# Macrophage response specificity to ligand mixtures is improved by signaling pathway antagonism

**DOI:** 10.1038/s44320-026-00230-9

**Published:** 2026-07-29

**Authors:** Xiaolu Guo, Supriya Sen, Julian Gonzalez, Alexander Hoffmann

**Affiliations:** 1 Department of Microbiology, Immunology, and Molecular Genetics, and Institute for Quantitative and Computational Biosciences, University of California, Los Angeles, 611 Charles E. Young Dr E, Los Angeles, CA USA; 2 Present Address: Amgen, One Amgen Center Drive, Thousand Oaks, USA

**Keywords:** Immunology, Signal Transduction

## Abstract

Macrophages function as immune sentinels that distinguish diverse threats and mount appropriate responses. This stimulus-response specificity (SRS) is partly encoded in the dynamics of the NFκB transcription factor. While most studies examine single ligands, physiological exposures involve complex multi-ligand mixtures. Using a mathematical model that captures heterogeneous single-cell NFκB responses, we generated simulation datasets for mixtures of up to five ligands and validated key predictions with live-cell microscopy. Following iterative refinement of the model, we quantified SRS with Wasserstein distance and machine learning classification and found that NFκB temporal coding can partially convey the presence of specific ligands within mixtures. By generating simulation datasets across all ligand pairs at doses spanning the full responsiveness range, we found several cases of synergy and antagonism between stimuli. Antagonism was for example the result of a limited supply of stimulus-cofactor CD14 or endosomal transport capacity. Synergy depended on ultra-sensitive IKK activation in cells with low receptor expression. While synergy does not enhance SRS, antagonism between TLR9 and TLR3 signaling pathways due to endosomal transport competition may enhance the distinguishability of CpG-pIC from ligand mixtures. These studies therefore identified antagonism mechanisms in signaling pathways as key to maintaining immune specificity under complex conditions.

## Synopsis

This study developed computational simulations of single-macrophage NFκB dynamics in response to ligand combinations, revealing that macrophages may partially detect the identity of specific ligands within ligand mixtures.

- A workflow was developed to predict NFκB responses to any ligand and dose combination in macrophages and validated by experimental data of key conditions.
- Dose-dependent synergistic and antagonistic regimes were identified for each ligand pair, along with their molecular mechanisms.
- An unanticipated mechanism by which CpG and pIC signal antagonistically was identified: limited capacity for endosomal transport.
- Macrophage NFκB signaling dynamics preserve partial stimulus specificity in complex ligand-mixture contexts, and this is fueled by signaling antagonism.

## Abstract (web summary)

This study developed computational simulations of single-macrophage NFκB dynamics in response to ligand combinations, revealing that macrophages may partially detect the identity of specific ligands within ligand mixtures.

## Graphical abstract

**Figure:**
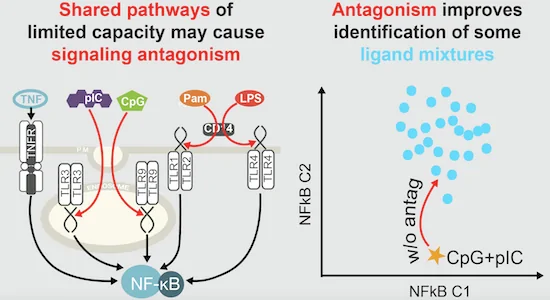

**Figure:**
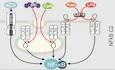

## Introduction

Functioning as sentinel immune cells, macrophages detect diverse immune threats by recognizing pathogen-associated molecular patterns (PAMPs), damage-associated molecular patterns (DAMPs) and cytokines (Kawai and Akira, 2011; Newton and Dixit, 2012). In response, they initiate immune defenses tailored to the specific threat, including cell-intrinsic defenses, localized immune responses, or systemic immune activation (Sheu et al, 2019; Sheu and Hoffmann, 2022). Indeed, stimulus-response specificity (SRS) is thought to be a hallmark of healthy macrophage function (Sheu and Hoffmann, 2022). The regulatory machinery governing stimulus-specific functions converges on a few signaling pathways, such as IRF, AP1, NFκB, and p38 (Luecke et al, 2021). Among these, NFκB is a key transcription factor that responds to all pathogens in macrophages and regulates immune gene expression.

The temporal dynamics of nuclear NFκB activity has been shown to mediate SRS following exposure of macrophages to cytokines or immune threats, a phenomenon known as temporal coding in signaling pathways (Selimkhanov et al, 2014; Kellogg et al, 2017; Lane et al, 2019; Adelaja et al, 2021; Rahman et al, 2024). Upon stimulation, NFκB translocate into the nucleus, where its activity is highly dynamic (Hoffmann et al, 2002; Nelson et al, 2004; Ashall et al, 2009; Sung et al, 2009; Tay et al, 2010), activating immune gene expression (Hoffmann et al, 2002; Werner et al, 2005; Lee et al, 2014; Sen et al, 2020; Cheng et al, 2021; Ando et al, 2021). NFκB activation exhibits stimulus-specific dynamic responses (Covert et al, 2005; Werner et al, 2005); for example, TNF-induced NFκB dynamics are oscillatory, while PAMP-induced responses are generally non-oscillatory. Such dynamic features can be quantified by six signaling codons: activation speed, peak amplitude, duration above a low threshold, total integral activity, how much of it is front-loaded (early versus late), and oscillatory content (Adelaja et al, 2021). These signaling codons were algorithmically identified as the features that provide the most information about stimulus ligand identity and dose. Dysfunction in NFκB stimulus-response specificity has been associated with diseases such as Sjögren’s syndrome (Adelaja et al, 2021).

While experimental studies of stimulus-response have typically focused on single, chemically defined ligands (Tay et al, 2010; Cheong et al, 2011; Selimkhanov et al, 2014; Adamson et al, 2016; Zhang et al, 2017; Adelaja et al, 2021; Rahman et al, 2024), Ligand mixtures more accurately represent the physiological environments encountered by macrophages. For example, BCG vaccines can activate immune cells via TLR2 and TLR4 (Heldwein et al, 2003). Similarly, *E. coli* infections may lead to multiple PAMPs being presented to macrophages, starting with LPS from the bacterial cell wall (Aderem and Ulevitch, 2000), followed by CpG, a DNA fragment from the E. coli genome (Bauer et al, 2001), with further exposure to cytokines such as TNF. In addition to single-pathogen infections presenting multiple PAMPs, DAMPs, and cytokines simultaneously, more complex scenarios involving multiple microbes may also occur. For instance, macrophages constantly encounter diverse bacteria and viruses in the colonic niche (Hegarty et al, 2023; Bain and Mowat, 2014), and co-infections in the lung can also lead to complex ligand mixtures. To probe such physiological scenarios, prior studies have used experimental approaches that focus on a limited number of representative combinations, such as LPS-TNFα (Gutschow et al, 2019) or LPS-Pam stimulation (Kellogg et al, 2017), or on selected five cytokine mixtures (Wang et al, 2021) that modulate NFκB or ERK dynamics. However, due to the vast exploration space of possible ligand combinations, there is a lack of systematic experimental investigation of SRS in response to ligand mixtures.

Given established mathematical models of NFκB regulatory mechanisms, a dearth of experimental data may be complemented by computational simulations if they faithfully recapitulate the heterogeneous single-cell stimulus-response trajectories observed in the experimental data that is available. Early models captured bulk-average NFκB dynamics in different IκB knockout genotypes (Hoffmann et al, 2002), and explored the mechanisms that could account for single-cell trajectories (Nelson et al, 2004; Sung et al, 2009; Hughey et al, 2015; Adelaja et al, 2021). Subsequent modeling efforts have been made to account for the cell-to-cell-heterogeneity through parameter selection. These mechanistic models, which integrate extensive experimental data of protein interactions, translocation, or catalysis, have provided confidence in the accuracy of the underlying biochemical reaction network.

Building on this foundation, the current state-of-the-art mechanistic model was developed and parameterized to recapitulate representative cell NFκB dynamics (Adelaja et al, 2021). This model comprises five receptor modules corresponding to the TNFR, TLR2, TLR9, TLR4, and TLR3 signaling pathways. These receptor modules converge on a shared core module, which consists of the NFκB-IKK-IκBα negative feedback loop. The five receptors interact with specific ligands: the cytokine tumor necrosis factor (TNF, for TNFR); Pam3CysSerLys4 (Pam, for TLR2), cytosine-phosphate-guanine (CpG, for TLR9), and lipopolysaccharide (LPS, for TLR4), which are bacterial pathogen-associated molecular patterns (PAMPs); as well as polyinosinic:polycytidylic acid (pIC, for TLR3), a viral PAMP. The topology of this NFκB mathematical model and its parameterization are grounded in decades of experimental research and are supported by numerous published studies (Hoffmann et al, 2002; Basak et al, 2012). Based on this, we have recently developed model simulations of a population of heterogeneous single-cell NFκB signaling trajectories (Guo et al, 2025). A quantitative assessment of this model demonstrates that it successfully captures the experimental SRS, as determined by information-theoretic and machine learning classification methods, for single-ligand stimuli while accounting for cellular heterogeneity within the population.

Here, we have leveraged these advances to study NFκB response trajectories to combinatorial ligands. We developed a workflow to generate a population of virtual NFκB response cells that are capable of responding to multiple ligands by matching receptor proximal signaling modules so that available data distributions are preserved. Comparing model simulation with available experimental validation data allowed us to improve the model iteratively and gain new insights into the regulatory mechanism. Remarkably, we found that even within complex mixtures, the presence of specific ligands is partially identifiable based on ligand-characteristic NFκB dynamics. Examining the underlying regulatory mechanism revealed that the SRS of ligand mixtures is enhanced by nonlinear signal combining due to pathway antagonism at common signaling nodes. This insight describes a generalizable principle of dynamic signal coding to maintain specificity.

## Results

### Predicting NFκB responses to combinatorial ligand stimuli

To study NFκB response dynamics in response to stimulation with combinatorial ligands, we employed a well-parameterized single-cell mechanistic model of the NFκB signaling network (Guo et al, 2025). This mechanistic model was previously trained on single-ligand response experimental datasets, capturing the single-ligand stimulus-response specificity of the population of macrophages while accounting for cellular heterogeneity (Guo et al, 2025). Specifically, quantitative NFκB dynamic trajectory data from hundreds of single macrophages responding to five single ligands (TNF, pIC, Pam, CpG, and LPS) at three to five doses was obtained from live-cell imaging experiments. The mathematical model (Appendix Fig. S1) consists of a 52-dimensional system of ordinary differential equations, including 52 intracellular species, 101 reactions and 133 parameters, and is divided into five receptor modules, which respond to the corresponding ligands respectively, and the IKK-NFκB core module that contains the prominent IκBα negative feedback loop. By fitting the single-cell experimental dataset with a nonlinear mixed-effect statistical model (coupling with a 52-dimensional NFκB ODE model), the parameter distributions for the single-cell population were inferred. Analyzing the resulting simulated NFκB trajectories with information-theoretic and machine learning classification analyses confirmed that the virtual cell model simulations reproduced key SRS performance characteristics of live macrophages.

**Figure 1:**
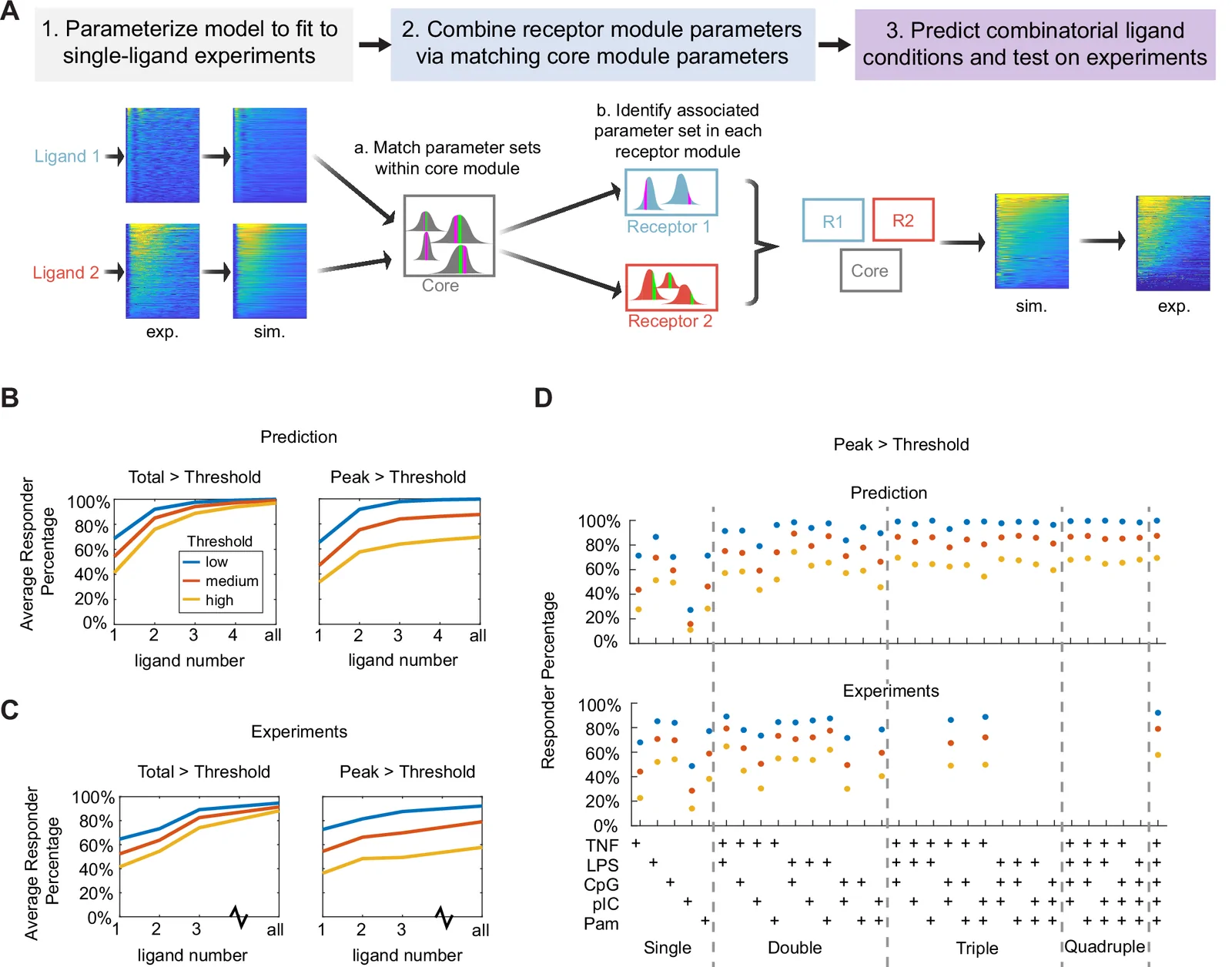
Extending a single-ligand-trained mathematical model to predict NFκB responses to ligand mixtures. (**A**) Schematic of the workflow: A mathematical model is parameterized to account for heterogeneous NFκB signaling dynamics in response to five single-ligand stimulation conditions. This involves parameters within the regulatory networks of the ligand-specific receptor module and the shared IKK-IκB-NFκB core module. By matching similar core module parameter sets, associated parameter sets for the two receptor modules are combined, thereby enabling simulations of NFκB responses to ligand mixtures. (**B**) Model-predicted average responder fraction for all single, double, triple, quadruple or the five ligand stimulations. Responder fractions are defined by low, medium or high thresholds applied to either Total (left) or Peak (right): thresholds of 0.4, 0.5, and 0.6 µM·h for Total, and 0.12, 0.14, and 0.16 µM for Peak. (**C**) Experimentally determined average responder fractions for the single, double, triple, quadruple or all ligand stimulations. Responder fractions are defined by low, medium or high thresholds applied to either Total (left) or Peak (right) using arbitrary fluorescence units (AU): thresholds of 0.2, 0.3, and 0.4 AU·h for Total, and 0.14, 0.18, and 0.22 AU for Peak are applied. (**D**) Responder fractions defined by a low, medium, or high threshold applied to Peak activity for each indicated ligand and ligand combination, for simulation (top) and experimental data (bottom). Responder fractions are defined as in (**B**, **C**). These data define the averages shown in (**B**, **C**). Source data are available online for this figure.

We developed a workflow to predict NFκB response trajectories in response to combinatorial ligand stimulation from this single-ligand parameterized model. Previous work determined parameter distributions for only the cognate receptor module (and the core module) that provided the best fit for the single-ligand experimental data (Fig. 1A, Step 1), and other receptor module parameter information is missing. To simulate stimulus responses to more than two ligands, we imputed the other ligand–receptor module parameters using shared core-module parameters as common variables and employing nearest-neighbor hot-deck imputation (Andridge and Little, 2010). In this setup, the core module functions as an “anchor” to harmonize two or more receptor-specific parameter distributions. This was achieved by minimizing Euclidean distance between the core module parameters associated with the independently parametrized single-ligand models (Fig. 1A, Step 2). Specifically, for a given cell i responding to ligand l ($i\in {S}_{l}$), we identified a corresponding cell j responding to ligand k ($j\in {S}_{k}$) through the following optimization:

1 $${j}_{i,{S}_{k}}={{{{\rm{argmin}}}}}_{j\in {S}_{k}}{||}{p}_{{core}}^{\left(j\right)}-{p}_{{core}}^{\left(i\right)}{||}$$

where ${p}_{{core}}^{(i)}$ represents the core parameter vector of cell i. The parameters of cell i in the receptor module associated with ligand k (hereafter referred to as receptor module k) were then inferred as:

2 $${\widetilde{p}}_{{S}_{k}}^{\left(i\right)}={p}_{{S}_{k}}^{({j}_{i,{S}_{k}})}$$

where ${p}_{{S}_{k}}^{({j}_{i,{S}_{k}})}$ represents the parameters of receptor module k for cell ${j}_{i,{S}_{k}}$, and ${\widetilde{p}}_{{S}_{k}}^{\left(i\right)}$ represents the extended receptor module k parameters of cell i.

**Figure EV1:**
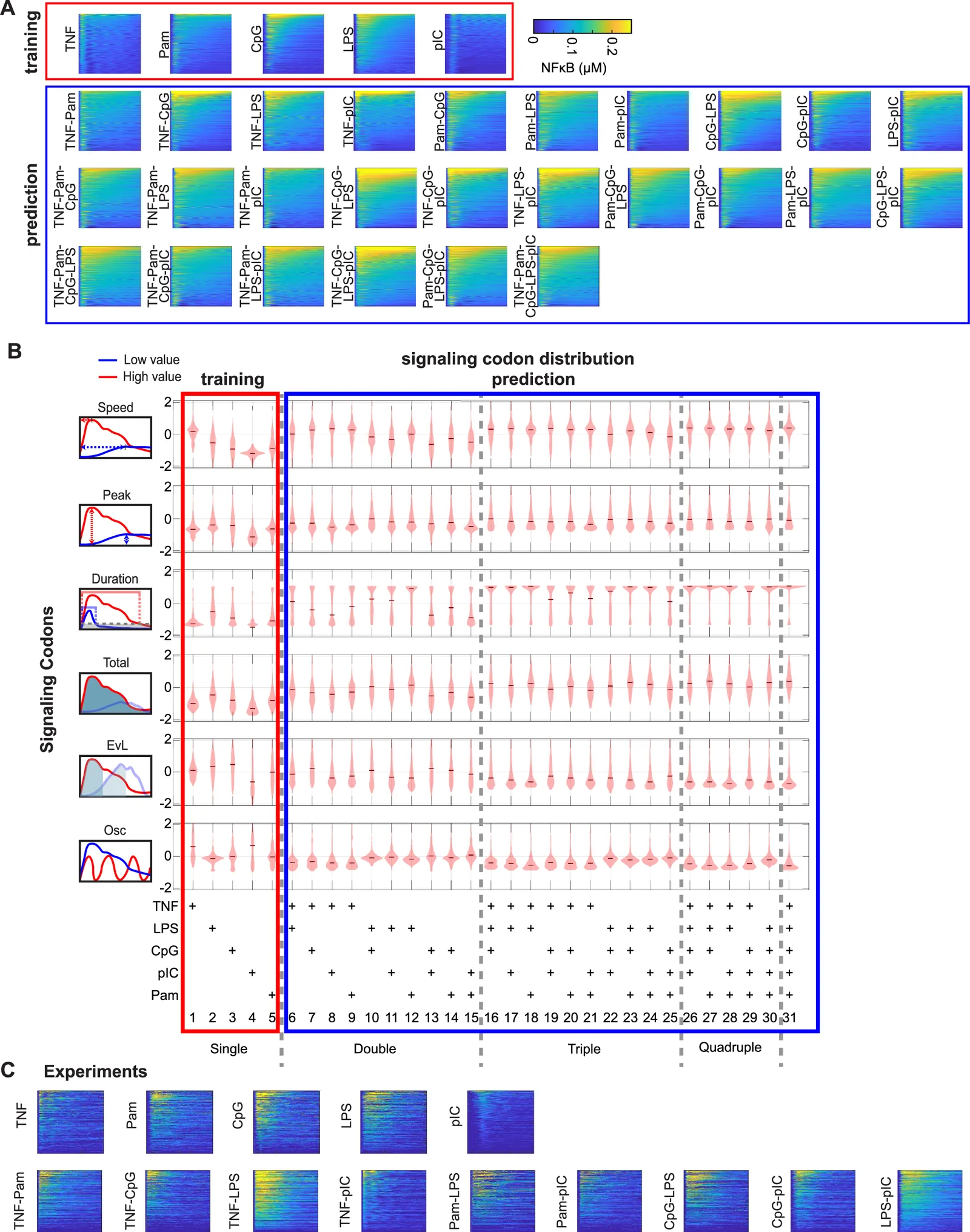
Model simulations predict NFκB responses to combinatorial ligand stimulation. (**A**) Heatmaps of simulated single-cell NFκB trajectories under single-ligand (training dataset, in the red box) and combinatorial ligand (prediction dataset, in the blue box) stimulation. For each heatmap, the condition is indicated on the left, and each row within the heatmap represents a single-cell prediction, ordered by the total activity of the signaling. In each subpanel, the x-axis represents time, and color intensity indicates NFκB abundance in accordance with the color bar. (**B**) Violin plots of signaling codon distributions from single-ligand stimulation (training) to combinatorial ligand stimulation (prediction). On the left, plots illustrate the high and low values for each signaling codon to illustrate the dynamic features each signaling codon captures and quantifies. Conditions are labeled at the bottom. The mean value of the respective signaling codon distribution is indicated with “-“. The red box indicates single-ligand conditions (training dataset), and the blue box indicates the combinatorial-ligand conditions (prediction dataset). For each condition, *n* = 1000 simulated single cells. (**C**) Heatmaps of experimental single-cell NFκB trajectories under single-ligand (top row) and pairwise combinatorial ligand (bottom row) stimulation. Within each heatmap, each row represents a single-cell prediction (ordered by total signaling activity); the x-axis represents time, and color intensity indicates NFκB abundance in accordance with the color bar in panel (**A**).

Using this approach, we simulated NFκB signaling responses not only in response to five single-ligand stimuli (TNF, Pam, CpG, LPS, and pIC), but also ten double-ligand combinations, ten triple-ligand combinations, five quadruple-ligand combinations, and one quintuple-ligand condition—a total of 31 conditions. The model was trained exclusively on single-ligand data (red box, Fig. EV1A) and was then able to predict responses to combinatorial ligands (blue box, Fig. EV1A).

To quantify NFκB dynamics, we decomposed trajectories into six signaling codons, which were identified as the informative features of NFκB trajectories (Adelaja et al, 2021). Specifically, Speed represents how quickly NFκB becomes activated (translocating to the nucleus), Peak denotes the maximum value of the dynamic response, Duration measures how long the signal remains above a low activity threshold, Total represents the integral of the signaling dynamics over an 8-hr timecourse, EvL measures whether the activity is loaded early or late, and Osc quantifies the oscillatory content (Fig. EV1B, see methods for detailed signaling codon definition). Heatmaps and signaling codon distributions revealed that combinatorial-ligand conditions were generally predicted to exhibit stronger responses (Total) compared to single-ligand conditions (Fig. EV1B).

### Responder fractions increase with the number of ligands in combinatorial stimuli

To test our model predictions, we conducted experimental studies for a subset of the combinatorial stimuli (Fig. EV1C). Both simulations and experimental data reveal the presence of non-responding cells under single-ligand conditions (Fig. EV1). We asked: do non-responders observed experimentally arise from cellular ill-health, or are they due to cellular heterogeneity in the receptor modules that may render some cells insensitive to one or more ligands but not all?

To address this question, we first defined the responder ratio as the fraction of cells with Peak or Total NFκB signaling codons exceeding predefined thresholds. These thresholds were chosen to yield a responder fraction of 40–80% (Singh et al, 2024) for single-ligand stimuli. The model predicted that the responder fraction generally increases with the number of combinatorial stimuli (Fig. 1B). When applying thresholds on Total activity, the average responder ratio reaches a saturation point of over 90% that is largely independent of the threshold applied. When applying thresholds on Peak activity, the average responder fraction also increases, but the plateau is sensitively dependent on the threshold. We analyzed the experimental data analogously (Fig. 1C) and found remarkable concordance; the responder fraction increases with the number of ligands used, reaching a plateau that is either independent of threshold (Total) or dependent on threshold (Peak). This analysis provides confidence in the validity of the model as well as the experimental workflow. It suggests that signaling from multiple ligands generally combines in an integrative manner, and that non-responder cells are likely a result of cellular heterogeneity in receptor modules rather than cellular ill-health that may affect the core IKK-NFκB module.

Examining the individual stimulus conditions in detail, we found that the responder fraction varies substantially between ligands (e.g., lowest for pIC and highest for LPS) as well as between ligand mixtures (Fig. 1D; Appendix Fig. S2). For the available experimental data, we found good agreement with the model predictions. Some discrepancies between simulations and experiments were observed. These were cases of non-integrative responses, where the combinatorial-ligand response is lower than at least one of the corresponding single-ligand responses. For instance, the model predicted that CpG-pIC would exhibit a higher response than exposure with CpG or pIC alone (Fig. 1D, top panel). However, experimental results showed a non-integrative response for the CpG-pIC pair, with lower activation than CpG alone. This was in contrast to the ligand-pair pIC-Pam, which involves analogous TRIF and MyD88 signal transducers (Fig. 1D, bottom panel).

### Limited endosomal transport can result in ligand antagonism

**Figure 2:**
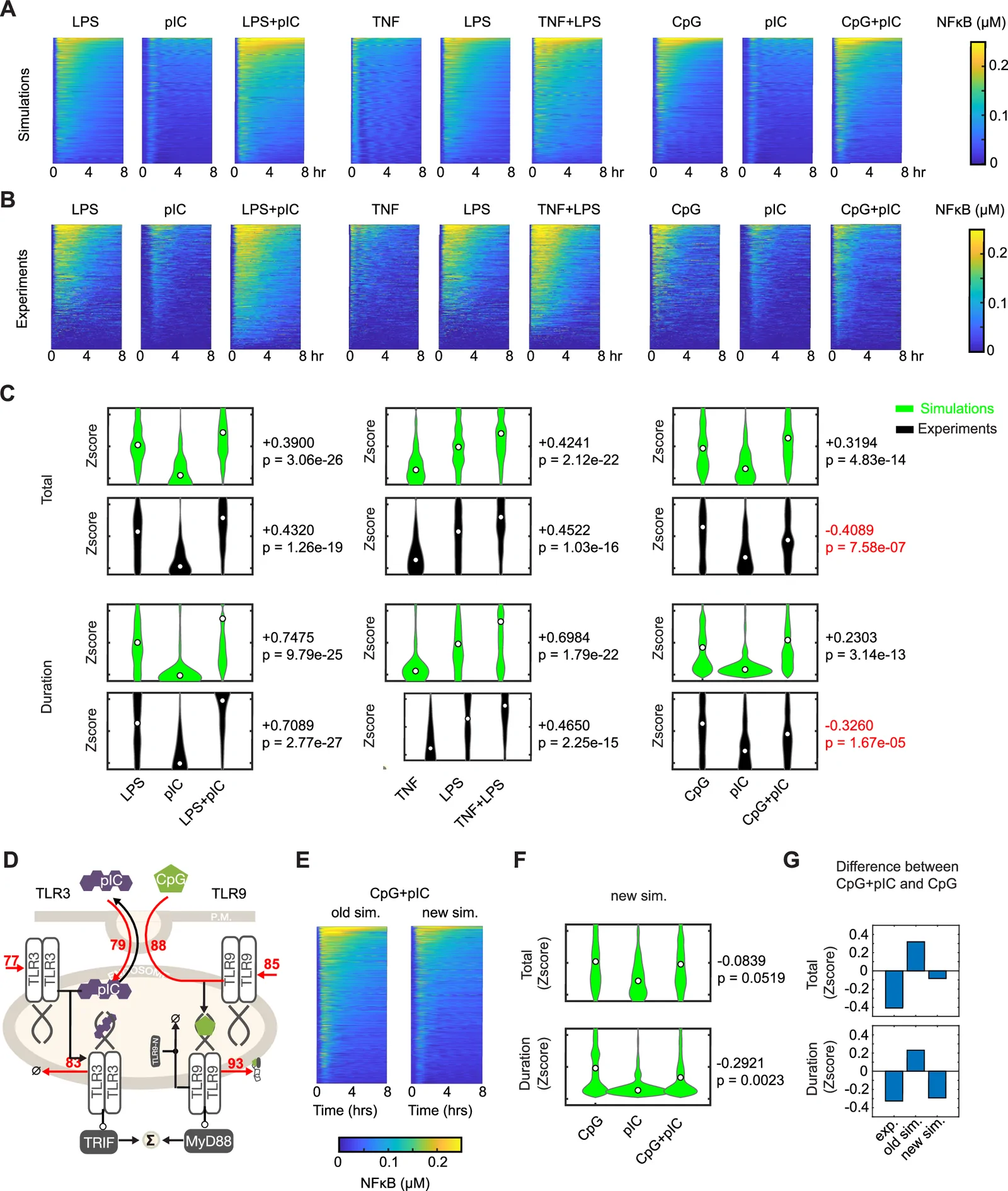
Iterative modeling and experimentation of combinatorial ligand stimulation. (**A**) Heatmaps of model-predicted NFκB signaling trajectories in macrophages responding to combinatorial ligand stimulation: LPS+pIC, TNF + LPS, and CpG + pIC. Trajectories were ordered by total integral with nuclear NFκB abundance over the time course, as indicated by the color scale. (**B**) Heatmaps of experimental data for the conditions shown in (**B**). (**C**) Violin plots comparing signaling codon distributions of total (top) and duration (bottom) from single and combinatorial ligand stimulation, as predicted by the model (green) and determined by experiments (black). The hollow circle in each plot indicates the distribution median. Values shown to the right of each panel indicate the median difference between the combinatorial condition and the larger median of the two corresponding single-ligand conditions. Positive values indicate that the combinatorial response exceeds both single-ligand responses, whereas negative values indicate that the combinatorial response is lower than the larger single-ligand response. Associated *p* values were calculated using one-sided Mann–Whitney *U*-tests. For each condition, *n* = 1000 simulated single cells (model) and *n* ≈ 600–950 experimental single cells (exact n per condition in the Source Data). (**D**) Schematic of the regulatory network in which CpG and pIC use the same endosomal transport machinery. The potential saturation of endosomal transport may lead to functional antagonism between the two ligands. (**E**) Heatmap of predicted NFκB signaling trajectories in macrophages upon combinatorial stimulation with CpG and pIC under transport saturation conditions. (**F**) Violin plots of the z-scored signaling codon distributions of total (top) and duration (bottom) as predicted by the refined model for single and combinatorial ligand stimulation. Values shown to the right of each panel indicate the median difference and *p* values (same as Fig. 2C). For each condition, *n* = 1000 simulated single cells. (**G**) Bar graph of the mean value difference in the z-scored total (left) and duration (right) signaling codon distributions shown in panel (**C**) for the original model and in panel (**E**) for the refined model. Source data are available online for this figure.

To investigate the discrepancy in more detail, we examined the simulated and experimentally measured trajectories of TNF + LPS, pIC + LPS, and pIC + CpG dual-ligand simulations and corresponding single-ligand stimulations (Fig. 2A). Experiments showed good concordance for LPS+pIC and TNF + LPS, but not CpG + pIC, which showed much lower responses than predicted (Fig. 2B). By comparing signaling codon distributions calculated for simulated and experimental trajectories (Appendix Fig. S3), we similarly found good general agreement, although some discrepancies, such as for the CpG-pIC pair, were evident. Specifically, we found that LPS + pIC, and TNF + LPS responses to be integrative as predicted (Fig. 2C). However, the CpG+pIC stimulated response was non-integrative: both Duration and Total were lower for CpG+pIC than for CpG alone, in contrast to model predictions.

To address the model’s shortcomings, we noted that both ligands are sensed by receptors located in endosomes. The endosomal transport machinery is therefore responsible for moving both ligands towards the receptors (Hu et al, 2015; O’Sullivan and Lindsay, 2020), but the model represented these as independent mass action reactions. Modeling receptor pathways independently could not account for their shared limited endosomal capacity. To address this, we reformulated the model such that CpG and pIC are transported by a common endosomal trafficking process (Fig. 2D), such that when one ligand saturates the transport machinery, the other ligand is affected. This biochemical process is captured by a Michaelis–Menten kinetics formulation (see Methods for details) and does not require introducing additional parameters.

The updated model resulted in a negative correlation between endosomal CpG-receptor and pIC-receptor complexes due to competition for the limited endosomal transport of the ligands. The fact that pIC-TRIF signaling is generally weaker than CpG-MyD88 signaling allows pIC to “poison” CpG-induced NFκB activation. Simulations with the revised model accurately reflected the non-integrative CpG+pIC response (Fig. 2E), where Duration and Total activity for the combined stimuli were lower than for CpG alone (Fig. 2F,G). In sum, our results suggest that the saturability of endosomal transport may lead to non-integrative signaling responses for stimulus ligands that are sensed by endosomal receptors.

### Stimulus-response specificity for combinatorial ligand stimuli

**Figure EV2:**
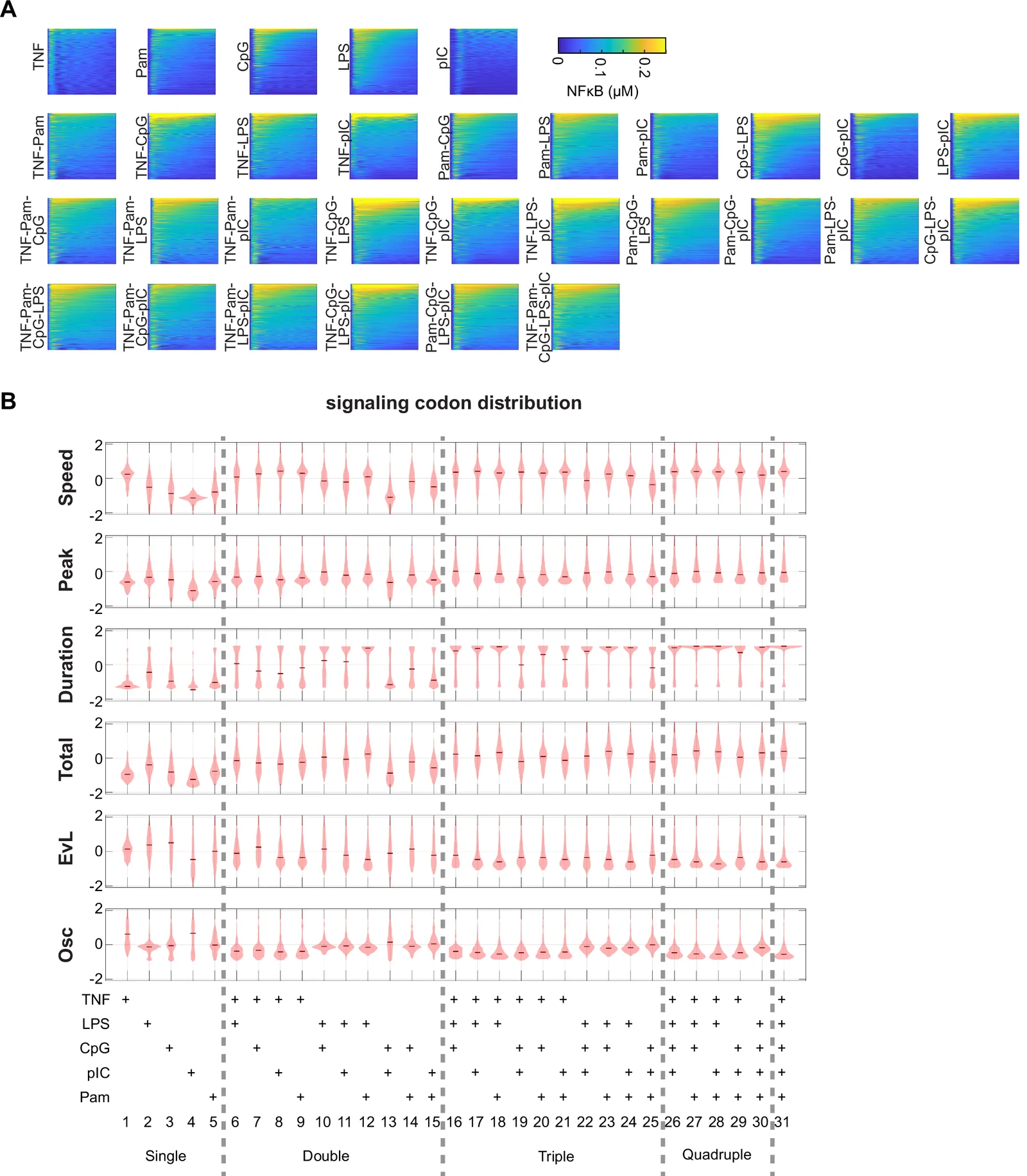
Updated model simulations of combinatorial ligand stimulations. (**A**) Heatmaps of simulated single-cell NFκB trajectories under single and all combinations of ligand stimulation using the updated model (Fig. 2D). For each heatmap, the condition is indicated on the left, and each row within the heatmap represents a single-cell prediction, ordered by Total signaling activity. In each subpanel, the x-axis represents time, and color intensity indicates NFκB abundance in accordance with the color bar. (**B**) Violin plots of signaling codon distributions from signaling trajectories shown in panel (**A**). Conditions are labeled at bottom. The mean value of the respective codon distribution is indicated. For each condition, *n* = 1000 simulated single cells.

We employed the updated model to simulate NFκB signaling dynamics and decomposed the trajectories into signaling codons (Fig. EV2A,B) to enable investigations of the stimulus-response specificity (SRS). For simplicity, we assume that high-dose ligands and their combinations represent critical immune challenges, and we evaluate whether cells can achieve SRS for these challenges, whereas low-dose stimuli may be perceived as noise or as non-critical challenges. Previous studies have shown that single-ligand stimulation yields high SRS at high doses (Adelaja et al, 2021). We employed three approaches to assess SRS: (1) Wasserstein distance of signaling codon distributions between conditions; (2) random forest classifier using signaling codons; and (3) long short-term memory (LSTM) classifier using time-series data.

**Figure 3:**
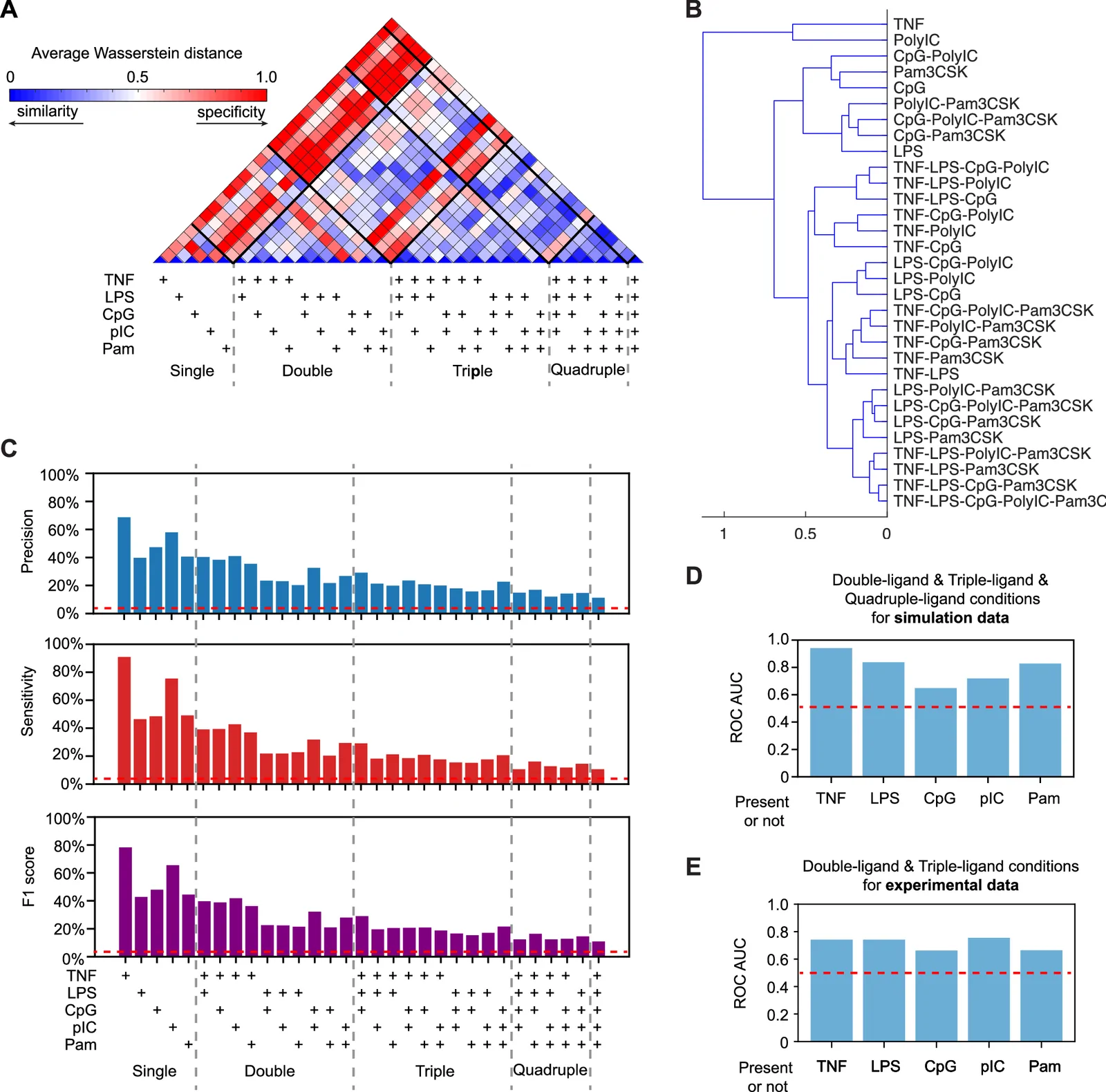
Distinguishability of single-ligand and combinatorial ligand stimuli. (**A**) Heatmap of average Wasserstein distance between the distributions of six z-scored NFκB signaling codons for each of 31 conditions. Conditions are identified on the diagonal axis. Color scale indicates average Wasserstein distance low values (blue, similar) to high values (red, specific). (**B**) Hierarchical clustering of the 31 conditions *via* single-linkage clustering of the average Wasserstein distance metric. (**C**) Bar plots of precision, sensitivity, and F1 score of a Random Forest classifier trained on all 31 conditions using NFκB response signaling codons as input to predict the stimuli. 67% of the data were used for training and 33% for testing. (**D**) Bar plot depicting the ROC AUC of a classifier trained on the signaling codons distributions of simulation data for TNF-present vs. TNF-absent, LPS-present vs. LPS-absent, CpG-present vs. CpG-absent, pIC-present vs. pIC-absent, and Pam-present vs. Pam-absent groups generated from all double-ligand, triple-ligand, and quadruple-ligand conditions. Methodology as in (**C**). (**E**) Bar plot depicting the ROC AUC of a classifier trained on the signaling codons distributions of experimental data. Methodology as in (**D**). Source data are available online for this figure.

We first calculated the Wasserstein distance between signaling codons in different stimulus conditions, as this metric has been used to quantify the similarity and specificity across conditions (Guo et al, 2025). This confirmed a high distinguishability for single-ligand stimuli but revealed that the distinguishability diminishes with the number of combinatorial ligands that make up the stimulus condition (Fig. 3A). To rank the most distinguishable stimuli among ligand mixtures, we applied hierarchical clustering to all 31 conditions based on their Wasserstein distances (Fig. 3B). TNF was the most distinguishable ligand in context of all possible ligand mixtures (with hierarchical height ≈0.5 to the nearest cluster), followed by the viral PAMP pIC (hierarchical height ≈0.49 to the nearest cluster). Bacterial PAMPs (LPS, CpG, and Pam) are less distinguishable compared to TNF or pIC, but still distinguishable from other mixtures of ligands (average hierarchical height ≈0.3 to the corresponding nearest cluster). Overall, single-ligand conditions were more specific than combinatorial-ligand conditions.

To further examine SRS in ligand mixtures using the updated model simulation, we trained random forest classifier (Breiman, 2001) on the signaling codon distributions and quantified the specificity for each condition via different prediction accuracy metrics. Machine-learning classifiers have been widely used to assess the distinguishability and confusion in signaling dynamics (Adelaja et al, 2021; Rahman et al, 2024). Analysis of the classifier’s precision and sensitivity matrix showed that true positive rates (diagonal elements, quantifying specificity) were substantially higher than misclassification rates (off-diagonal elements, quantifying the confusion between conditions) (Appendix Figure S4A, B). When plotting only the true positives, single-ligand conditions demonstrated the highest sensitivity, precision, and F1 scores, while distinguishability declined with increasing ligand numbers (Fig. 3C; Appendix Fig. S4A,B). Notably, the TNF-stimulated condition achieved the highest distinguishability with approximately 69% precision, 91% sensitivity, and a 78% F1 score, whereas the five-ligand combination condition exhibited the lowest distinguishability (11% for sensitivity, precision, and F1 score), though still far above the random guess baseline of 3.2% (red dashed line in Fig. 3C). We also applied a LSTM machine learning model to the entire time-series trajectories (Hochreiter & Schmidhuber, 1997; Singh et al, 2024), and found similar results, confirming signaling codons capture all the information present in the newly simulated data (Appendix Fig. S5).

We then used random forest classifiers to investigate whether dual-ligand conditions show confusion with the corresponding single-ligand conditions. For each ligand pair, a model was trained to distinguish among three conditions: ligand A alone, ligand B alone, and the combined stimulus (ligand A + B). While dual-ligand stimuli were less distinguishable than their respective single-ligand counterparts, they remained well differentiated, with sensitivities ranging from 60 to 97% (Appendix Fig. S4C) and precisions from 58 to 94% (Appendix Fig. S4D), significantly outperforming the 33% expected from random guessing (red dashed line in Appendix Fig. S4C,D). Among all pairs, the TNF-alone condition exhibited the highest specificity in the TNF-related pairs, achieving over 94–95% sensitivity and 85–91% precision. TNF stimulation remained the most distinguishable, even from TNF-involved dual-ligand combinations.

We reasoned that ultimately, the cell needs to determine whether specific immune stimuli are present. To test this, we grouped all double-ligand, triple-ligand, and quadruple-ligand conditions (in total 25 conditions) into two categories: one with 14 conditions where TNF is present and another with 11 conditions where TNF is absent. We then trained machine learning classifiers to predict whether a cell was exposed to TNF based on its NFκB signaling codon. Classifiers trained to distinguish TNF-present from TNF-absent conditions achieved a Receiver Operating Characteristic-Area Under the Curve (ROC AUC) of 0.94, significantly above the 0.5 baseline (Fig. 3D; Appendix Fig. S6A). Extending this analysis to other ligands, cells detected LPS (0.84), Pam (0.83), pIC (0.72), and CpG (0.65) in mixtures (Fig. 3D; Appendix Fig. S6A). Using experimental data from double- and triple-ligand stimuli (Fig. 1D), ROC AUC values were TNF 0.74, LPS 0.74, Pam 0.66, pIC 0.75, and CpG 0.66 (Fig. 3E; Appendix Fig. S6B). Classifier accuracies yielded consistent results (Appendix Fig. S6C,D). These results indicated a notable capability of preserving ligand-specific information within NFκB dynamics, potentially enabling nuclear detection of extracellular ligands even in complex stimulus mixtures.

### Ultrasensitive IKK activation underlies synergy among low-dose ligands

We wondered what may enable the preservation of distinctive dynamic signaling features when cells are exposed to ligand mixtures. To tackle this question more comprehensively, we considered the full range of ligand doses, ranging from a barely perceptible response to a saturated response. We reasoned that different doses of ligand combinations may elicit synergistic or antagonistic responses, potentially amplifying cellular outputs or optimizing decision-making under limited cellular resources. To determine whether ligands combine synergistically or antagonistically, we simulated 360,000 single-cell signaling trajectories across 360 conditions generated from ten ligand pairs, each evaluated at 6 doses spanning nonresponsive to saturating levels (Appendix Fig. S1; see Methods for details).

**Figure EV3:**
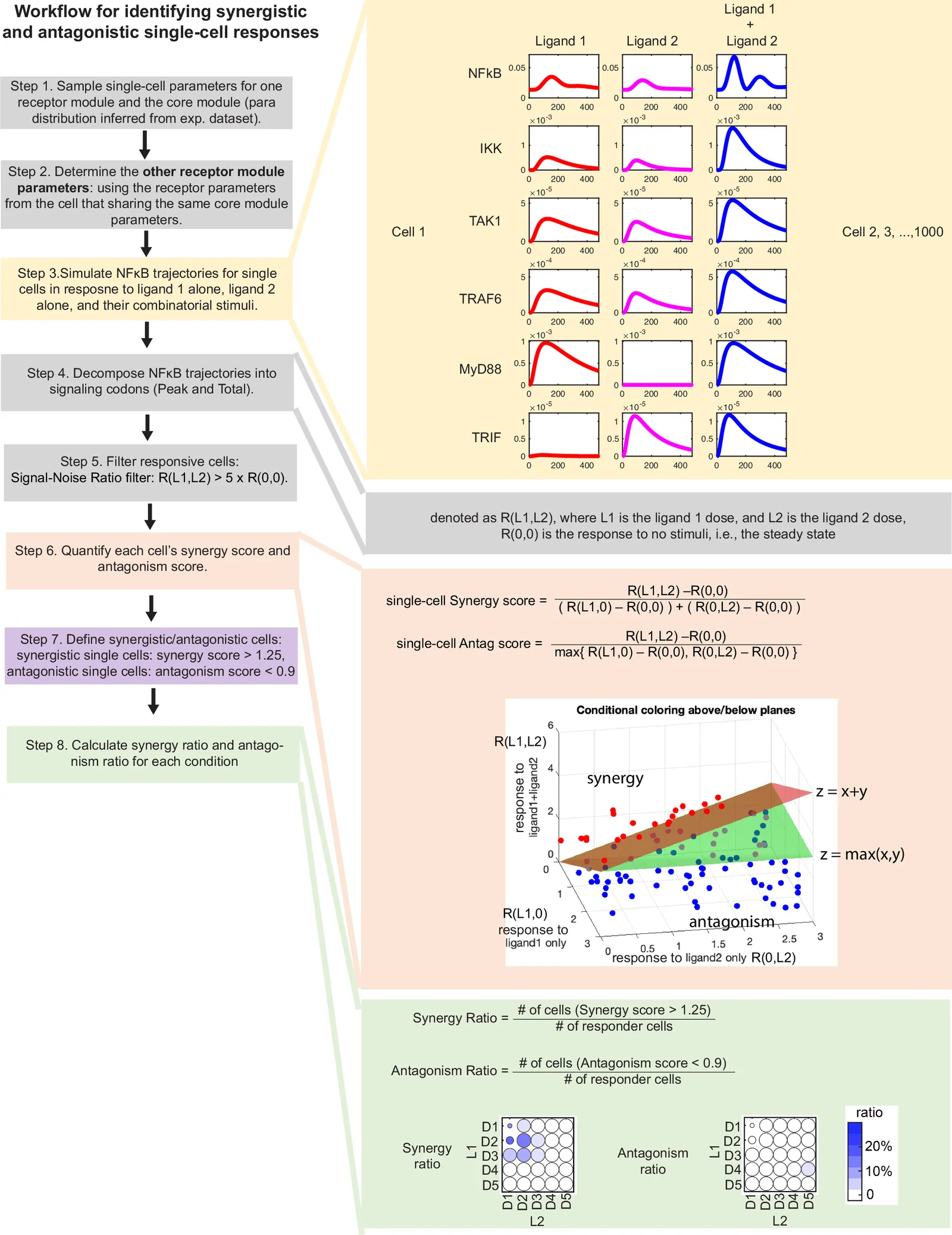
Workflow for simulating single-cell signaling dynamics and calculating the synergy and antagonism score. Left is the description of the workflow, and right are illustrations of key steps described in the workflow. Step 1 corresponds to the Methods section “Simulation of heterogenous NFκB dynamical responses”; Step 2 corresponds to the Methods section “Predicting heterogeneous single-cell responses to combinatorial-ligand stimulation”; Steps 3–7 correspond to the Methods section “Single-cell synergy and antagonism score”. On the right, the illustration for Step 3 shows the generated simulation for individual cells (cell 1 as an example; in total, we generated 1000 virtual cells for each ligand pair). Within the cell 1 results, in each subpanel, the x-axis represents time; the y-axis shows the simulated species (indicated on the left) under three example conditions: applying the first ligand only, applying the second ligand only, or applying their combination. Illustration for Step 6: the synergy and antagonism score equations are the same as in the main text, Results “Ultrasensitive IKK activation underlies synergy among low-dose ligands” and “Kinetic competition can lead to antagonism between high-dose ligands and enhance signaling specificity of ligand mixtures,” where R is the peak response for the corresponding stimuli (L1 represents the first ligand dose, L2 represents the second ligand dose; 0 represents the corresponding ligand not applied, i.e., 0 dose). Responses for the single-ligand conditions and the combinatorial condition are plotted in 3D space, with R(L1,0) − R(0,0) as the x-axis, R(0, L2) − R(0,0) as the y-axis, and R(L1, L2) − R(0,0) as the z-axis. The red dots illustrate synergistic cells, the blue dots illustrate antagonistic cells, and the gray dots illustrate non-synergistic/non-antagonistic cells. The two planes z = x + y and z = max(x,y) separate synergistic, antagonistic, and non-synergistic/non-antagonistic cells. Illustration for Step 8: for each ligand pair, the fractions of synergistic and antagonistic cells are calculated and plotted as bubble plots, where circle size indicates the number of responding cells, and color indicates the fraction of synergistic/antagonistic cells (Figs. 4A and 5A).

**Figure 4:**
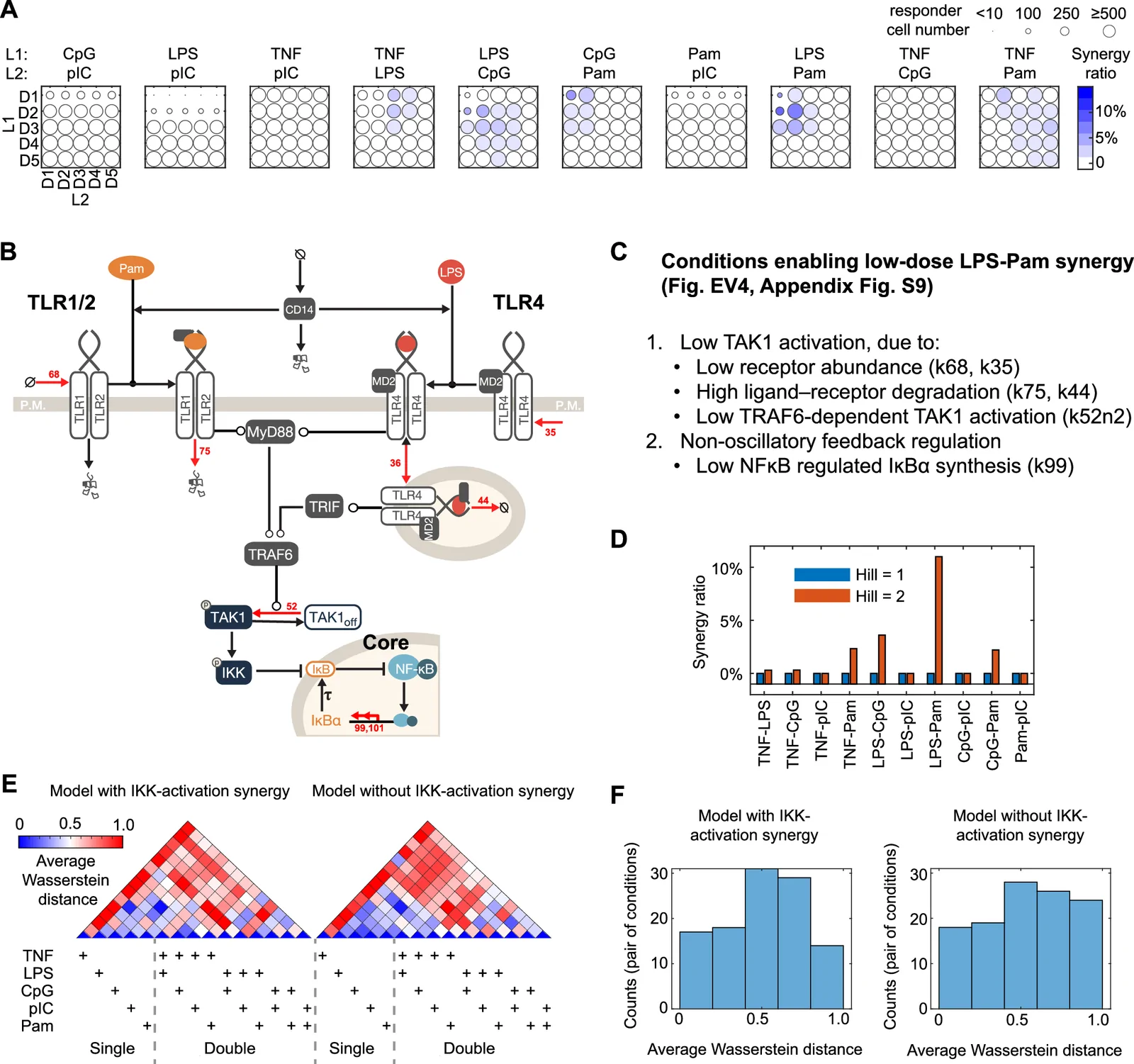
Evaluating dose-dependent synergy for ligand combinations and their stimulus-response specificity. (**A**) Bubble chart showing the fraction of cells exhibiting signaling synergy (color scale) and the number of responder cells (bubble size) across ten ligand pairs. Each panel is labeled at the top with Ligand 1 (L1) and Ligand 2 (L2). Dose levels for L1 and L2 are shown on the y-axis and x-axis, respectively (the first panel serves as an example; all others follow the same format). (**B**) Schematic of the NFκB signaling model for LPS-Pam signaling pathway, illustrating two receptor-specific modules alongside the central core (TAK1, IKK, and the NFκB–IκBα feedback loop). Red arrows with numeric labels mark the variable single-cell parameters. Figure elements are adapted from Adelaja et al, (2021) and Guo et al, 2025. (**C**) Summary of conditions enabling low-dose LPS-Pam synergy, as delineated in Fig. EV4; Appendix Fig. S9. (**D**) Bar plot showing the fraction of synergistic cells among dose-2 ligand pairs for Hill = 1 and Hill = 2 (corresponding to Appendix Fig. 11A,B). (**E**) Evaluating stimulus-response specificity of dose-2 ligands and ligand pairs: Heatmaps of average Wasserstein distances between the distributions of 6 NFκB signaling codons for each of 15 conditions (corresponding to Appendix Fig. S11C,D) using a model with IKK-activation synergy (Hill = 2) or without (Hill = 1). Conditions are identified on the diagonal axis. Color intensity indicates the average Wasserstein distance according to the color bar. (**F**) Histogram of the number of condition pairs (out of 120 total) for which the average Wasserstein distance falls within the indicated bin for the alternative models described in (**E**): model with IKK-activation synergy (left) and model without IKK-activation synergy (right). The y-axis indicates the number of condition pairs whose average Wasserstein distance falls within each bin. The *p* value shown in the upper right of the right panel was calculated using a paired Wilcoxon signed-rank test comparing the matched condition-pair distances between the two models. Source data are available online for this figure.

We quantified ligand-pair synergy using the peak of NFκB signaling dynamics (Fig. EV3). After excluding nonresponsive cells, we computed a single-cell synergy score, defined as

$${{{\rm{Synergy\; Score}}}}=\frac{R\left(L1,L2\right)-R\left(0,0\right)}{\left(R\left(L1,0\right)-R\left(0,0\right)\right)+\left(R\left(0,L2\right)-R\left(0,0\right)\right)}$$

where R is the Peak of the Response. Cells with a synergy score >1 represent the combined response $R\left(L1,L2\right)-R\left({{\mathrm{0,0}}}\right)$ exceeding the sum of single-ligand effects $R\left(L{{\mathrm{1,0}}}\right)-R\left({{\mathrm{0,0}}}\right)$ and $R\left(0,L2\right)-R\left({{\mathrm{0,0}}}\right)$. To avoid numerical artifacts, we classified cells as synergistic when the score was >1.25 (Fig. EV3, see Methods for more details). For each condition, we calculated the ratio of synergistic cells (Fig. EV3, Step 8). Synergy was most pronounced when one ligand was at a low or medium dose (Fig. 4A).

**Figure EV4:**
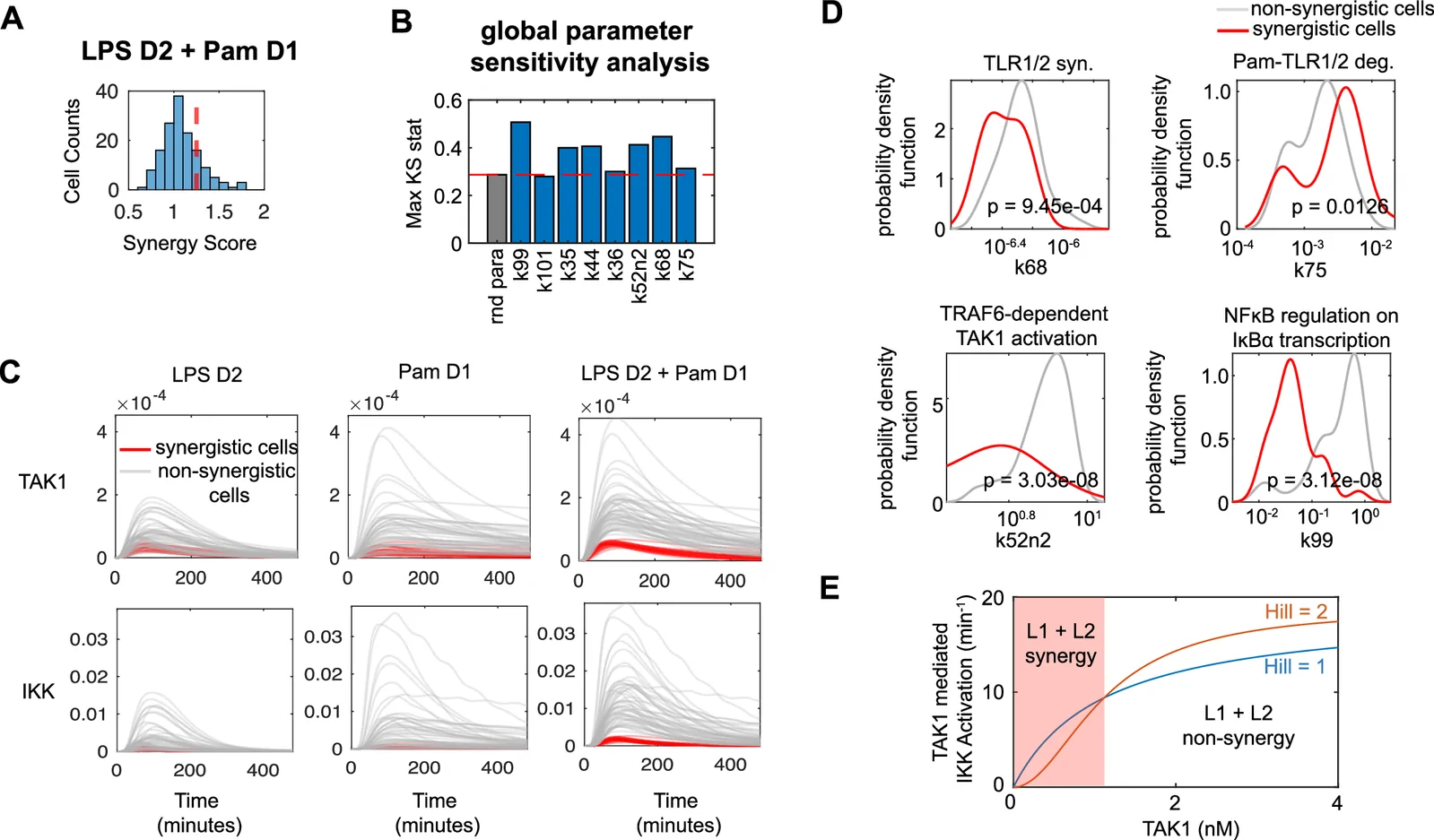
Conditions for single-cell synergy under LPS D2 + Pam D1 co-stimulation. (**A**) Histogram of synergy scores for responsive cells under combined stimulus conditions of Dose-2 (D2) LPS and Dose 1 (D1) Pam. (**B**) Bar plot indicating the feature importance score of each parameter, as evaluated via PAWN parameter analysis for the condition of D2 LPS + D1 Pam. The KS stat is the Kolmogorov–Smirnov distance between the unconditional output distribution and the output distribution conditioned on a given parameter, with a larger KS value indicating a stronger influence of that parameter on the model output. The parameter index is the same as in Fig. 4B. “rnd para” denotes a randomly assigned variable included in the model as a negative control, and its corresponding KS statistic provides a baseline for comparison with the sensitivities of the actual model parameters. (**C**) Single-cell time-series trajectories plot of TAK1 (top row) and IKK (Bottom row) activation for synergistic cells (synergy score >1.25, red lines) and non-synergistic cells (synergy score <1, gray lines) in response to D2 LPS (left panel), D1 Pam (middle panel), or their combination (right panel). (**D**) Distribution curves of parameter functions—metrics derived from parameter combinations linked to specific biological functions—for synergistic cells (red curve, synergy score >1.25) and non-synergistic cells (gray curve, synergy score <1). Each parameter function is labeled on the x-axis, with its numeric values indicated. The y-axis shows the probability density, and the biological relevance of each parameter function is noted in the title. Associated *p*-values were calculated using one-sided Mann–Whitney *U*-tests. (**E**) Hill equation curves of TAK1-mediated IKK activation. For Hill coefficient = 2, if L1–L2 responses fall within the red-shaded region, synergy between L1 and L2 may occur.

To investigate the underlying regulatory mechanisms, we examined the LPS-Pam pair in detail (Fig. 4B; Appendix Figs. S8 and S9A), focusing on a representative synergistic condition, D2 (0.178 ng/mL) LPS with D1 (5.62 ng/mL) Pam. In the D2 LPS D1-Pam condition, out of 150 responsive cells, 28 were synergistic (synergy score >1.25) and 52 non-synergistic (synergy score <1) (Fig. EV4A).

To assess how biochemical parameters contribute to synergy, we first compared single-cell parameter distributions between synergistic cells and all responders (Appendix Fig. S9B). We then applied PAWN global sensitivity analysis (Khorashadi Zadeh et al, 2017; Pianosi and Wagener, 2015, 2018) to quantify how variation in each parameter influenced synergy score distributions (Fig. EV4B; see Methods). The most influential parameters were: (1) receptor abundance-related parameters (TLR4 synthesis rate k35, LPS-TLR4 degradation rate k44, and TLR1/2 synthesis rate k68), (2) TRAF6-mediated TAK1 activation (k52n2), and (3) the rate of NFκB-regulated IκBα transcription (k99).

To probe the mechanism underlying synergy, we compared responses of synergistic versus non-synergistic cells (Appendix Fig. S9C). In synergistic cells, TAK1 activation is reduced (Fig. EV4C), which may be attributed to low receptor abundance (k68 and k35), high ligand-receptor complex degradation (k75 and k44), and low TRAF6-dependent TAK1 activation (k52n2), collectively constraining TAK1 signaling (Fig. EV4D). Under these conditions, the ultrasensitive IKK activation (Hill = 2, required for TNF-induced NFκB oscillation, Appendix Fig. S10, Adelaja et al, 2021) driven by TAK1 enables synergy at the level of IKK at low ligand doses (Fig. EV4C,E). Propagation of this synergy to NFκB further depends on the downstream dynamical regime of the IKK-IκBα–NFκB module. Synergistic cells exhibited a non-oscillatory IκB-NFκB feedback regime (Appendix Fig. S9C), in which NFκB-induced IκBα transcription was tuned to be slower (k99) (Fig. EV4D), de-oscillating downstream dynamics. This downstream dynamical and parameter analysis shows that IKK-level synergy can be transmitted to NFκB only in a non-oscillatory regime, where IκBα negative feedback does not mask or reshape the upstream synergistic input. Together, these features suggest that ultrasensitive IKK activation permits low-dose ligand combinations to generate synergy (Fig. 4C).

Finally, to test whether such synergy enhances signaling fidelity, we simulated low-dose ligand combinations using models with IKK-activation synergy (Hill = 2) and without IKK-activation synergy (Hill = 1) (Appendix Fig. S11A,B). Removing ultrasensitivity (Hill = 1) abolished synergy (Fig. 4D). However, analysis of SRS by calculating Wasserstein distances between signaling codons (Guo et al, 2025) under these low-dose conditions (Appendix Fig. S11C,D) did not reveal improved separability with synergy (Fig. 4E,F). While ultrasensitivity enables synergistic peak responses, it does not guarantee synergy for total or other informative signaling codons (Appendix Fig. S11C,D) and therefore does not enhance SRS.

### Kinetic competition can lead to antagonism between high-dose ligands and enhance signaling specificity of ligand mixtures

Similarly, to identify cases of ligand-pair antagonism, we defined a single-cell antagonism score:

$${{{\rm{Antagonism\; Score}}}}=\frac{R\left(L1,L2\right)-R\left(0,0\right)}{\max \left(R\left(L1,0\right)-R\left(0,0\right),R\left(0,L2\right)-R\left(0,0\right)\right)}$$

**Figure 5:**
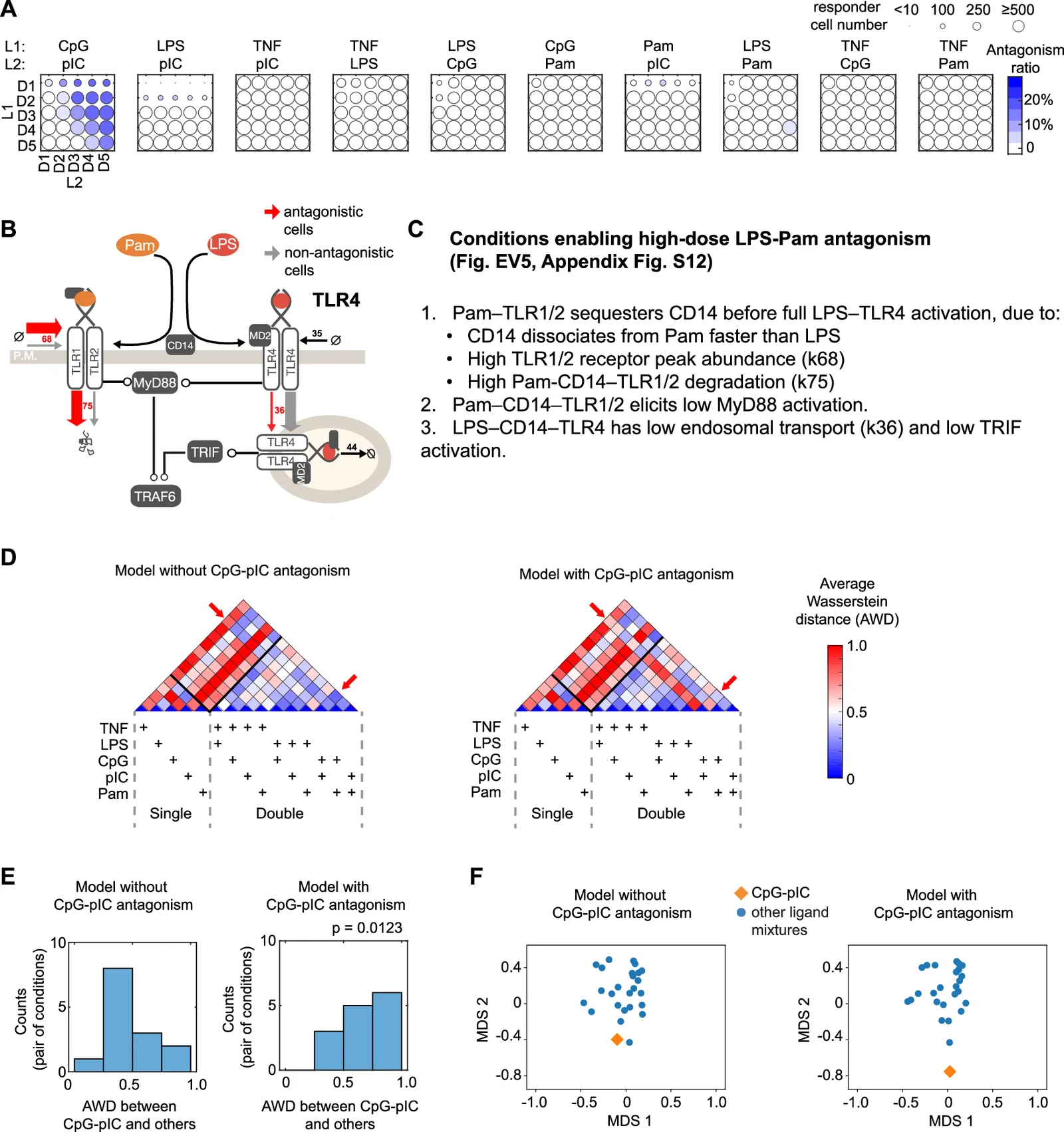
Evaluation of dose-dependent antagonism for ligand combinations and stimulus-response specificity. (**A**) Bubble chart showing the fraction of cells that show signaling antagonism (color) and the number of responder cells (bubble size) across ten ligand pairs. Each panel is labeled at the top with ligand 1 (L1) and ligand 2 (L2). Similar as Fig. 4A. (**B**) Schematic illustrating single-cell parameter differences between antagonistic and non-antagonistic cells in the LPS–Pam pathway. Red arrows mark the antagonistic parameter regime, and gray arrows mark the non-antagonistic regime. Figure elements are adapted from Adelaja et al, (2021) and Guo et al, 2025. (**C**) Summary of conditions enabling high-dose Pam-LPS antagonism, as delineated in Fig. EV5 and Appendix Fig. S12. (**D**) Heatmaps of the average Wasserstein distance between the distributions of six NFκB signaling codons for each of the 15 conditions (five single-ligand conditions and ten ligand pairs, detailed in Figs. EV1A and EV2A) for the original model that does not contain CpG-pIC antagonism and the updated model that does contain CpG-pIC antagonism (indicated in the title of each subpanel). Conditions are identified on the diagonal axis. Color intensity indicates average Wasserstein distance in accordance with the color bar. (**E**) Histogram of the number of single and double-ligand conditions (out of 14 total) whose average Wasserstein distance (AWD) to the CpG-pIC condition falls within the indicated bin for each of the alternative models (as labeled in the title of each subpanel). The *p*-value shown in the upper right of the right panel was calculated using a paired Wilcoxon signed-rank test comparing the matched condition-pair distances between the two models. (**F**) MDS visualization of ligand-combination similarity with and without CpG-pIC competition. Multidimensional scaling (MDS) was applied to the pairwise AWD matrix to visualize similarities among simulated ligand-combination conditions. Each blue point represents one ligand-mixture condition, and the orange diamond indicates the CpG-pIC condition. The left panel shows the MDS embedding when CpG-pIC competition is excluded, whereas the right panel shows the embedding when CpG-pIC competition is included. Source data are available online for this figure.

Cells with an antagonism score <1 exhibit a combined response smaller than at least one of the single-ligand responses. To exclude spurious cases arising from numerical noise, we classified cells as antagonistic only when their score was <0.9 (Fig. EV3; see Methods). Computing the fraction of antagonistic cells across all 360 conditions (Fig. EV3, Step 8) revealed two ligand pairs that produced antagonism among high dosage: CpG-pIC and LPS-Pam (Fig. 5A).

**Figure EV5:**
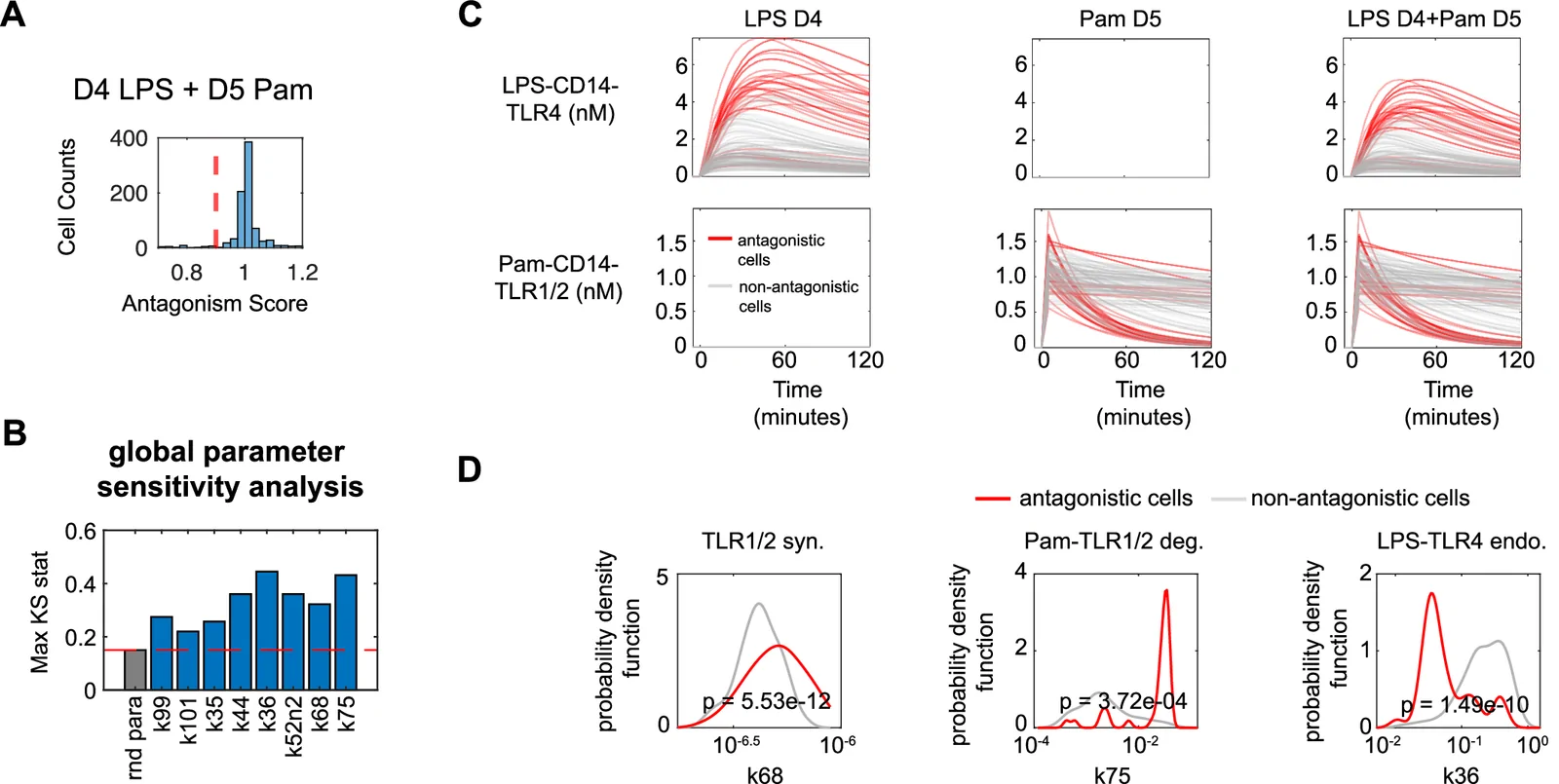
Conditions for single-cell antagonism under LPS D4 + Pam D5 co-stimulation. (**A**) Histogram of antagonism scores for responsive cells under combined stimulus conditions of Dose 4 (D4) LPS and Dose 5 (D5) Pam. (**B**) Bar plot indicating the feature importance score of each parameter, as evaluated via PAWN parameter analysis for condition of D4 LPS + D5 Pam. The KS stat is the Kolmogorov–Smirnov distance between the unconditional output distribution and the output distribution conditioned on a given parameter, with a larger KS value indicating a stronger influence of that parameter on the model output. The parameter index is the same as in Fig. 4B. “rnd para” denotes a randomly assigned variable included in the model as a negative control, and its corresponding KS statistic provides a baseline for comparison with the sensitivities of the actual model parameters. (**C**) Single-cell time-series trajectories plot of LPS-CD14-TLR4 (top row) and Pam-CD14-TLR1/2 (Bottom row) activation for antagonistic cells (antagonism score <0.9, red lines) and non-antagonistic cells (antagonism score >1.1, gray lines) in response to D4 LPS (left panel), D5 Pam (middle panel), or their combination (right panel). (**D**) Distribution curves of parameters for antagonistic cells (red curve, antagonism score <0.9) and non- antagonistic cells (gray curve, antagonism score >1.1). Each parameter function is labeled on the x-axis, with its numeric values indicated. The y-axis shows the probability density, and the biological relevance of each parameter function is noted in the title. Associated *p* values were calculated using one-sided Mann–Whitney *U*-tests.

To characterize antagonism in detail, we focused on the LPS-Pam pair and examined a representative antagonistic condition consisting of D4 LPS (1.78 ng/mL) combined with D5 Pam (562 ng/mL) (Appendix Fig. S12A). Among 863 responsive cells, 36 displayed antagonism (score <0.9), while 569 were non-antagonistic (score >1.0), including 61 cells with strongly integrative responses (score >1.1) (Fig. EV5A).

We next compared parameter distributions between antagonistic cells and the overall responsive population (Appendix Fig. S12B) and applied PAWN global sensitivity analysis (Khorashadi Zadeh et al, 2017; Pianosi and Wagener, 2015, 2018) to determine which kinetic parameters most strongly shaped antagonism scores (See Methods, Fig. EV5B). The most influential parameters included: (1) receptor abundance—related parameters—LPS-TLR4 degradation (k44), TLR1/2 synthesis (k68), and Pam-TLR1/2 complex degradation (k75); (2) LPS-TLR4 endosomal transport (k36); and (3) TRAF6-dependent TAK1 activation (k52n2).

To identify the underlying mechanism, we compared signaling trajectories between antagonistic and non-antagonistic cells (Appendix Fig. S12C). In antagonistic cells, early Pam signaling disrupts full LPS pathway activation by sequestering CD14. Because Pam dissociates from CD14 more rapidly than LPS, Pam reaches its signaling peak earlier (Fig. EV5C), allowing Pam-TLR2 complexes to occupy CD14 before LPS-TLR4 engagement can fully mature (Appendix Fig. S13A,B). This sequestration is further reinforced by increased TLR1/2 receptor abundance (k68) and accelerated degradation of Pam-CD14-TLR1/2 complexes (k75) (Fig. EV5D), collectively diverting CD14 away from productive LPS signaling. Moreover, TLR-CD14-Pam complexes generate weaker MyD88 activation than LPS-TLR4 (Appendix Fig. S12C), so preferential routing of CD14 to the Pam pathway dampens downstream NFκB activation. Concurrently, LPS-CD14-TLR4 complexes exhibit limited TRIF activation due to reduced endosomal transport (k36) (Fig. EV5D), preventing robust late-phase signaling. Together, these features indicate that early Pam-driven CD14 sequestration, combined with impaired LPS-TLR4 TRIF signaling, coordinately produces high-dose Pam-LPS antagonism (Fig. 5B,C).

Collectively, these analyses show that CD14, a shared co-receptor required for both the TLR1/2-Pam and TLR4LPS pathways, acts as a limiting resource whose competitive allocation constrains dual-ligand responses. Strong CD14 demand by LPS-TLR4 together with the early, high Pam-TLR1/2 activation intensifies this competition, resulting in antagonism under combined stimulation (Fig. 5C).

Finally, to evaluate how antagonism influences signaling fidelity, we compared SRS in the original model that does not contain CpG-pIC antagonism and the updated model that does contain CpG-pIC antagonism. Wasserstein distances between single-ligand and paired-ligand signaling codons showed significantly greater separability in the updated model with CpG-pIC antagonism (Fig. 5D,E). Extending this analysis across all combinatorial ligand conditions similarly demonstrated increased SRS in the updated model with CpG-pIC antagonism, indicating that antagonism enhances stimulus specificity in complex ligand environments (Appendix Fig. S14). By visualizing the pairwise Wasserstein distance matrix in a two-dimensional MDS space, we found that excluding CpG-pIC competition shifted the CpG-pIC condition closer to the main cluster of ligand mixtures, whereas including CpG-pIC competition separated CpG-pIC from other combinatorial conditions (Fig. 5F). Together, these results suggest that CpG-pIC antagonism generates a more distinguishable combinatorial response profile relative to other ligand mixtures.

## Discussion

Here, we report macrophage NFκB signaling responses to ligand mixtures and identify mechanisms of ligand-combination synergy and antagonism that shape stimulus-response specificity (SRS) to immune threats. We developed a workflow to generate large-scale simulations of macrophage NFκB responses to all possible ligand combinations, validated by experimental datasets (Figs. 1–3), and extended this framework to predict combinatorial responses across full dose ranges to identify synergistic and antagonistic regimes and their underlying mechanisms (Figs. 4, 5). Our results demonstrate that macrophages maintain partial NFκB signaling SRS in complex ligand-mixture contexts. We report that such specificity is in part mediated by signaling antagonism that results from biochemical bottlenecks, such as limited capacity for endosomal transport or a limited supply of TLR cofactor CD14.

Although combinatorial stimulus conditions are the physiological norm in nature, experimental studies of signaling dynamics have not been able to provide a comprehensive survey, given the exponential explosion of combinatorial stimulus conditions when multiple ligands and multiple doses are considered. Here, we developed a computational, systems-level strategy that leverages a mechanistic mathematical model to systematically map macrophage responses. The five representative single ligands spanning bacterial, viral, and cytokine stimuli (Guo et al, 2025) applied at six doses resulted in 2.88 million single-cell simulations. Importantly, the model simulations provided access not only to the effector output of the network, NFκB dynamics, but also to the trajectories of all molecular species, including 12 key signaling nodes such as IKK, TAK1, and MyD88. This enabled us not only to determine stimulus-response specificity of NFκB dynamics and identify cases of synergy and antagonism but to investigate the underlying molecular network causes of these phenomena and develop a mechanistic understanding of their context-dependence.

The methodology employed in this work exemplifies the power of combining prior knowledge of signaling networks with appropriate statistical methods. Previous work identified single-cell parameter distributions that generate reliable predictions beyond the training range (Guo et al, 2025), which were validated by independent experiments across extended doses, mutant genotypes, and upstream kinase (Adelaja et al, 2021; Cheong et al, 2011; Cruz et al, 2021). This approach leverages a nonlinear mixed-effects (NLME) statistical framework (Lindstrom and Bates, 1990; Lavielle and Mbogning, 2014; Delyon et al, 1999) that combines parameter distribution estimation with a knowledge-based ordinary differential equation (ODE) dynamic model (Adelaja et al, 2021). In the present study, we demonstrate that a knowledge-based mechanistic model (Guo et al, 2025), together with a statistical matching approach (D’Orazio et al, 2006), enables predictions that extend into combinatorial stimulus space beyond the training range. Specifically, we treated shared core-module parameters as common variables and applied nearest-neighbor hot-deck imputation (Andridge and Little, 2010), effectively using the core module as an anchor to align distinct receptor-specific parameter spaces. Similar methodologies have been applied to correct batch effects or integrate single-cell RNAseq datasets using anchor cell types (Stuart et al, 2019). However, applying this approach to mechanistic model parameterizations is a means to grow the predictive power of mechanistic network simulations while preserving measured single-cell heterogeneity profiles. Importantly, we validated the approach for key ligand combinations in this work (Figs. 1, 2 and EV1 and EV2). As such, the approach provides a pathway to developing virtual cell models that function as knowledge bases for mechanistic models while recapitulating the heterogeneity of single-cell dynamic responses to perturbations.

By iteratively comparing mechanistic model predictions with experimental validation, we identified a key signaling integration feature: resource-limited antagonism. For example, we identified a discrepancy between model predictions and experimental observations under CpG-pIC stimulation and resolved it by incorporating Michaelis–Menten kinetics, without introducing free parameters, to model endosomal transport competition between CpG and pIC (Fig. 2). We also identified CD14 competition between LPS and Pam as contributing to NFκB signaling antagonism (Fig. 5), aligning with previous experiments reporting it as a non-integrative combination (Kellogg et al, 2017). Although antagonism between CpG and pIC could, in principle, arise from alternative mechanisms—such as pathway crosstalk or parallel-pathway interference—the experimental data indicate that the antagonistic effect must occur within the first 30 min. That renders gene expression–mediated feedback via IκBα (Hoffmann et al, 2002) or A20 (Werner et al, 2005) unlikely. Further, the shared IKK activation pathway depends primarily on phosphorylation and K63-linked ubiquitination (Wertz and Dixit, 2010; Chen, 2005), which combinatorial inputs may saturate but not produce antagonism.

For the analysis of the enormous dataset, we defined a new computational approach for SRS calculation in the context of ligand mixtures. This approach is only possible because of the large, comprehensive dataset we generated, which includes all ligand-mixture conditions. We employed machine learning classifiers (Adelaja et al, 2021) and Wasserstein distance to quantify condition discrimination and similarity (Guo et al, 2025) (Fig. 3). Early quantification of specificity and signaling fidelity began with definitions based on signal integral ratios capturing signaling crosstalk (Komarova et al, 2005). Subsequent application of information theory to cellular signaling systems advanced the field by enabling quantitative measurement of specificity (Cheong et al, 2011), later extended to dynamic vectors (Selimkhanov et al, 2014). Mechanistic models were then employed to investigate SRS using information theory (Suderman et al, 2017). More recently, machine learning approaches have demonstrated strong performance in quantifying SRS (Tang and Hoffmann, 2022; Rahman et al, 2024; Singh et al, 2024; Luecke et al, 2024). In both information-theoretic and machine-learning approaches, each condition (ligand, doses, or combinatorial) is typically treated as its own category or class. This approach may fail with an increasing number of conditions. For example, classifier performance metrics deteriorate as the number of conditions increases (Adelaja et al, 2021). In this work, we introduced binary present-or-not classifiers to distinguish conditions with or without a specific ligand across all ligand-mixture conditions (Fig. 3D,E). This formulation models a fundamental function of macrophages: determining whether specific immune threats are present in a complex environment (Sheu and Hoffmann, 2022; Lavin et al, 2015). By comparing the results of mutual information (MI) and corresponding machine learning (ML) classifier performance (Appendix Fig. S15; Fig. 3D), we found that MI metrics are highly sensitive for evaluating perfectly or near-perfectly distinguishable conditions, while ML metrics can assess differences in distinguishability among imperfectly classified cases. The simplified binary classifier-based metric provides a feasible approach for quantifying essential macrophage decision-making in complex environments, which can theoretically encompass an infinite number of conditions. The concept of stimulus-response specificity and the associated computational paradigm for SRS in ligand mixtures may extend beyond innate immune cells to other cell types, as all cells must be able to detect differences in environmental cues and respond appropriately.

Given cell heterogeneity, signaling pathway synergy or antagonism manifests in some but not all cells. We found that a population average measure was too insensitive to detect such functionally relevant interactions between pathways. While experimentally measuring how the same cell responds to different ligands and their corresponding pairwise combinations is infeasible, we could do so in our computational simulations. This capability allowed us to define synergistic and antagonistic NFκB signaling in single cells, and thus identify the mechanisms and contextual conditions that drive synergy or antagonism. For example, we identified synergy regimes in ~5–10% of single cells arising from IKK ultrasensitivity, but only when specific contextual network conditions were met (Fig. 4). While the current manuscript focuses on the emergent properties of SRS and ligand identification, the supporting data files provide a complete mechanistic description of their context-dependence. This mechanistic detail documents the unprecedented mechanistic knowledge that we have now attained in how inflammatory stimuli activate NFκB.

Cells interpret not only the combination of signals but also their timing and duration to mount appropriate transcriptional responses (Kumar et al, 2004; Son et al, 2023). For example, acute inflammation integrates pathogen-derived cues with pro- and anti-inflammatory signals over a timeframe of hours to days (Kumar et al, 2004), to coordinate the pathogen removal and tissue repairing process. Investigating sequential stimulus combinations in our model is therefore crucial for understanding how cells process complex physiological inputs. Simulations that account for longer timescales may require additional feedback mechanisms, as described in some of our previous studies for NFκB (Werner et al, 2005, 2008). With the inclusion of gene regulatory networks, additional sources of cellular heterogeneity may be considered, but advances in measuring single-cell gene expression trajectories (scGETs) (Sheu et al, 2024) may begin to provide the requisite datasets that allow for parameterizing an expanded innate immune response network model and the further development of the computational workflow described here.

Furthermore, innate immune responses do not solely rely on NFκB but also involve the critical functions of AP1, p38, and the IRF3-ISGF3 axis. The additional pathways are likely activated in a coordinated manner and provide additional information (Luecke et al, 2021). This is exemplified by the studies demonstrating synergistic effects between CpG and pIC in inhibiting tumor growth and promoting cytokine production (Huang et al, 2020), such as IFNβ and TNFα, whose expression is also regulated by the IRF and MAPK signaling pathways (Sheu et al, 2023; Luecke et al, 2021). Therefore, the inclusion of parallel pathways of AP1 and MAPK, as well as the type I interferon network (Cheng et al, 2015; Davies et al, 2020; Peterson et al, 2022; Paek et al, 2016; Luecke et al, 2024; Hanson and Batchelor, 2022) are next steps expanding the mathematical models presented here.

Our work advances the field by providing a mechanistic understanding of stimulus–response specificity under physiologically relevant conditions of ligand mixtures. We describe a computational workflow and analysis strategies that enable the inclusion of additional regulatory modules in the quest of developing whole virtual cell simulations based on mechanistic knowledge that recapitulate the precision of heterogeneous single-cell responses. The present work not only provides motivation for such studies, but also offers a blueprint for analytical strategies and highlights the role of mathematical models in extending the capabilities of experimental studies.

## Methods

**Table:** Reagents and tools table

Reagent/resource	Reference or source	Identifier or catalog number
**Experimental models**		
Cell Line: RelA-mVenus hMPs	Singh, Sen et al, 2024	hMPs
Strains: RelA^mVenus/mVenus^ (C57BL/6)	Adelaja et al, 2021	JAX stock 38987
**Recombinant DNA**		
**Antibodies**		
**Oligonucleotides and other sequence-based reagents**		
**Chemicals, Enzymes and other reagents**		
lipopolysaccharide (LPS)	Sigma- Aldrich, B5:055	L2880
Murine TNF	Roche	11271156001
Pam3CSK4	InvivoGen	tlrl-pms
polyinosine-polycytidylic acid (Poly(I:C))	InvivoGen	tlrl-picw
Synthetic CpG ODN 1668	InvivoGen	tlrl-1668
Hoechst 33342 dye	Thermo Fisher	62249
**Software**		
MACKtrack - Image Analysis	Adelaja et al, 2021	https://github.com/brookstaylorjr/MACKtrack
Single-cell NFκB models and parameterization	Guo et al, 2025	https://github.com/Xiaolu-Guo/Virtual_single_cell_NFkB
Combinatorial ligand NFκB dynamic simulations	This study	https://github.com/Xiaolu-Guo/Combinatorial_ligand_NFkB
NFκB trajectory feature calculation	Guo et al, 2025	https://github.com/Xiaolu-Guo/Virtual_single_cell_NFkB
Random Forest Classifier	Breiman, 2001	https://scikit-learn.org/stable/
LSTM classifiers	Hochreiter and Schmidhuber, 1997	http://arxiv.org/abs/1605.08695
Wasserstein-2 distances	Peyré and Cuturi, 2019	https://www.mathworks.com/matlabcentral/fileexchange/75228-wasserstein-distance/files/ws_distance.m https://doi.org/10.1561/2200000073
Single-cell synergy and antagonism score	This study	https://github.com/Xiaolu-Guo/Combinatorial_ligand_NFkB
PAWN global sensitivity analysis	Pianosi and Wagener, 2015	https://www.mathworks.com/matlabcentral/fileexchange/69436-pawn-global-sensitivity-analysis-algorithm
MATLAB ODE simulation	The MathWorks Inc.	https://www.mathworks.com/
**Other**		

### Simulation of heterogenous NFκB dynamical responses

A well-established ODE model of NFκB signaling (Adelaja et al, 2021; Guo et al, 2025) was used to investigate NFκB dynamics in response to different stimuli. Detailed model structure is given in Dataset EV2.

We performed simulations using a previously established mathematical model of NFκB signaling, represented by a 52-dimensional system of ordinary differential equations (ODEs) (Adelaja et al, 2021). This model includes five receptor modules (TNF, LPS, CpG, pIC, and Pam) interacting with a shared core NFκB-IKK-IκBα signaling module (Appendix Fig. S1). The representative-cell mechanistic model was calibrated using a representative cell from experimental NFκB trajectory data collected from primary bone marrow-derived macrophages obtained from mVenus-RelA reporter mice.

The ODE model was then fitted to the population of single-cell trajectories to recapitulate the cell-to-cell heterogeneity in the experimental data (Guo et al, 2025). This is achieved by solving the nonlinear mixed-effects model (NLME) through stochastic approximation of the expectation maximization algorithm (SAEM) (Delyon et al, 1999; Lavielle and Mbogning, 2014; Llamosi et al, 2016; Dharmarajan et al, 2019). Seventeen parameters were estimated, with 6–7 parameters for each ligand. Within the core module, the estimated parameters included the rates governing TAK1 activation (k52 and k65), the time delays of IκBα transcription regulated by NFκB (k99 and k101), and the overall NFκB abundance (tot NFκB). Within the receptor module, receptor synthesis rates (k54 for TNF, k68 for Pam, k85 for CpG, k35 for LPS, k77 for pIC), degradation rates of the receptor-ligand complexes (k56, k61, k64 for TNF; k75 for Pam; k93 for CpG; k44 for LPS; k83 for pIC), and endosomal uptake rates (k87 for CpG; k36 and k40 for LPS; k79 for pIC) were fitted. All remaining parameters were fixed at literature-suggested values (Adelaja et al, 2021). The single-cell parameters inferred from experimental individual-cell trajectories then served as an empirical distribution for generating the new dataset (see Dataset EV1). To better reproduce the pIC-stimulated NFκB dynamics in HoxB4-mediated transduction-derived macrophages (see Experimental data generation), the pIC signaling complex degradation rate (k83) was increased fivefold.

We applied bootstrapping sampling from the empirical distribution:

7 $$f\left(\psi \right)={\sum}_{m=1}^{M}\frac{1}{M}\delta \left(\psi -{\hat{\psi }}^{\left(m\right)}\right)$$

where M represents the total number of cells across all dosage conditions for that specific ligand. The sampled heterogeneous single-cell parameters were applied to simulate the heterogeneous single-cell NFκB signaling trajectories using the 52-dimensional ODE (see Guo et al, 2025 supplementary for full sets of equations). Simulation results showed that the bootstrapping approach replicates the experimental NFκB dynamic feature distributions.

Using the sampled single-cell parameters, the model was numerically solved using MATLAB’s ode15s solver in two distinct simulation phases. The first phase established the steady-state conditions, followed by the second phase applied single-ligand or combinatorial-ligand stimulations. A total of five ligand stimulations at high dose and their combination were applied: TNF at 10 ng/mL; LPS at 10 ng/mL; CpG at 100 nM; pIC at 100 µg/mL; and Pam at 100 ng/mL. Trajectories are simulated for 8 h after stimulation and saved every 5 min. All simulation results were visualized using MATLAB.

### Predicting heterogeneous single-cell responses to combinatorial-ligand stimulation

For predicting responses to combinatorial ligand stimuli, receptor module parameter sets were matched based on core module parameter sets using equations [1–2]. The workflow of combinatorial ligand stimulation was adapted from Guo et al, 2025. For each condition, 1000 cells were simulated using MATLAB’s ode15s solver. Simulations included an initial phase to establish a steady state, followed by stimulation. The resulting trajectories were visualized and analyzed in MATLAB.

We employed the bootstrap-sampling and core-module-matching strategy previously described by Guo et al, 2025 to assemble heterogeneous virtual single-cell NFκB network parameters from the inferred single-cell dataset. A more detailed description of this procedure follows below. These diverse parameter sets were then fed into the NFκB ODE model to simulate single-cell NFκB signaling trajectories in response to various ligand combinations.

Because the original single-cell parameter estimates included only one receptor module plus the shared core module, parameters for additional receptors had to be imputed to reconstruct each cell’s full network (see Fig. S5A in (Guo et al, 2025)). To build 600 distinct virtual cells containing two receptor modules and the core, we first performed bootstrap resampling on the inferred single-cell dataset for the “first” ligand: we drew 300 parameter sets (with replacement), each providing that ligand’s receptor parameters together with the core module parameters. Next, for each of these 300 resampled cells, we selected the second receptor’s parameters by finding—in the pool of inferred single-cell data—the cell whose core module profile most closely matched the resampled core. That yielded 300 fully specified, heterogeneous single-cell networks. We then inverted the process—bootstrapping the second ligand’s parameters first and matching the first ligand’s receptor module—to produce an additional 300 networks, bringing the total to 600.

For example, to model co-stimulation by TNF and LPS, we began by sampling 300 parameter sets from TNF-stimulated single cells (TNF receptor plus core). For each sampled core, we identified the LPS-stimulated cell whose core profile differed least, and then assigned that cell’s LPS receptor parameters. This generated 300 TNF-first virtual cells. Performing the reverse (sampling LPS first, then matching TNF) produced another 300, together forming 600 heterogeneous virtual cells capable of predicting responses to TNF  +  LPS stimuli.

More generally, to generate N heterogeneous single-cell NFκB networks for predicting responses to n-ligand stimuli, we first sampled N/n parameter sets corresponding to the 1st receptor module and core module from the experimental data inferred parameter sets. These defined the core and one receptor module parameters for each virtual single cell. To infer parameters for the remaining n−1 receptor modules, we assigned values from 2nd ligand stimulated single cells with same—or the most similar—core module parameters (Eqs. 1–2 in main text). This process was repeated iteratively, treating each receptor module as the initial sampling module in turn, until N virtual single-cell NFκB signaling networks were fully reconstructed.

The sampled parameters were then applied to simulate single-cell NFκB signaling trajectories using a 52-dimensional ODE model (see Dataset EV1). For each condition, between 999 and 1,000 cells were simulated (*N* = 999 for *n* = 3; *N* = 1000 for *n* = 1, 2, 4, 5) using Matlab’s ode15s solver. Simulations included an initial phase to establish steady-state conditions, followed by stimulation. The resulting trajectories were visualized in MATLAB.

### Experimental data generation

#### Macrophage cell culture and stimulation

RelA-mVenus mouse strain (Adelaja et al, 2021) were used to generate myeloid precursor cells via HoxB4-mediated transduction (hMP) (Ruedl et al, 2008). In the RelA-mVenus mouse model, the NFκB subunit RelA is tagged with the fluorescent protein mVenus, allowing live-cell monitoring of NFκB nuclear translocation. To obtain hMP-derived macrophages (hMPDMs), hMPs were differentiated in L929-conditioned medium following the standard bone marrow–derived macrophage protocol (Adelaja et al, 2021). On days 6 or 7 of differentiation, hMPDMs were re-plated into eight-well ibidi SlideTek chambers at 12,000–18,000 cells per well. Imaging was performed when cultures reached optimal density on days 10–12. Prior to stimulation, cells were maintained in their original medium. Stimuli—applied without medium change—included LPS (10 ng/mL, TLR4 agonist; Sigma-Aldrich), pIC (Poly(I:C), 100 µg/mL, TLR3 agonist; InvivoGen), CpG B ODN (100 nM, TLR9 agonist; InvivoGen), Pam (Pam3CSK4, 100 ng/mL, TLR2 agonist; InvivoGen), TNF (10 ng/mL; Roche), and their combinations (Fig. EV1). Concentrations were optimized to elicit maximal NFκB activation.

#### Live-cell Imaging

Two hours before imaging, hMPDMs were incubated with Hoechst 33342 (5 ng/mL) for nuclear labeling. Time-lapse imaging was conducted on a Zeiss AxioObserver platform with live-cell incubation within ibidi chambers, equipped with a Sutter Lambda XL light source, acquiring both DIC and epifluorescence every 5 min. The first three frames (pre-stimulation) established each cell’s NFκB baseline. Fifteen minutes into imaging, the appropriate stimulus was introduced directly into each well. Images were recorded continuously for 8 h using a Hamamatsu Orca-Flash 2.0 CCD camera.

#### Image processing

Acquired time-lapse microscopy images were exported to MATLAB R2018a for single-cell segmentation and tracking using the MACKtrack pipeline (https://github.com/brookstaylorjr/MACKtrack) (Adelaja et al, 2021). DIC images guided cell identification, nuclear outlines were refined by Hoechst fluorescence, and segmented objects were linked across frames. Nuclear and cytoplasmic regions were defined to quantify mVenus-NFκB fluorescence per cell. Background normalization and baseline subtraction were performed by averaging the first three pre-stimulation frames and subtracting this value from each trajectory. Time = 0 was set at the third frame, and the subsequent 97 time points (8 h) were analyzed. Any mitotic cells or those that exited the field of view were excluded from further analysis.

After acquiring fluorescent images of live cells and processing them into single-cell nuclear NFκB activity time trajectories (in arbitrary units), we rescale these measurements into standard units (Guo et al, 2025) for further analysis and quantification. We then compare the rescaled single-cell NFκB trajectories with the model’s predictions.

### Responder fraction

We decomposed NFκB trajectories into six NFκB signaling codons—Speed, Peak, Duration, Total, EvL, and Osc—that encode stimulus information (Adelaja et al, 2021). The quantification was described (Guo et al, 2025) (see SI Appendix for details). Responder fractions were determined by imposing specified thresholds on Peak and Total.

Simulation responder ratio is calculated via peak value or integral higher than a specific threshold, implemented in MATLAB. Experimental data are first rescaled to a similar numerical range under standard units (Singh et al, 2024; Luecke et al, 2024; Guo et al, 2025), then calculated the responder ratio. For the model simulation data, the integral thresholds are 0.4 (µM·h), 0.5 (µM·h), and 0.6 (µM·h). The peak thresholds are 0.12 (µM), 0.14 (µM), and 0.16 (µM). For the experimental data, the integral thresholds are 0.2 (A.U.·h), 0.3 (A.U.·h), and 0.4 (A.U.·h). The peak thresholds are 0.14 (A.U.), 0.18 (A.U.), and 0.22 (A.U.). Thresholds were manually selected so that the medium threshold yield, on average, 50–60% responder cells under single-ligand conditions, while the responder ratio remains unsaturated under three-ligand stimulation.

### Signaling codon decomposition

Six signaling codons are dynamic features capturing the stimulus-specific information encoded in macrophage NFκB trajectories (Adelaja et al, 2021). The calculation is the same as (Guo et al, 2025), and detailed in the codes (https://github.com/Xiaolu-Guo/Combinatorial_ligand_NFkB). Briefly: “Speed” captures how rapidly NFκB activation occurs. “Peak” records the maximum signaling level. “Duration” measures how long NFκB activity remains above the activation threshold. “Total” corresponds to the area under the response curve. “Early vs. Late” (“EvL”) indicates whether NFκB activity is weighted toward the early or late phase of the response. “Oscillatory Power” (“Osc”) reflects the strength of NFκB oscillations driven by IκBα feedback. Below are the formal definitions of each codon, based on the simulation dataset and rescaled experimental dataset.

Let cell (m) NFκB signaling trajectory be

$${X}^{(m)}=({x}_{1}^{(m)},{x}_{2}^{(m)},{x}_{3}^{(m)},\ldots ,\,{x}_{N}^{(m)})$$

observed at time $\left(i-1\right)\cdot \Delta t$, for $i=1,2,3,\ldots ,\,N$.

1. Speed: for the trajectory ${X}^{(m)}$, we define the local maxima peak sets ${\Lambda }_{{peak}}$ as
  $${\Lambda }_{{peak}}=\{{x}_{i}^{\left(m\right)}:{x}_{i}^{\left(m\right)} > \, {x}_{i-1}^{m}{{\& }} \, {x}_{i}^{\left(m\right)}\ge {x}_{i+1}^{m} \, {{\& }} \, {x}_{i}^{\left(m\right)} > 0\}$$
  We define the index of the first peak time point as:
  $${i}_{{peak}}^{(m)}=\min (i\in {\Lambda }_{{peak}}{|i} > 3)$$
  Speed is defined by three components:
  $${{\rm{peak}}}1{{\_time}}\left({X}^{\left(m\right)}\right)=\left({i}_{{peak}}^{(m)}-1\right)\cdot \Delta t$$
  $$\max \_{{\rm{pk}}}1{{\_{{\rm{speed}}}}}\left({X}^{\left(m\right)}\right)={\max }_{j < {i}_{{peak}}^{(m)}}{x^{\prime} }_{j}^{\left(m\right)}$$
  and
  $${{\rm{Derivative}}}\_{{\rm{initial}}}\left({X}^{\left(m\right)}\right)={x^{\prime} }_{2}^{\left(m\right)}$$
  where ${x{\prime} }_{j}^{\left(m\right)}$ are the numerical derivatives (with respect to time) of $({x}_{1}^{(m)},{x}_{2}^{(m)},{x}_{3}^{(m)},\ldots ,\,{x}_{N}^{(m)})$ at time frame j.
  Taken all together, Speed is defined by:
  $${Speed}\left({X}^{\left(m\right)}\right)= 	 \frac{1}{3}\left(-z\left({{\rm{peak}}}1\_{{\rm{time}}}({X}^{(m)})\right)\right. \\ 	 \left. +z\left(\max {{\_{{\rm{pk}}}}}1\_{{\rm{speed}}}({X}^{(m)})\right) \right. \\ 	 \left. +z\left({{\rm{Derivative}}}\_{{\rm{initial}}}({X}^{(m)})\right)\right)$$
  where $z\left(\cdot \right)$ are the z-score across all cell among all conditions for corresponding component.
2. Peak is defined by two components; first component is the maximal value of each trajectory:
  $$\max \_{{\rm{val}}}\left({X}^{\left(m\right)}\right)={\max }_{{{\rm{i}}}}({x}_{i}^{\left(m\right)})$$
  second component is the peak values of the first peak:
  $${{\rm{peak}}}1{{\_{{\rm{amp}}}}}\left({X}^{\left(m\right)}\right)={x}_{{i}_{{peak}1}^{(m)}}^{m}$$
  and Peak is defined by
  $${Peak}({X}^{(m)})=\frac{1}{2}(z(\max \_{{\rm{val}}}({X}^{(m)}))+z({{\rm{peak}}}1\_{{\rm{amp}}}({X}^{(m)})))$$
3. Duration:
  $${Speed}({X}^{(m)})=\left|\left\{i:{x}_{i}^{\left(m\right)} > {threshold}\right\}\right|\cdot \Delta t$$
4. Total:
  $${Total}\left({X}^{\left(m\right)}\right)={\sum }_{k=1}^{N}\frac{\left({x}_{k}^{\left(m\right)}+{x}_{k+1}^{\left(m\right)}\right)\Delta t}{2}$$
5. EvL:
  $${EarlyVsLate}({X}^{(m)})={{{\rm{argmin}}}}_{{i}}|{\sum }_{k=1}^{i}\frac{({x}_{k}^{(m)}+{x}_{k+1}^{(m)})\Delta t}{2}-\frac{1}{2}{\max }_{l}{\sum }_{k=1}^{l}\frac{({x}_{k}^{(m)}+{x}_{k+1}^{(m)})\Delta t}{2}|\cdot \Delta t$$
6. Osc: for the trajectory $\{{x}_{i}^{\left(m\right)}\}$, we calculate discrete Fourier transform $\{\hat{{x}^{(m)}}\left({f}_{k}\right)\}$ and the power spectral density (PSD) $\left\{\hat{{x}^{\left(m\right)}}{\left({f}_{k}\right)}^{2}\right\}$. The Osc signaling codon is the summation of PSD over the frequency range between 0.33 and 1 h^−1^.

$${Osc}({X}^{\left(m\right)})={\sum}_{k:0.33\le {f}_{k}\le 1}\hat{{x}^{\left(m\right)}}{\left({f}_{k}\right)}^{2}$$

For each NFκB trajectory, the above six signaling codons are calculated in MATLAB.

### Model refinement for CpG and pIC

Given that both CpG and pIC signaling activation requires endosomal transport, they may compete for cellular resources for endosomal import. For example, both CpG and pIC require Scavenger Receptors (Murshid et al, 2016). Scavenger Receptors promote transport from the membrane to the endosome, while they themselves may be recycled (Mori et al, 1994; Stephen et al, 2010). These processes are similar to enzyme dynamics, in which the enzyme promotes the conversion of substrates into products and is recycled after the reaction is completed. In the previous model (Fig. 1), CpG and pIC endosomal transport were assumed to be independent, and the corresponding reactions are:

$${{\rm{E}}}+{{\rm{CpG}}}\begin{array}{c}{{{\rm{k}}}}_{+1,{{\rm{CpG}}}}\\ \rightleftharpoons \\ {{{\rm{k}}}}_{-1,{{\rm{CpG}}}}\end{array}{{\rm{ECpG}}}{\to }^{{k}_{{cat},{CpG}}}E+{{CpG}}_{{en}}$$

$${{\rm{E}}}+{{\rm{pIC}}}\begin{array}{c}{k}_{+1,{pIC}}\\ \rightleftharpoons \\ {k}_{-1,{pIC}}\end{array}{{\rm{EpIC}}}{\to }^{{k}_{{cat},{pIC}}}E+{{pIC}}_{{en}}$$

where ${{\rm{E}}}$ are the resources for the endosomal transport, such as Scavenger Receptor, which is recycled after the reaction. Based on the Michaelis–Menten kinetics (Michaelis et al, 2011), the CpG endosomal transport rate can be approximated by:

$$v({CpG})=\frac{\frac{{k}_{{{\rm{cat}}},{{\rm{CpG}}}}}{{K}_{m,{CpG}}}* E0* {{\rm{CpG}}}}{1+\frac{{CpG}}{{K}_{m,{CpG}}}}=\frac{{v}_{max ,{pIC}}* {{\rm{CpG}}}}{1+\frac{{CpG}}{{K}_{m,{CpG}}}}$$

$$v({pIC})=\frac{\frac{{k}_{{{\rm{cat}}},{pIC}}}{{K}_{m,{pIC}}}* E0* {pIC}}{1+\frac{{pIC}}{{K}_{m,{pIC}}}}=\frac{{v}_{max ,{pIC}}* {pIC}}{1+\frac{{pIC}}{{K}_{m,{pIC}}}}$$

where ${v}_{max }=\frac{{k}_{{{\rm{cat}}}}}{{K}_{m}}* E0$, E0 is the total cellular resources for endosomal import, ${K}_{m}=\frac{{k}_{-1}}{{k}_{+1}}$. This is how CpG and pIC signaling pathways are originally modeled (Fig. 1). To incorporate the competition for endosomal transport resources, based on competition form of the Michaelis–Menten kinetics (Michaelis et. al, 2011), the rate should be updated as:

$$v({CpG})=\frac{{v}_{max ,{pIC}}* {{\rm{CpG}}}}{1+\frac{{CpG}}{{K}_{m,{CpG}}}+\frac{{pIC}}{{K}_{m,{pIC}}}}$$

$$v({pIC})=\frac{{v}_{max ,{pIC}}* {pIC}}{1+\frac{{pIC}}{{K}_{m,{pIC}}}+\frac{{CpG}}{{K}_{m,{CpG}}}}$$

Combining with the rest of the reactions in the signaling network, the full formula of these two equations are:

$$\frac{{{\rm{d}}}({{\rm{Cp}}}{{{\rm{G}}}}_{{{\rm{en}}}})}{{{\rm{d}}}t}= 	 -{\psi }_{89,1}* {{\rm{Cp}}}{{{\rm{G}}}}_{{{\rm{en}}}}-{\psi }_{90,1}* {{\rm{Cp}}}{{{\rm{G}}}}_{{{\rm{en}}}}* {{\rm{TLR}}}9\\ 	 +\frac{{v}_{max ,{pIC}}* {{\rm{CpG}}}}{1+\frac{{CpG}}{{K}_{m,{CpG}}}+\frac{{pIC}}{{K}_{m,{pIC}}}}+{\psi }_{91,1}* {{\rm{TLR}}}9{{\rm{CpG}}}$$

and

$$\frac{{{\rm{d}}}({{\rm{pI}}}{{{\rm{C}}}}_{{{\rm{en}}}})}{{{\rm{d}}}t}= 	 -{\psi }_{80,1}* {{\rm{pI}}}{{{\rm{C}}}}_{{{\rm{en}}}}-{\psi }_{81,1}* {{\rm{pI}}}{{{\rm{C}}}}_{{{\rm{en}}}}* {{\rm{TLR}}}3 \\ 	 +\frac{{v}_{max ,{pIC}}* {pIC}}{1+\frac{{pIC}}{{K}_{m,{pIC}}}+\frac{{CpG}}{{K}_{m,{CpG}}}}+{\psi }_{82,1}* {{\rm{TLR}}}3{{\rm{pIC}}}$$

These equations replaced the original ODE equations of $\frac{d({Cp}{G}_{{en}})}{{dt}}$ and $\frac{d({polyI}{C}_{{en}})}{{dt}}$ for the updated model.

### Stimulus-response specificity (SRS) assessment

To analyze stimulus-response specificity of NFκB dynamics across different stimulations, we computed the distributions of signaling codons from simulated datasets using the updated CpG-pIC competition model. Wasserstein-2 distances were computed between codon distributions across different conditions, and the average Wasserstein distance across six signaling codons was used to quantify stimulus-response specificity (Figs. 3A, 4E, and 5D).

A random forest classifier (Breiman, 2001) was trained on simulated data to predict conditions with NFκB signaling codons as input. The model was implemented using “RandomForestClassifier” in Python (Pedregosa et al, 2011). The dataset included 31 ligand-mixture conditions: five single ligands—TNF, LPS, CpG, pIC, and Pam—and 26 ligand combinations of these single ligands. The dataset was split into 67% training and 33% testing, comprising 30,990 virtual cells, with 999–1000 cells per condition.

Model performance was assessed using a confusion matrix, where predicted vs. true ligand classification accuracy was recorded. Evaluation metrics included sensitivity, precision, and F1 score (harmonic mean of precision and sensitivity) to measure per-class performance. Heatmaps were used to visualize classification trends and misclassification patterns.

For pair-ligand combinations and the corresponding single-ligand condition, the same classification approach was applied to the corresponding three conditions—two single ligands and their combination—for each pair. This resulted in a total of 10 unique base-paired-ligand combinations.

For the classifier on predicting specific ligand presence or not in ligand mixtures, we grouped all double-ligand, triple-ligand, and quadruple-ligand conditions (in total 25 simulation conditions) into two categories: one with 14 conditions where the specific ligand is present and another with 11 conditions where that ligand is absent. We then trained machine learning classifiers to predict whether a cell was exposed to that ligand based on its NFκB signaling codon. Five different classifiers were trained for five ligands (Fig. 3D). The same classification approach was performed for the experimental dataset (Fig. 3E).

Similarly, LSTM classifiers (Hochreiter and Schmidhuber, 1997) were used to classify conditions based on NFκB dynamic trajectories. This was implemented using “LSTM” in “tensorflow.keras.layers” in Python (Abadi et al, 2016). The dataset was split into 60% training, and 20% validation and 20% testing. All model performance metrics are calculated using the 20% validation + 20% testing, in total 40% of the dataset.

### Single-cell synergy and antagonism score

To explore how different ligand dose levels combine, for each of ten ligand pairs (e.g., TNF–LPS, Pam–pIC, and eight others; hereafter denoted ligand 1 and ligand 2), we selected five dose levels—from minimal through saturated, doses spaced logarithmically—plus a zero dose (Appendix Fig. S9). The dose values are listed in Appendix Table S1.

We then paired every dose of ligand 1 with every dose of ligand 2, yielding 36 combinations per pair. For each pair of ligands, 1000 cells were sampled using the same method (See Predicting heterogeneous single-cell responses to combinatorial-ligand stimulation). For each single cell, nuclear NFκB, IKK, TAK1, TRAF6, TRIF, MyD88, and C1 (TNF signaling complex), TLR4LPSen (LPS signaling complex on endosome), TLR4LPS (LPS signaling complex on membrane), TLR9_CpG (CpG signaling complex), TLR3_pIC (pIC signaling complex), and TLR2_P3CSK (Pam signaling complex) dynamics in response to each of the 36 combinations of the ligand 1 and ligand 2 with doses from D0 to D5 is then simulated and stored.

In total, 10,000 cells were sampled, across 360 unique combinatorial conditions. Specifically, 360,000 single-cell NFκB profiles, 360,000 single-cell IKK profiles, 360,000 single-cell TAK1 profiles, 324,000 single-cell MyD88 profiles, 360,000 single-cell TRAF6 profiles, 252,000 single-cell TRIF profiles, 144,000 single-cell C1 profiles, 144,000 single-cell TLR4LPS profiles, 144,000 single-cell TLR4LPSen profiles, 144,000 single-cell TLR2_P3CSK profiles, 144,000 single-cell TLR9_CpG profiles, and 144,000 single-cell TLR3_pIC profiles were produced (Fig. EV3, Steps 1–3; see Fig. S8 for simulated single-cell trajectories of an example LPS–Pam pair).

The synergy and antagonism score were defined based on Peak features of NFκB dynamics upon stimulation (Fig. EV3, Step 4). Nonresponsive cells with response upon stimuli less than fivefold of the basal level were filtered out (Fig. EV3, Step 5). For each responder cell, a synergy score and an antagonism score were defined as (Fig. EV3, Step 6):

$${{{\rm{Synergy\; Score}}}}=\frac{R\left(L1,L2\right)-R\left(0,0\right)}{\left(R\left(L1,0\right)-R\left(0,0\right)\right)+\left(R\left(0,L2\right)-R\left(0,0\right)\right)}$$

$${{{\rm{Antagonism\; Score}}}}=\frac{R\left(L1,L2\right)-R\left(0,0\right)}{\max \left(R\left(L1,0\right)-R\left(0,0\right),R\left(0,L2\right)-R\left(0,0\right)\right)}$$

where L1 and L2 are the doses of ligands 1 and 2, respectively; R(L1, L2) is the dynamic feature (Peak or Total) of NFκB signaling activity in response to the corresponding combination; R(0, 0) is the dynamic feature at zero doses (steady state) for that same cell; and R(L1, 0) and R(0, L2) are the dynamic features in response to each ligand alone for that same cell.

Plotting z = R(L1, L2) − R(0, 0) against x = R(L1, 0) − R(0, 0) and y = R(0, L2) − R(0, 0), cells with synergistic responses locate above the plane z = x + y, i.e., synergy score >1; antagonistic cells fall below the plane z = max(x, y), i.e., antagonism score <1 (Fig. EV3, Step 6). To identify cells with significant effects (and exclude those “synergistic/antagonistic cells” induced by numerical error), synergistic cells were defined as those with a synergy score >1.25, and antagonistic cells were defined as those with an antagonism score <0.9 (Fig. EV3, Step 7).

### Parameter sensitivity analysis

To quantify each parameter’s contribution to the single-cell synergy scores or antagonism scores, we employed PAWN, a global parameter sensitivity analysis algorithm, using single-cell parameters as independent variables/input of the model, and scores as dependent variables/output of the model. PAWN analysis is a distribution-based global sensitivity analysis method that quantifies how strongly each input parameter influences model output by measuring how much the conditional output distribution changes when that parameter is fixed compared with the unconditional output distribution (Khorashadi Zadeh et al, 2017; Pianosi and Wagener, 2015, 2018). PAWN analysis was implemented in Matlab.

### Statistics and reproducibility

#### Sample size

For simulation data, NFκB trajectories were generated from heterogeneous single-cell parameter sets obtained by bootstrap resampling of the empirical single-cell parameter distributions (see Methods, “Simulation of heterogeneous NFκB dynamical responses”) (Guo et al, 2025). For Figs. 1–3, 1000 virtual cells were simulated for each of the 31 single- and combinatorial-ligand conditions. For the dose-resolved synergy and antagonism analysis (Figs. 4, 5), 1000 cells were simulated for each of 360 conditions (10 ligand pairs × 36 dose combinations), yielding 360,000 single-cell trajectories (nine signaling species recorded per cell). Each virtual cell is an independent stochastic draw from the inferred parameter distribution; no statistical method was used to predetermine the number of simulated cells, which was set to match the experimental single-cell sample sizes and to stabilize the distribution estimates.

For experimental data, live-cell imaging of nuclear NFκB (RelA-mVenus) was performed in HoxB4-derived macrophages (hMPDMs). Each condition yielded ≈600–950 single-cell trajectories (exact n per condition in the Source Data) from seven independent imaging experiments. No statistical method was used to predetermine sample size; the number of cells per condition was determined by culture density, imaging throughput, and MACKtrack segmentation and tracking. Single cells within an imaging experiment are technical units. The single-ligand imaging experiments were representative of eleven biological replicates, some of which were reported in previous publications (Adelaja et al, 2021; Luecke et al, 2021; Rahman et al, 2024; Singh et al, 2024; Guo et al, 2025).

#### Inclusion and exclusion criteria

For experimental data, mitotic cells and cells that left the field of view during imaging were excluded. For the simulation-based synergy and antagonism analysis (Figs. 4, 5), non-responding cells (below the stated Peak threshold, or <5-fold over basal) were excluded so that synergy and antagonism scores were well defined (see Methods, “Single-cell synergy and antagonism score”). No other data were excluded.

#### Randomization and blinding

The study comprises computational simulations and automated single-cell image quantification, with identical pipelines applied to all conditions; no allocation of subjects to treatment groups was involved, so randomization and blinding are not applicable. Classifier data were partitioned at random (67/33% train/test for random forests; 60/20%/20% train/validation/test for LSTM).

#### Statistical tests

Differences between single-cell distributions (e.g., signaling codons, model parameters) were assessed with one-sided Mann–Whitney *U*-tests, a nonparametric test that does not require assuming a specific distributional form. Wasserstein-2 distances are descriptive; comparisons of stimulus-response specificity between alternative models (with vs without IKK ultrasensitivity; with vs without CpG-pIC antagonism) used paired Wilcoxon signed-rank tests over matched condition pairs of the average Wasserstein distance. Parameter sensitivities were quantified by PAWN global sensitivity analysis, using the Kolmogorov–Smirnov statistic between the conditional and unconditional output distributions with a randomly assigned dummy parameter as the negative-control significance threshold. Exact *p* values and the test used are reported in the corresponding figures.

## Supplementary information

Appendix

Peer Review File

Dataset EV1

Dataset EV2

Source data Fig. 1

Source data Fig. 2

Source data Fig. 3

Source data Fig. 4

Source data Fig. 5

Expanded View Figures

## Data Availability

All experimental and simulation data were available at Mendeley Data (https://doi.org/10.17632/hcg272hzyd.1). All code is available at GitHub (https://github.com/Xiaolu-Guo/Combinatorial_ligand_NFkB). The source data of this paper are collected in the following database record: biostudies:S-SCDT-10_1038-S44320-026-00230-9.

## References

[CR1] Abadi M, Barham P, Chen J, Chen Z, Davis A, Dean J, Devin M, Ghemawat S, Irving G, Isard M et al (2016) TensorFlow: a system for large-scale machine learning. In 12th USENIX Symposium on Operating Systems Design and Implementation (OSDI 16) pp 265–283. Savannah, GA: USENIX Association

[CR2] Adamson A, Boddington C, Downton P, Rowe W, Bagnall J, Lam C, Maya-Mendoza A, Schmidt L, Harper CV, Spiller DG et al (2016) Signal transduction controls heterogeneous NF-κB dynamics and target gene expression through cytokine-specific refractory states. Nat Commun 7:12057 10.1038/ncomms12057 PMC4935804 27381163

[CR3] Adelaja A, Taylor B, Sheu KM, Liu Y, Luecke S, Hoffmann A (2021) Six distinct NFκB signaling codons convey discrete information to distinguish stimuli and enable appropriate macrophage responses. Immunity 54:916–930.e7 10.1016/j.immuni.2021.04.011 PMC8184127 33979588

[CR4] Aderem A, Ulevitch RJ (2000) Toll-like receptors in the induction of the innate immune response. Nature 406:782–787 10.1038/35021228 10963608

[CR5] Ando M, Magi S, Seki M, Suzuki Y, Kasukawa T, Lefaudeux D, Hoffmann A, Okada M (2021) IκBα is required for full transcriptional induction of some NFκB-regulated genes in response to TNF in MCF-7 cells. NPJ Syst Biol Appl 7:42 10.1038/s41540-021-00204-7 PMC8636565 34853340

[CR6] Andridge RR, Little RJA (2010) A review of hot deck imputation for survey non-response. Int Stat Rev 78:40–64 10.1111/j.1751-5823.2010.00103.x PMC3130338 21743766

[CR7] Ashall L, Horton CA, Nelson DE, Paszek P, Harper CV, Sillitoe K, Ryan S, Spiller DG, Unitt JF, Broomhead DS et al (2009) Pulsatile stimulation determines timing and specificity of NF-κB-dependent transcription. Science 324:242–246 10.1126/science.1164860 PMC2785900 19359585

[CR8] Bain CC, Mowat AM (2014) Macrophages in intestinal homeostasis and inflammation. Immunol Rev 260:102–117 10.1111/imr.12192 PMC4141699 24942685

[CR9] Basak S, Behar M, Hoffmann A (2012) Lessons from mathematically modeling the NF-κB pathway. Immunol Rev 246:221–238 10.1111/j.1600-065X.2011.01092.x PMC3343698 22435558

[CR10] Bauer S, Kirschning CJ, Häcker H, Redecke V, Hausmann S, Akira S, Wagner H, Lipford GB (2001) Human TLR9 confers responsiveness to bacterial DNA via species-specific CpG motif recognition. Proc Natl Acad Sci USA 98:9237–9242 10.1073/pnas.161293498 PMC55404 11470918

[CR11] Breiman L (2001) Random forests. Mach Learn 45:5–32

[CR12] Chen ZJ (2005) Ubiquitin signalling in the NF-κB pathway. Nat Cell Biol 7:758–765 10.1038/ncb0805-758 PMC1551980 16056267

[CR13] Cheng QJ, Ohta S, Sheu KM, Spreafico R, Adelaja A, Taylor B, Hoffmann A (2021) NF-κB dynamics determine the stimulus specificity of epigenomic reprogramming in macrophages. Science 372:1349–1353 10.1126/science.abc0269 PMC8489855 34140389

[CR14] Cheng Z, Taylor B, Ourthiague DR, Hoffmann A (2015) Distinct single-cell signaling characteristics are conferred by the MyD88 and TRIF pathways during TLR4 activation. Sci Signal 8:ra69 10.1126/scisignal.aaa5208 PMC6764925 26175492

[CR15] Cheong R, Rhee A, Wang CJ, Nemenman I, Levchenko A (2011) Information transduction capacity of noisy biochemical signaling networks. Science 334:354–358 10.1126/science.1204553 PMC3895446 21921160

[CR16] Covert MW, Leung TH, Gaston JE, Baltimore D (2005) Achieving stability of lipopolysaccharide-induced NF-kappaB activation. Science 309:1854–1857 10.1126/science.1112304 16166516

[CR17] Cruz JA, Mokashi CS, Kowalczyk GJ, Guo Y, Zhang Q, Gupta S, Schipper DL, Smeal SW, Lee REC (2021) A variable-gain stochastic pooling motif mediates information transfer from receptor assemblies into NF-κB. Sci Adv 7:eabi9410 10.1126/sciadv.abi9410 PMC8302133 34301608

[CR18] Davies AE, Pargett M, Siebert S, Gillies TE, Choi Y, Tobin SJ, Ram AR, Murthy V, Juliano C, Quon G et al (2020) Systems-level properties of EGFR-RAS-ERK signaling amplify local signals to generate dynamic gene expression heterogeneity. Cell Syst 11:161–175.e5 10.1016/j.cels.2020.07.004 PMC7856305 32726596

[CR19] Delyon B, Lavielle M, Moulines E (1999) Convergence of a stochastic approximation version of the EM algorithm. Ann Stat 27:94–128

[CR20] Dharmarajan L, Kaltenbach H-M, Rudolf F, Stelling J (2019) A simple and flexible computational framework for inferring sources of heterogeneity from single-cell dynamics. Cell Syst 8:15–26.e11 10.1016/j.cels.2018.12.007 30638813

[CR21] D’Orazio M, Zio MD, Scanu M (2006) Statistical matching: theory and practice. John Wiley & Sons

[CR22] Guo X, Adelaja A, Singh A, Roy W, Hoffmann A (2025) Modeling heterogeneous signaling dynamics of macrophages reveals principles of information transmission in stimulus responses. Nat Commun 16:5986 10.1038/s41467-025-60901-3 PMC12218168 40593748

[CR24] Gutschow MV, Mason JC, Lane KM, Maayan I, Hughey JJ, Bajar BT, Amatya DN, Valle SD, Covert MW (2019) Combinatorial processing of bacterial and host-derived innate immune stimuli at the single-cell level. Mol Biol Cell 30:282–292 10.1091/mbc.E18-07-0423 PMC6589564 30462580

[CR25] Hanson RL, Batchelor E (2022) Coordination of MAPK and p53 dynamics in the cellular responses to DNA damage and oxidative stress. Mol Syst Biol 18:e11401 10.15252/msb.202211401 PMC9724178 36472304

[CR26] Hegarty LM, Jones G-R, Bain CC (2023) Macrophages in intestinal homeostasis and inflammatory bowel disease. Nat Rev Gastroenterol Hepatol 20:538–553 10.1038/s41575-023-00769-0 37069320

[CR27] Heldwein KA, Liang MD, Andresen TK, Thomas KE, Marty AM, Cuesta N, Vogel SN, Fenton MJ (2003) TLR2 and TLR4 serve distinct roles in the host immune response against Mycobacterium bovis BCG. J Leukoc Biol 74:277–286 10.1189/jlb.0103026 12885945

[CR28] Hochreiter S, Schmidhuber J (1997) Long short-term memory. Neural Comput 9:1735–1780 10.1162/neco.1997.9.8.1735 9377276

[CR29] Hoffmann A, Levchenko A, Scott ML, Baltimore D (2002) The IkappaB-NF-kappaB signaling module: temporal control and selective gene activation. Science 298:1241–1245 10.1126/science.1071914 12424381

[CR30] Hu Y-B, Dammer EB, Ren R-J, Wang G (2015) The endosomal-lysosomal system: from acidification and cargo sorting to neurodegeneration. Transl Neurodegener 4:18 10.1186/s40035-015-0041-1 PMC4596472 26448863

[CR31] Huang Y, Zhang Q, Lubas M, Yuan Y, Yalcin F, Efe IE, Xia P, Motta E, Buonfiglioli A, Lehnardt S et al (2020) Synergistic Toll-like receptor 3/9 signaling affects properties and impairs glioma-promoting activity of microglia. J Neurosci 40:6428–6443 10.1523/JNEUROSCI.0666-20.2020 PMC7424875 32631940

[CR32] Hughey JJ, Gutschow MV, Bajar BT, Covert MW (2015) Single-cell variation leads to population invariance in NF-κB signaling dynamics. Mol Biol Cell 26:583–590 10.1091/mbc.E14-08-1267 PMC4310747 25473117

[CR33] Kawai T, Akira S (2011) Toll-like receptors and their crosstalk with other innate receptors in infection and immunity. Immunity 34:637–650 10.1016/j.immuni.2011.05.006 21616434

[CR34] Kellogg RA, Tian C, Etzrodt M, Tay S (2017) Cellular decision making by non-integrative processing of TLR inputs. Cell Rep 19:125–135 10.1016/j.celrep.2017.03.027 PMC5766323 28380352

[CR35] Khorashadi Zadeh F, Nossent J, Sarrazin F, Pianosi F, van Griensven A, Wagener T, Bauwens W (2017) Comparison of variance-based and moment-independent global sensitivity analysis approaches by application to the SWAT model. Environ Model Softw 91:210–222

[CR36] Komarova NL, Zou X, Nie Q, Bardwell L (2005) A theoretical framework for specificity in cell signaling. Mol Syst Biol 1:2005.0023 10.1038/msb4100031 PMC1681467 16729058

[CR37] Kumar R, Clermont G, Vodovotz Y, Chow CC (2004) The dynamics of acute inflammation. J Theor Biol 230:145–155 10.1016/j.jtbi.2004.04.044 15321710

[CR38] Lane K, Andres-Terre M, Kudo T, Monack DM, Covert MW (2019) Escalating threat levels of bacterial infection can be discriminated by distinct MAPK and NF-κB signaling dynamics in single host cells. Cell Syst 8:183–196.e4 10.1016/j.cels.2019.02.008 30904375

[CR39] Lavielle M, Mbogning C (2014) An improved SAEM algorithm for maximum likelihood estimation in mixtures of non linear mixed effects models. Stat Comput 24:693–707

[CR40] Lavin Y, Mortha A, Rahman A, Merad M (2015) Regulation of macrophage development and function in peripheral tissues. Nat Rev Immunol 15:731–744 10.1038/nri3920 PMC4706379 26603899

[CR41] Lee REC, Walker SR, Savery K, Frank DA, Gaudet S (2014) Fold change of nuclear NF-κB determines TNF-induced transcription in single cells. Mol Cell 53:867–879 10.1016/j.molcel.2014.01.026 PMC3977799 24530305

[CR42] Lindstrom ML, Bates DM (1990) Nonlinear mixed effects models for repeated measures data. Biometrics 46:673–687 2242409

[CR43] Llamosi A, Gonzalez-Vargas AM, Versari C, Cinquemani E, Ferrari-Trecate G, Hersen P, Batt G (2016) What population reveals about individual cell identity: single-cell parameter estimation of models of gene expression in yeast. PLoS Comput Biol 12:e1004706 10.1371/journal.pcbi.1004706 PMC4747589 26859137

[CR44] Luecke S, Guo X, Sheu KM, Singh A, Lowe SC, Han M, Diaz J, Lopes F, Wollman R, Hoffmann A (2024) Dynamical and combinatorial coding by MAPK p38 and NFκB in the inflammatory response of macrophages. Mol Syst Biol 20:898–932 10.1038/s44320-024-00047-4 PMC11297158 38872050

[CR45] Luecke S, Sheu KM, Hoffmann A (2021) Stimulus-specific responses in innate immunity: multilayered regulatory circuits. Immunity 54:1915–1932 10.1016/j.immuni.2021.08.018 PMC8452151 34525335

[CR46] Michaelis L, Menten ML, Johnson KA, Goody RS (2011) The original Michaelis constant: translation of the 1913 Michaelis-Menten paper. Biochemistry 50:8264–8269 10.1021/bi201284u PMC3381512 21888353

[CR47] Mori T, Takahashi K, Naito M, Kodama T, Hakamata H, Sakai M, Miyazaki A, Horiuchi S, Ando M (1994) Endocytic pathway of scavenger receptors via trans-Golgi system in bovine alveolar macrophages. Lab Invest 71:409–416 7933991

[CR48] Murshid A, Borges TJ, Lang BJ, Calderwood SK (2016) The scavenger receptor SREC-I cooperates with toll-like receptors to trigger inflammatory innate immune responses. Front Immunol 7:226 10.3389/fimmu.2016.00226 PMC4904184 27379091

[CR49] Nelson DE, Ihekwaba AEC, Elliott M, Johnson JR, Gibney CA, Foreman BE, Nelson G, See V, Horton CA, Spiller DG et al (2004) Oscillations in NF-κB signaling control the dynamics of gene expression. Science 306:704–708 10.1126/science.1099962 15499023

[CR50] Newton K, Dixit VM (2012) Signaling in innate immunity and inflammation. Cold Spring Harb Perspect Biol 4:a006049 10.1101/cshperspect.a006049 PMC3282411 22296764

[CR51] O’Sullivan MJ, Lindsay AJ (2020) The endosomal recycling pathway-at the crossroads of the cell. Int J Mol Sci 21:6074 10.3390/ijms21176074 PMC7503921 32842549

[CR52] Paek AL, Liu JC, Loewer A, Forrester WC, Lahav G (2016) Cell-to-cell variation in p53 dynamics leads to fractional killing. Cell 165:631–642 10.1016/j.cell.2016.03.025 PMC5217463 27062928

[CR54] Pedregosa F, Varoquaux G, Gramfort A, Michel V, Thirion B, Grisel O, Blondel M, Prettenhofer P, Weiss R, Dubourg V et al (2011) Scikit-learn: machine learning in Python. J Mach Learn Res 12:2825–2830

[CR55] Peterson AF, Ingram K, Huang EJ, Parksong J, McKenney C, Bever GS, Regot S (2022) Systematic analysis of the MAPK signaling network reveals MAP3K-driven control of cell fate. Cell Syst 13:885–894.e4 10.1016/j.cels.2022.10.003 PMC9923805 36356576

[CR79] Peyré G, Cuturi, M. (2019) Computational Optimal Transport with Applications to Data Sciences. Foundations and Trends® in Machine Learning 11:355-607

[CR56] Pianosi F, Wagener T (2015) A simple and efficient method for global sensitivity analysis based on cumulative distribution functions. Environ Model Softw 67:1–11

[CR57] Pianosi F, Wagener T (2018) Distribution-based sensitivity analysis from a generic input-output sample. Environ Model Softw 108:197–207

[CR58] Rahman SMT, Singh A, Lowe S, Aqdas M, Jiang K, Vaidehi Narayanan H, Hoffmann A, Sung M-H (2024) Co-imaging of RelA and c-Rel reveals features of NF-κB signaling for ligand discrimination. Cell Rep 43:113940 10.1016/j.celrep.2024.113940 PMC11015162 38483906

[CR59] Ruedl C, Khameneh HJ, Karjalainen K (2008) Manipulation of immune system via immortal bone marrow stem cells. Int Immunol 20:1211–1218 10.1093/intimm/dxn079 18644831

[CR60] Selimkhanov J, Taylor B, Yao J, Pilko A, Albeck J, Hoffmann A, Tsimring L, Wollman R (2014) Accurate information transmission through dynamic biochemical signaling networks. Science 346:1370–1373 10.1126/science.1254933 PMC4813785 25504722

[CR61] Sen S, Cheng Z, Sheu KM, Chen YH, Hoffmann A (2020) Gene regulatory strategies that decode the duration of NFκB dynamics contribute to LPS- versus TNF-specific gene expression. Cell Syst 10:169–182.e5 10.1016/j.cels.2019.12.004 PMC7047529 31972132

[CR62] Sheu K, Luecke S, Hoffmann A (2019) Stimulus-specificity in the responses of immune sentinel cells. Curr Opin Syst Biol 18:53–61 10.1016/j.coisb.2019.10.011 PMC7450653 32864512

[CR63] Sheu KM, Guru AA, Hoffmann A (2023) Quantifying stimulus-response specificity to probe the functional state of macrophages. Cell Syst 14:180–195.e5 10.1016/j.cels.2022.12.012 PMC10023480 36657439

[CR64] Sheu KM, Hoffmann A (2022) Functional hallmarks of healthy macrophage responses: their regulatory basis and disease relevance. Annu Rev Immunol 40:295–321 10.1146/annurev-immunol-101320-031555 PMC10074967 35471841

[CR65] Sheu KM, Pimplaskar A, Hoffmann A (2024) Single-cell stimulus-response gene expression trajectories reveal the stimulus specificities of dynamic responses by single macrophages. Mol Cell 84:4095–4110.e6 10.1016/j.molcel.2024.09.023 PMC11560543 39413794

[CR66] Singh A, Sen S, Iter M, Adelaja A, Luecke S, Guo X & Hoffmann A (2024) Stimulus-response signaling dynamics characterize macrophage polarization states. Cell Syst 15:563-577.e6 10.1016/j.cels.2024.05.002 PMC11226196 38843840

[CR67] Son M, Wang AG, Keisham B, Tay S (2023) Processing stimulus dynamics by the NF-κB network in single cells. Exp Mol Med 55:2531–2540 10.1038/s12276-023-01133-7 PMC10766959 38040923

[CR68] Stephen SL, Freestone K, Dunn S, Twigg MW, Homer-Vanniasinkam S, Walker JH, Wheatcroft SB, Ponnambalam S (2010) Scavenger receptors and their potential as therapeutic targets in the treatment of cardiovascular disease. Int J Hypertens 2010:646929 10.4061/2010/646929 PMC2958427 20981357

[CR69] Stuart T, Butler A, Hoffman P, Hafemeister C, Papalexi E, Mauck WM, Hao Y, Stoeckius M, Smibert P, Satija R (2019) Comprehensive integration of single-cell data. Cell 177:1888–1902.e21 10.1016/j.cell.2019.05.031 PMC6687398 31178118

[CR70] Suderman R, Bachman JA, Smith A, Sorger PK, Deeds EJ (2017) Fundamental trade-offs between information flow in single cells and cellular populations. Proc Natl Acad Sci USA 114:5755–5760 10.1073/pnas.1615660114 PMC5465904 28500273

[CR71] Sung M-H, Salvatore L, Lorenzi RD, Indrawan A, Pasparakis M, Hager GL, Bianchi ME, Agresti A (2009) Sustained oscillations of NF-κB produce distinct genome scanning and gene expression profiles. PLoS ONE 4:e7163 10.1371/journal.pone.0007163 PMC2747007 19787057

[CR72] Tang Y, Hoffmann A (2022) Quantifying information of intracellular signaling: progress with machine learning. Rep Prog Phys 85 10.1088/1361-6633/ac7a4a PMC9507437 35724636

[CR73] Tay S, Hughey JJ, Lee TK, Lipniacki T, Quake SR, Covert MW (2010) Single-cell NF-kappaB dynamics reveal digital activation and analogue information processing. Nature 466:267–271 10.1038/nature09145 PMC3105528 20581820

[CR74] Wang W, Zhang Y, Dettinger P, Reimann A, Kull T, Loeffler D, Manz MG, Lengerke C, Schroeder T (2021) Cytokine combinations for human blood stem cell expansion induce cell-type– and cytokine-specific signaling dynamics. Blood 138:847–857 10.1182/blood.2020008386 33988686

[CR75] Werner SL, Barken D, Hoffmann A (2005) Stimulus specificity of gene expression programs determined by temporal control of IKK activity. Science 309:1857–1861 10.1126/science.1113319 16166517

[CR76] Werner SL, Kearns JD, Zadorozhnaya V, Lynch C, O’Dea E, Boldin MP, Ma A, Baltimore D, Hoffmann A (2008) Encoding NF-kappaB temporal control in response to TNF: distinct roles for the negative regulators IkappaBalpha and A20. Genes Dev 22:2093–2101 10.1101/gad.1680708 PMC2492747 18676814

[CR77] Wertz IE, Dixit VM (2010) Signaling to NF-kappaB: regulation by ubiquitination. Cold Spring Harb Perspect Biol 2:a003350 10.1101/cshperspect.a003350 PMC2829959 20300215

[CR78] Zhang Q, Gupta S, Schipper DL, Kowalczyk GJ, Mancini AE, Faeder JR, Lee REC (2017) NF-κB dynamics discriminate between TNF doses in single cells. Cell Systems 5:638–645.e5 10.1016/j.cels.2017.10.011 PMC5746429 29128333

