# Supplementary material for: Macrophage response specificity to ligand mixtures is improved by signaling pathway antagonism
Source: Mol Syst Biol. 2026 Jul 29;22(10):1578–604. doi: 10.1038/s44320-026-00230-9 (PMC13639095; doi:10.1038/s44320-026-00230-9)
Supplement: Supplementary file 1 [file 44320_2026_230_MOESM10_ESM.pdf]

Expanded View Figures

Figure EV1. Model simulations predict NFκB responses to combinatorial ligand stimulation.

(**A**) Heatmaps of simulated single-cell NFκB trajectories under single-ligand (training dataset, in the red box) and combinatorial ligand (prediction dataset, in the blue box) stimulation. For each heatmap, the condition is indicated on the left, and each row within the heatmap represents a single-cell prediction, ordered by the total activity of the signaling. In each subpanel, the x-axis represents time, and color intensity indicates NFκB abundance in accordance with the color bar. (**B**) Violin plots of signaling codon distributions from single-ligand stimulation (training) to combinatorial ligand stimulation (prediction). On the left, plots illustrate the high and low values for each signaling codon to illustrate the dynamic features each signaling codon captures and quantifies. Conditions are labeled at the bottom. The mean value of the respective signaling codon distribution is indicated with "-". The red box indicates single-ligand conditions (training dataset), and the blue box indicates the combinatorial-ligand conditions (prediction dataset). For each condition, $n = 1000$ simulated single cells. (**C**) Heatmaps of experimental single-cell NFκB trajectories under single-ligand (top row) and pairwise combinatorial ligand (bottom row) stimulation. Within each heatmap, each row represents a single-cell prediction (ordered by total signaling activity); the x-axis represents time, and color intensity indicates NFκB abundance in accordance with the color bar in panel (**A**).

© The Author(s)

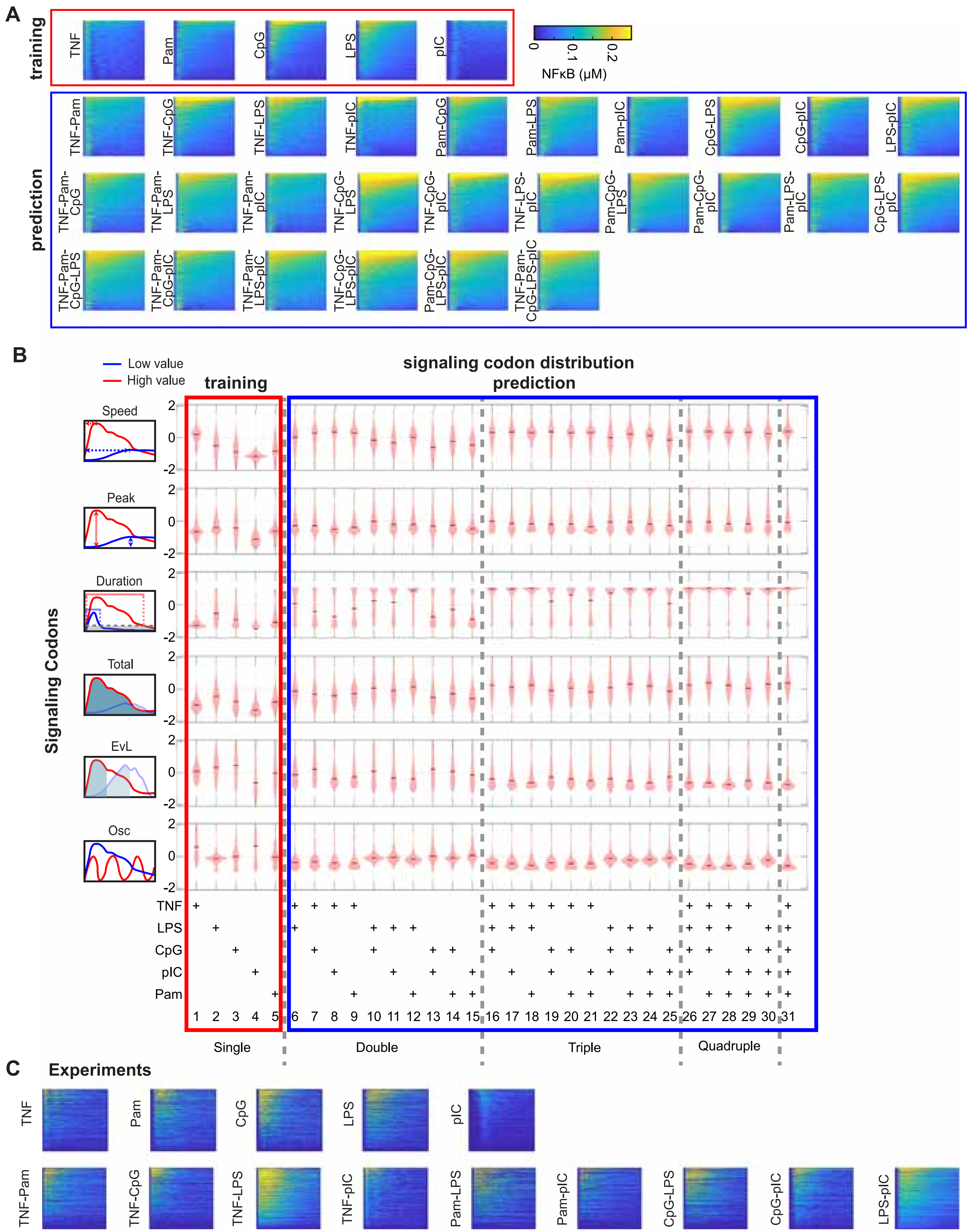
A
training
TNF
Pam
CpG
LPS
pIC
0
0.1
0.2
NFκB (μM)
prediction
TNF-Pam
TNF-CpG
TNF-LPS
TNF-pIC
Pam-CpG
Pam-LPS
Pam-pIC
CpG-LPS
CpG-pIC
LPS-pIC
TNF-Pam-CpG
TNF-Pam-LPS
TNF-Pam-pIC
TNF-CpG-LPS
TNF-CpG-pIC
TNF-LPS-pIC
Pam-CpG-LPS
Pam-CpG-pIC
Pam-LPS-pIC
CpG-LPS-pIC
TNF-Pam-CpG-LPS
TNF-Pam-CpG-pIC
TNF-Pam-LPS-pIC
TNF-CpG-LPS-pIC
Pam-CpG-LPS-pIC
TNF-Pam-CpG-LPS-pIC
B
Low value
High value
training
signaling codon distribution prediction
Signaling Codons
Speed
Peak
Duration
Total
EvL
Osc
2
0
-2
TNF
LPS
CpG
pIC
Pam
1 2 3 4 5 6 7 8 9 10 11 12 13 14 15 16 17 18 19 20 21 22 23 24 25 26 27 28 29 30 31
Single
Double
Triple
Quadruple
C
Experiments
TNF
Pam
CpG
LPS
pIC
TNF-Pam
TNF-CpG
TNF-LPS
TNF-pIC
Pam-LPS
Pam-pIC
CpG-LPS
CpG-pIC
LPS-pIC

 © The Author(s)

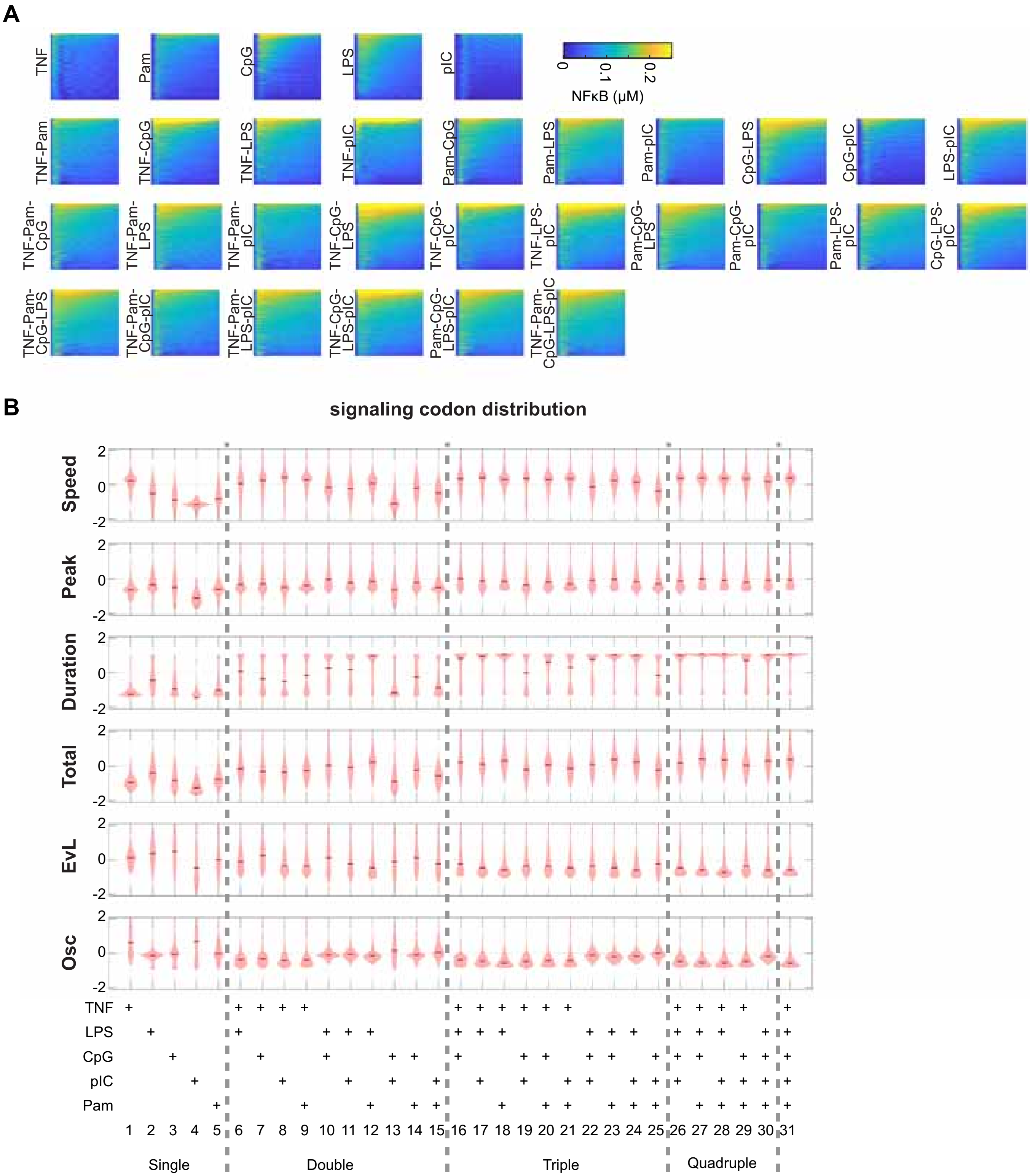

Figure EV2. Updated model simulations of combinatorial ligand stimulations.

(**A**) Heatmaps of simulated single-cell NFκB trajectories under single and all combinations of ligand stimulation using the updated model (Fig. 2D). For each heatmap, the condition is indicated on the left, and each row within the heatmap represents a single-cell prediction, ordered by Total signaling activity. In each subpanel, the x-axis represents time, and color intensity indicates NFκB abundance in accordance with the color bar. (**B**) Violin plots of signaling codon distributions from signaling trajectories shown in panel (**A**). Conditions are labeled at bottom. The mean value of the respective codon distribution is indicated. For each condition, $n = 1000$ simulated single cells.

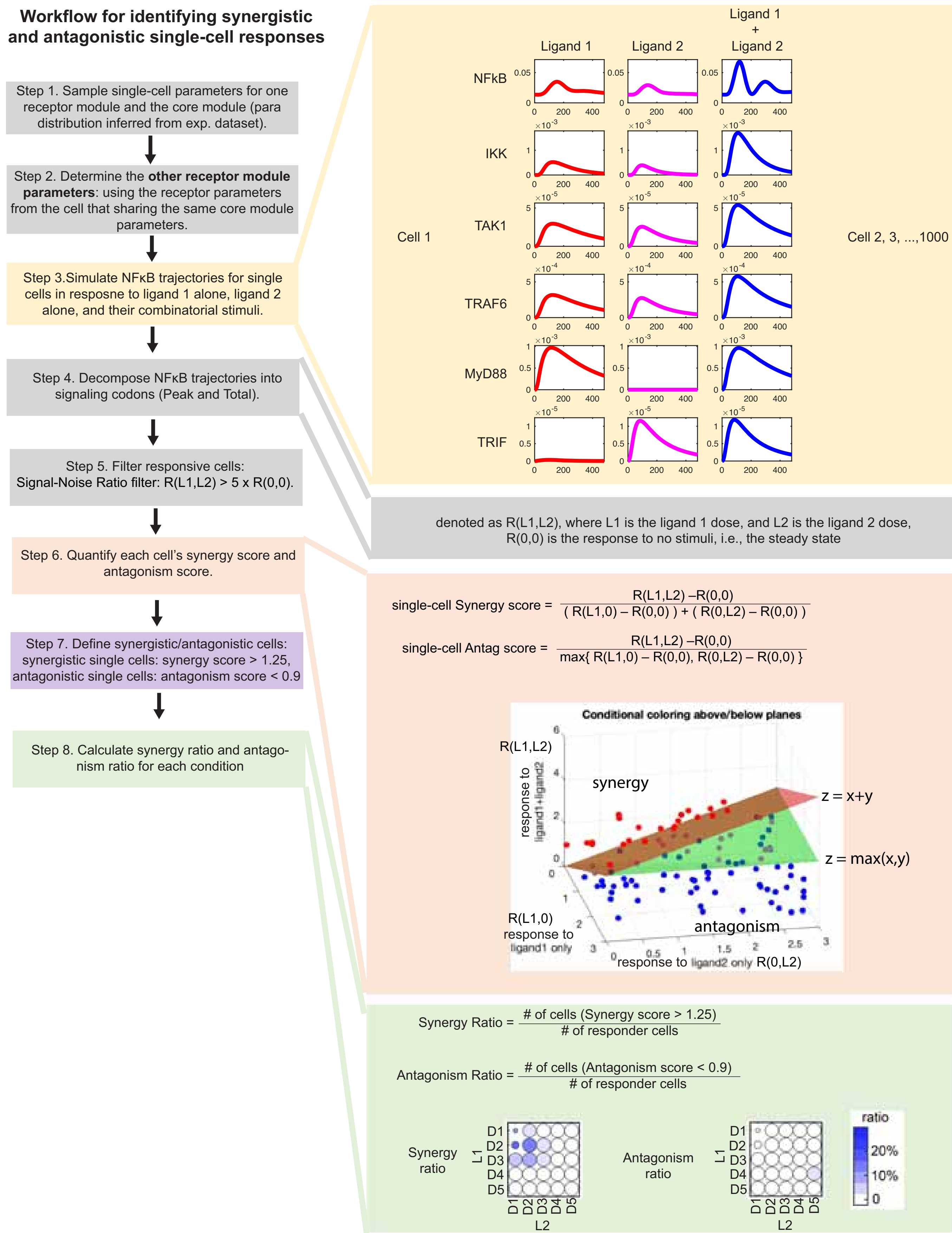

Workflow for identifying synergistic and antagonistic single-cell responses
Step 1. Sample single-cell parameters for one receptor module and the core module (para distribution inferred from exp. dataset).
Step 2. Determine the **other receptor module parameters**: using the receptor parameters from the cell that sharing the same core module parameters.
Step 3.Simulate NFκB trajectories for single cells in resposne to ligand 1 alone, ligand 2 alone, and their combinatorial stimuli.
Step 4. Decompose NFκB trajectories into signaling codons (Peak and Total).
Step 5. Filter responsive cells:
Signal-Noise Ratio filter: R(L1,L2) > 5 x R(0,0).
Step 6. Quantify each cell's synergy score and antagonism score.
Step 7. Define synergistic/antagonistic cells:
synergistic single cells: synergy score > 1.25,
antagonistic single cells: antagonism score < 0.9
Step 8. Calculate synergy ratio and antagonism ratio for each condition
Ligand 1
Ligand 2
Ligand 1 + Ligand 2
NFkB
IKK
TAK1
TRAF6
MyD88
TRIF
Cell 1
Cell 2, 3, ...,1000
denoted as R(L1,L2), where L1 is the ligand 1 dose, and L2 is the ligand 2 dose,
R(0,0) is the response to no stimuli, i.e., the steady state
single-cell Synergy score = R(L1,L2) –R(0,0) / (R(L1,0) – R(0,0)) + (R(0,L2) – R(0,0))
single-cell Antag score = R(L1,L2) –R(0,0) / max{ R(L1,0) – R(0,0), R(0,L2) – R(0,0) }
Conditional coloring above/below planes
R(L1,L2)
response to ligand1+ligand2
synergy
z = x+y
z = max(x,y)
antagonism
R(L1,0)
response to ligand1 only
response to ligand2 only R(0,L2)
Synergy Ratio = # of cells (Synergy score > 1.25) / # of responder cells
Antagonism Ratio = # of cells (Antagonism score < 0.9) / # of responder cells
Synergy ratio
Antagonism ratio
L1
D1
D2
D3
D4
D5
L2
ratio
20%
10%
0

 © The Author(s)

Figure EV3. Workflow for simulating single-cell signaling dynamics and calculating the synergy and antagonism score.

Left is the description of the workflow, and right are illustrations of key steps described in the workflow. Step 1 corresponds to the Methods section "Simulation of heterogenous NFκB dynamical responses"; Step 2 corresponds to the Methods section "Predicting heterogeneous single-cell responses to combinatorial-ligand stimulation"; Steps 3–7 correspond to the Methods section "Single-cell synergy and antagonism score". On the right, the illustration for Step 3 shows the generated simulation for individual cells (cell 1 as an example; in total, we generated 1000 virtual cells for each ligand pair). Within the cell 1 results, in each subpanel, the x-axis represents time; the y-axis shows the simulated species (indicated on the left) under three example conditions: applying the first ligand only, applying the second ligand only, or applying their combination. Illustration for Step 6: the synergy and antagonism score equations are the same as in the main text, Results "Ultrasensitive IKK activation underlies synergy among low-dose ligands" and "Kinetic competition can lead to antagonism between high-dose ligands and enhance signaling specificity of ligand mixtures," where R is the peak response for the corresponding stimuli (L1 represents the first ligand dose, L2 represents the second ligand dose; 0 represents the corresponding ligand not applied, i.e., 0 dose). Responses for the single-ligand conditions and the combinatorial condition are plotted in 3D space, with R(L1,0) − R(0,0) as the x-axis, R(0, L2) − R(0,0) as the y-axis, and R(L1, L2) − R(0,0) as the z-axis. The red dots illustrate synergistic cells, the blue dots illustrate antagonistic cells, and the gray dots illustrate non-synergistic/non-antagonistic cells. The two planes $z = x + y$ and $z = \max(x,y)$ separate synergistic, antagonistic, and non-synergistic/non-antagonistic cells. Illustration for Step 8: for each ligand pair, the fractions of synergistic and antagonistic cells are calculated and plotted as bubble plots, where circle size indicates the number of responding cells, and color indicates the fraction of synergistic/antagonistic cells (Figs. 4A and 5A).

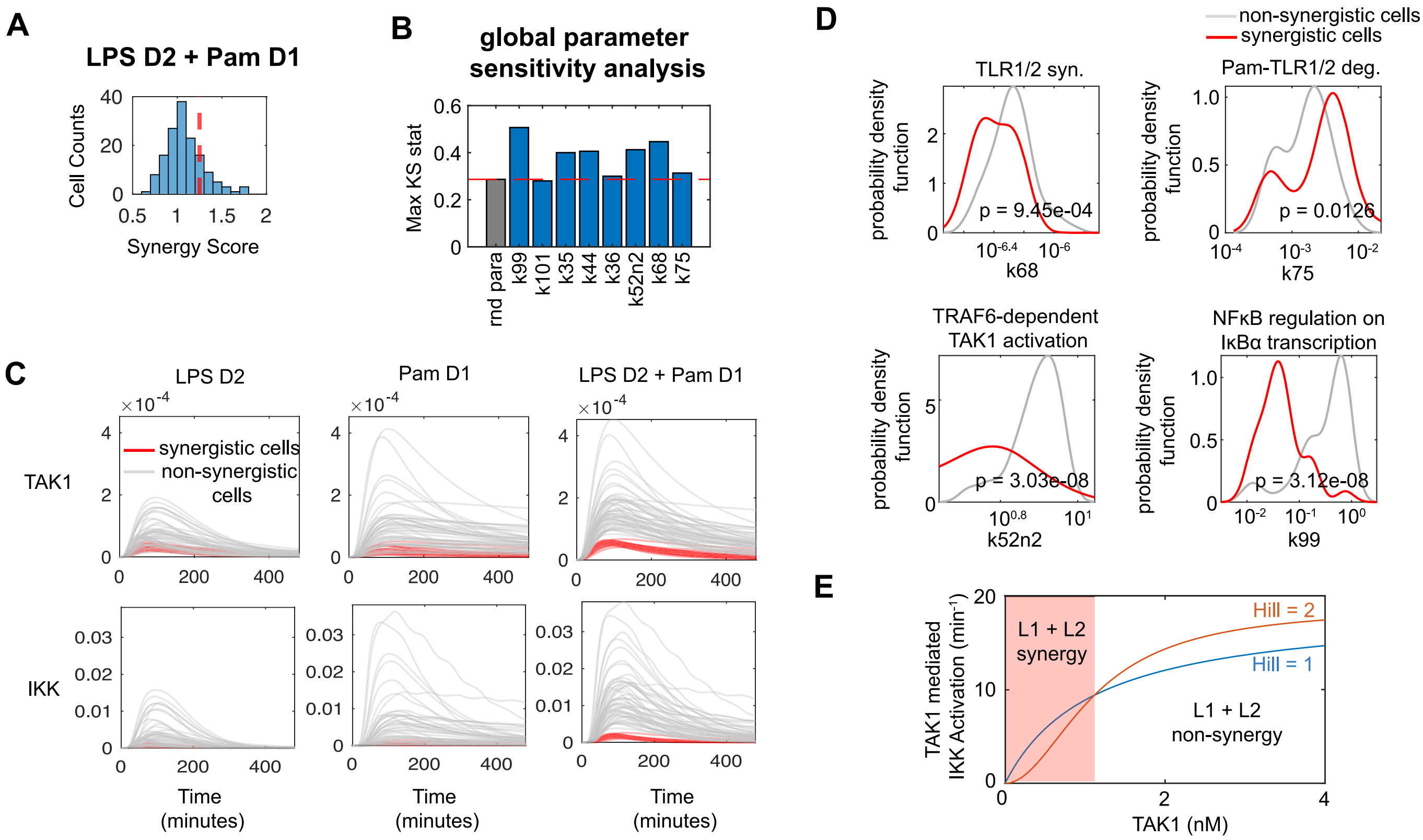

Figure EV4. Conditions for single-cell synergy under LPS D2 + Pam D1 co-stimulation.

(**A**) Histogram of synergy scores for responsive cells under combined stimulus conditions of Dose-2 (D2) LPS and Dose 1 (D1) Pam. (**B**) Bar plot indicating the feature importance score of each parameter, as evaluated via PAWN parameter analysis for the condition of D2 LPS + D1 Pam. The KS stat is the Kolmogorov–Smirnov distance between the unconditional output distribution and the output distribution conditioned on a given parameter, with a larger KS value indicating a stronger influence of that parameter on the model output. The parameter index is the same as in Fig. 4B. "rnd para" denotes a randomly assigned variable included in the model as a negative control, and its corresponding KS statistic provides a baseline for comparison with the sensitivities of the actual model parameters. (**C**) Single-cell time-series trajectories plot of TAK1 (top row) and IKK (Bottom row) activation for synergistic cells (synergy score >1.25, red lines) and non-synergistic cells (synergy score <1, gray lines) in response to D2 LPS (left panel), D1 Pam (middle panel), or their combination (right panel). (**D**) Distribution curves of parameter functions—metrics derived from parameter combinations linked to specific biological functions—for synergistic cells (red curve, synergy score >1.25) and non-synergistic cells (gray curve, synergy score <1). Each parameter function is labeled on the x-axis, with its numeric values indicated. The y-axis shows the probability density, and the biological relevance of each parameter function is noted in the title. Associated *p*-values were calculated using one-sided Mann–Whitney *U*-tests. (**E**) Hill equation curves of TAK1-mediated IKK activation. For Hill coefficient = 2, if L1–L2 responses fall within the red-shaded region, synergy between L1 and L2 may occur.

 © The Author(s)

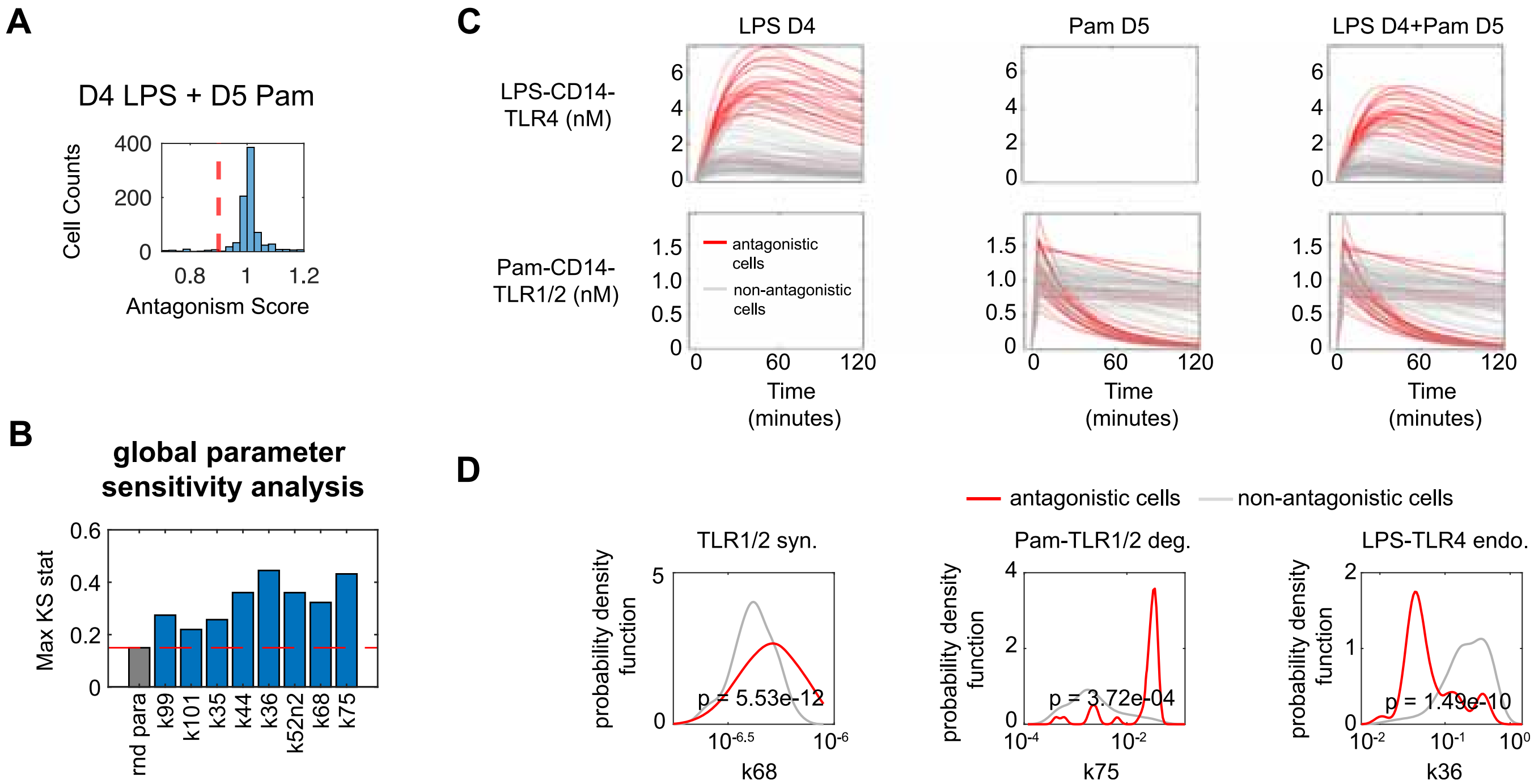

Figure EV5. Conditions for single-cell antagonism under LPS D4 + Pam D5 co-stimulation.

(**A**) Histogram of antagonism scores for responsive cells under combined stimulus conditions of Dose 4 (D4) LPS and Dose 5 (D5) Pam. (**B**) Bar plot indicating the feature importance score of each parameter, as evaluated via PAWN parameter analysis for condition of D4 LPS + D5 Pam. The KS stat is the Kolmogorov–Smirnov distance between the unconditional output distribution and the output distribution conditioned on a given parameter, with a larger KS value indicating a stronger influence of that parameter on the model output. The parameter index is the same as in Fig. 4B. "rnd para" denotes a randomly assigned variable included in the model as a negative control, and its corresponding KS statistic provides a baseline for comparison with the sensitivities of the actual model parameters. (**C**) Single-cell time-series trajectories plot of LPS-CD14-TLR4 (top row) and Pam-CD14-TLR1/2 (Bottom row) activation for antagonistic cells (antagonism score <0.9, red lines) and non-antagonistic cells (antagonism score >1.1, gray lines) in response to D4 LPS (left panel), D5 Pam (middle panel), or their combination (right panel). (**D**) Distribution curves of parameters for antagonistic cells (red curve, antagonism score <0.9) and non- antagonistic cells (gray curve, antagonism score >1.1). Each parameter function is labeled on the x-axis, with its numeric values indicated. The y-axis shows the probability density, and the biological relevance of each parameter function is noted in the title. Associated *p* values were calculated using one-sided Mann–Whitney *U*-tests.

© The Author(s)
