# Supplementary material for: Macrophage response specificity to ligand mixtures is improved by signaling pathway antagonism
Source: Mol Syst Biol. 2026 Jul 29;22(10):1578–604. doi: 10.1038/s44320-026-00230-9 (PMC13639095; doi:10.1038/s44320-026-00230-9)
Supplement: Supplementary file 2 [file 44320_2026_230_MOESM1_ESM.pdf]

Appendix for

Macrophage response specificity to ligand mixtures is improved by signaling pathway antagonism

Table of Contents

APPENDIX FIGURE S1. SCHEMATIC OF THE NFκB SIGNALING NETWORK MODEL 2
APPENDIX FIGURE S2. RESPONDER RATIOS OF NFκB SIGNALING UNDER SINGLE- AND COMBINATORIAL-LIGAND CONDITIONS 3
APPENDIX FIGURE S3. NFκB SIGNALING CODONS QUANTIFIED FROM MODEL SIMULATIONS AND EXPERIMENTAL STUDIES REVEAL SOME LIGAND COMBINATION TO BE INTEGRATIVE AND OTHERS NON-INTEGRATIVE 4
APPENDIX FIGURE S4. CLASSIFIER PREDICTIONS OF COMBINATORIAL LIGAND RESPONSES 5
APPENDIX FIGURE S5. PREDICTING STIMULI USING A LONG SHORT-TERM MEMORY (LSTM) MODEL 7
APPENDIX FIGURE S6. CLASSIFIER PREDICTIONS OF LIGAND PRESENCE UNDER MULTI-LIGAND CONDITIONS 8
APPENDIX FIGURE S7. SELECTING FUNCTIONALLY RELEVANT DOSES FOR EACH LIGAND BASED ON NFκB SIGNALING CODON DISTRIBUTIONS. 9
APPENDIX FIGURE S8. EXPLORING THE DYNAMICS OF KEY SIGNALING NODES WITHIN THE NFκB ACTIVATION PATHWAY. . 10
APPENDIX FIGURE S9. SINGLE-CELL SYNERGY ANALYSIS UNDER COMBINED LPS + PAM STIMULATION 11
APPENDIX FIGURE S10. REPRESENTATIVE CELL SIGNALING DYNAMICS UNDER DIFFERENT HILL COEFFICIENT OF TAK1- ... 12
MEDIATED IKK ACTIVATION. 12
APPENDIX FIGURE S11. SINGLE-CELL NFκB DYNAMIC PROFILES UNDER SINGLE AND COMBINATORIAL LIGAND STIMULATION, WITH AND WITHOUT IKK-ACTIVATION SYNERGY. 14
APPENDIX FIGURE S12. SINGLE-CELL ANTAGONISM ANALYSIS UNDER COMBINED LPS + PAM STIMULATION. 15
APPENDIX FIGURE S13. CD14 COMPETITION BETWEEN TLR1/2 AND TLR4 SIGNALING PATHWAYS. 16
APPENDIX FIGURE S14. CPG–PIC ANTAGONISM ENHANCES NFκB SIGNALING-CODON SPECIFICITY. 17
APPENDIX FIGURE S15. CHANNEL CAPACITY BETWEEN NFκB SIGNALING CODONS AND LIGAND PRESENCE (CORRESPONDING TO APPENDIX FIGURE S6C) 18
APPENDIX TABLE S1. LIGAND DOSES USED IN THE COMBINATORIAL DOSE-RESOLVED SYNERGY AND ANTAGONISM ANALYSIS. 19

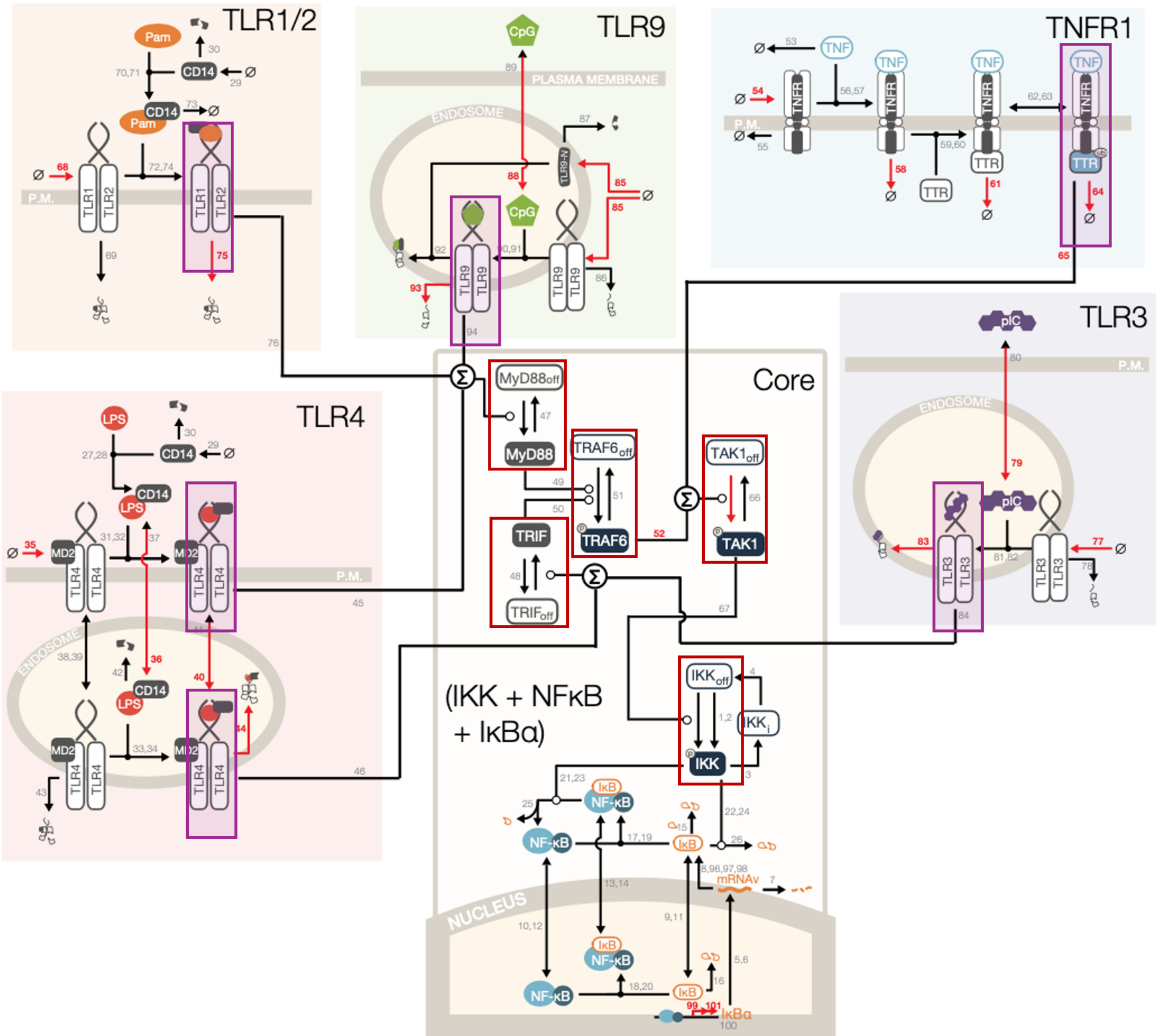

Appendix Figure S1. Schematic of the NFκB signaling network model

The model consists of 5 receptor modules (distinguished by different boxes) and the core module encompassing TAK1, IKK, and NFκB-IκBα feedback. Red arrows and numbers denote the variable parameters for single cells. The purple highlight boxes indicate the generated signaling dynamics from the simulation: nuclear NFκB, IKK, TAK1, TRAF6, TRIF, MyD88, C1 (TNF signaling complex), TLR4LPSen (LPS signaling complex on endosome), TLR4LPS (LPS signaling complex on membrane), TLR9_CpG (CpG signaling complex), TLR3_polyIC (polyIC signaling complex), TLR2_P3CSK (Pam signaling complex) dynamics. Figure is from Guo et al. (2025) which is adapted from Adelaja et al. (2021).

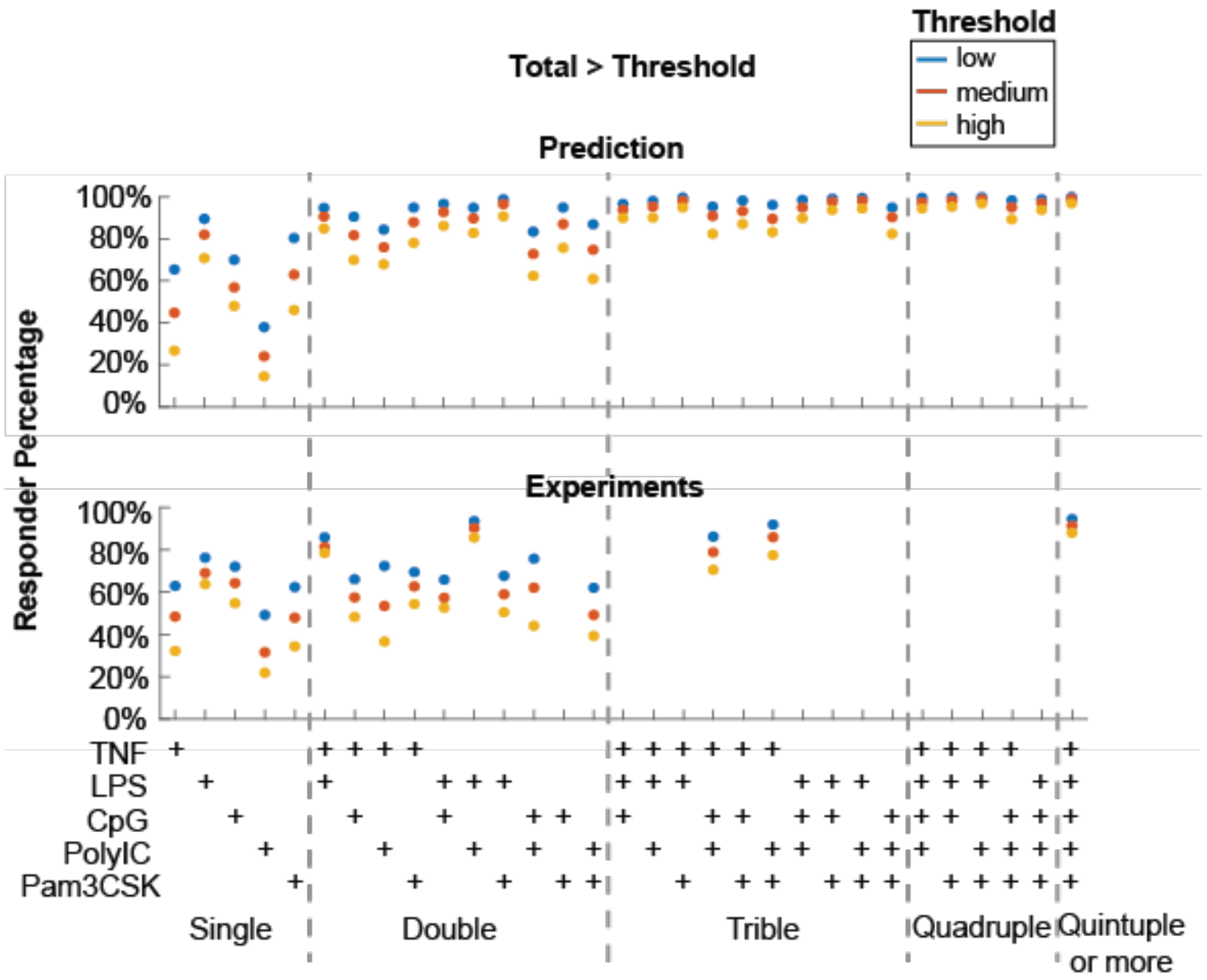

Appendix Figure S2. Responder ratios of NFκB signaling under single- and combinatorial-ligand conditions

Responder fractions defined by a low, medium, or high threshold applied to Total activity for each indicated ligand and ligand combination, for simulation (top) and experimental data (bottom). Thresholds for the simulation data are: thresholds of 0.4 (low), 0.5 (medium), and 0.6 (high) μM·h. Thresholds for the experimental data are: thresholds of 0.2 (low), 0.3 (medium), and 0.4 (high) AU·h. These data define the averages shown in Figure 1B and Figure 1C.

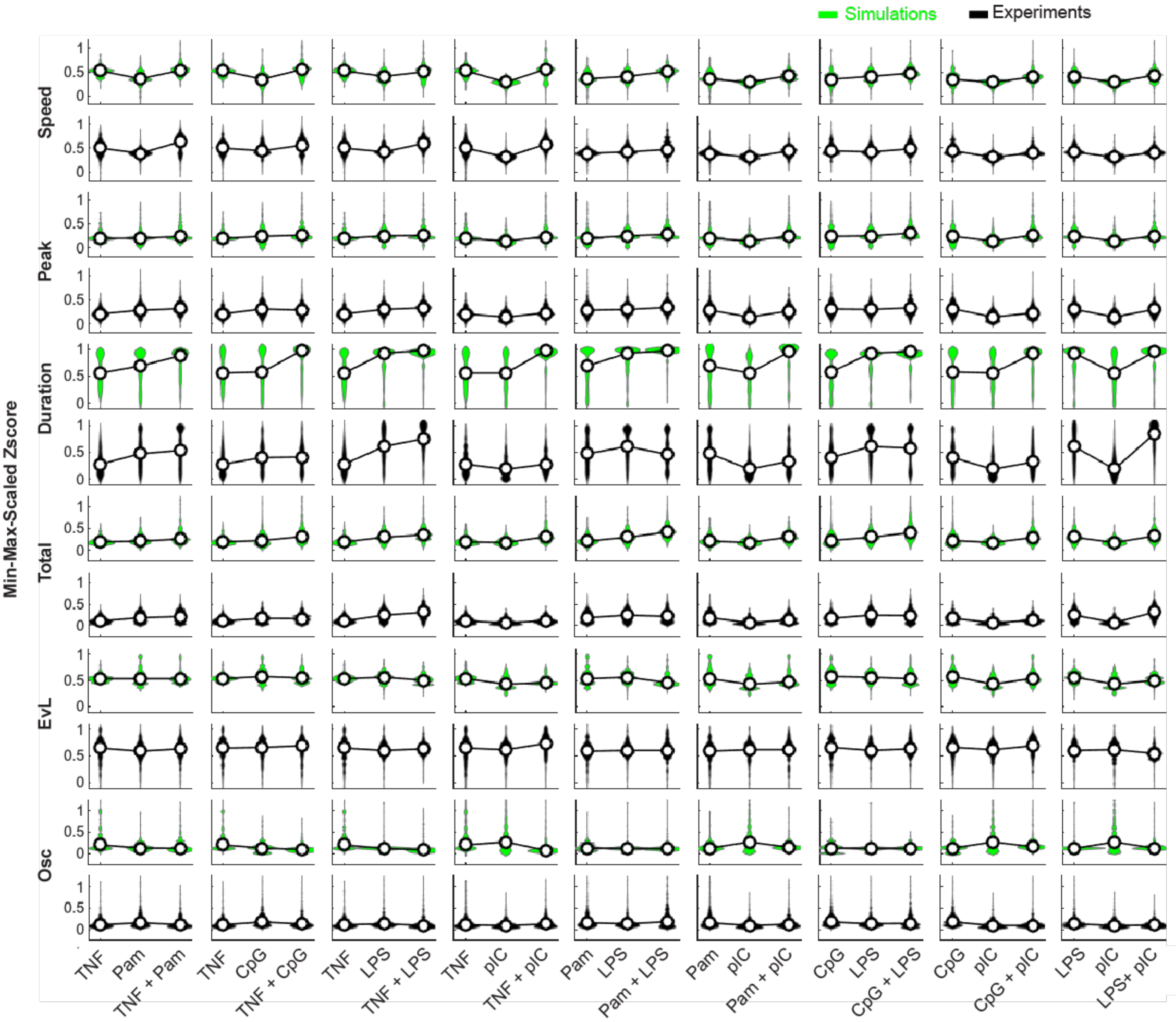

Appendix Figure S3. NFκB signaling codons quantified from model simulations and experimental studies reveal some ligand combination to be integrative and others non-integrative

Violin plots of indicated signaling codon distributions from single and paired ligand stimulations, as calculated from NFκB trajectories predicted by the model (green) or determined experimentally (black). Each pair of rows corresponds to one signaling codon, detailed on the left. Columns correspond to different stimulus conditions. Within each subpanel, the first two columns show the results from single ligand stimulation, and the third column shows the corresponding dual ligand stimulation, with ligand details indicated on the x-axis labels. The hollow circle in each plot symbolizes the median of the respective codon.

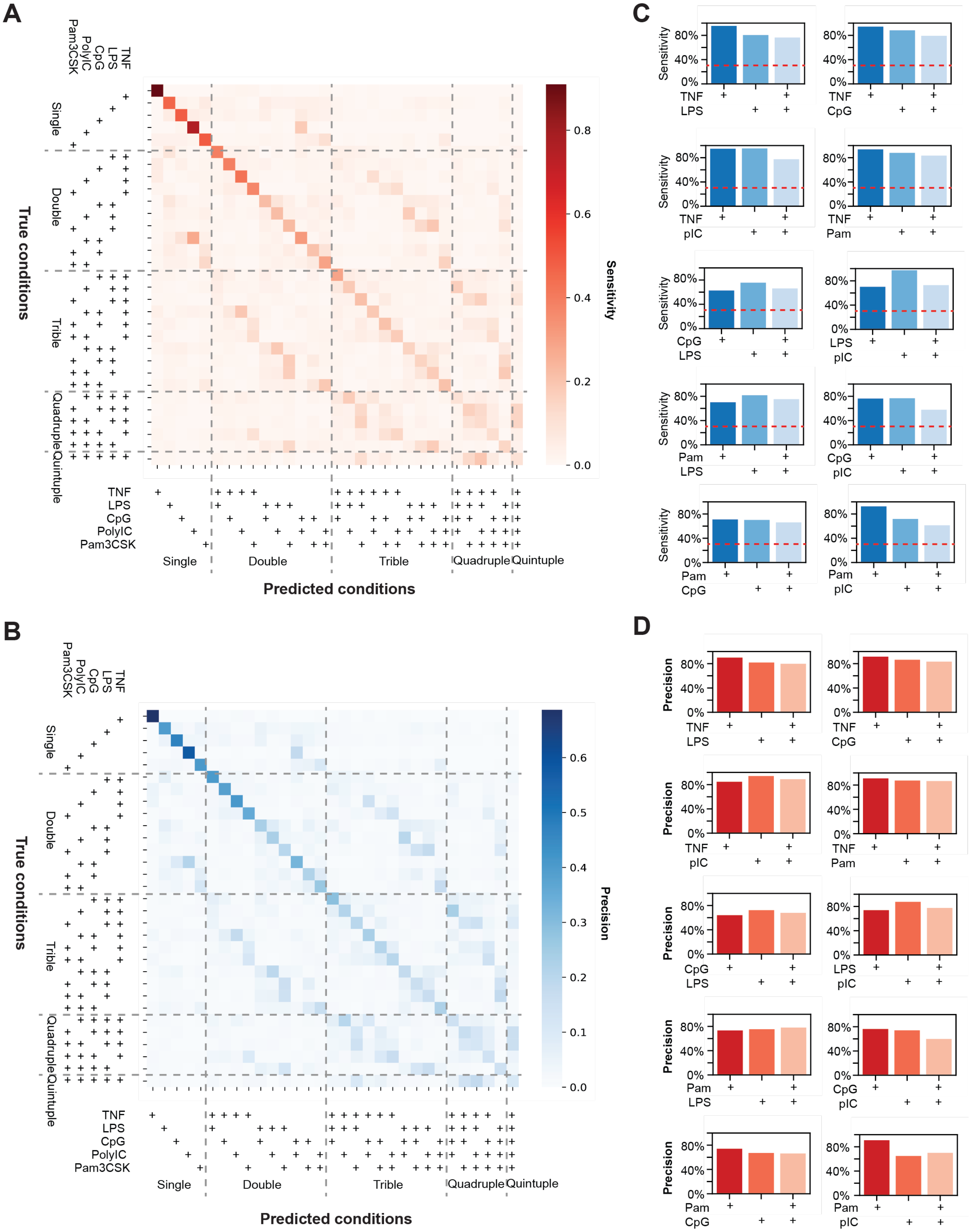

Appendix Figure S4. Classifier predictions of combinatorial ligand responses

A. Heatmap of Sensitivity matrix for the Random Forest classifier (see Methods session "**Stimulus-response specificity (SRS) assessment**"). The classifier was trained on all 31 conditions using NFκB response signaling codons as input to predict the stimulus, with 67% of the data used for training and 33%

for testing. The heatmap shows the results for the test data. The x-axis indicates the predicted conditions, and the y-axis indicates the true conditions. The diagonal elements are also shown in Figure 3C.
B. Heatmap of Precision matrix for the same Random Forest classifier. The diagonal elements are also shown in Figure 3C.
C. Bar plots of Sensitivity of the Random Forest classifiers trained on all 3 conditions (each single ligand and the pair).
D. Bar plots of Precision of the models described in C.

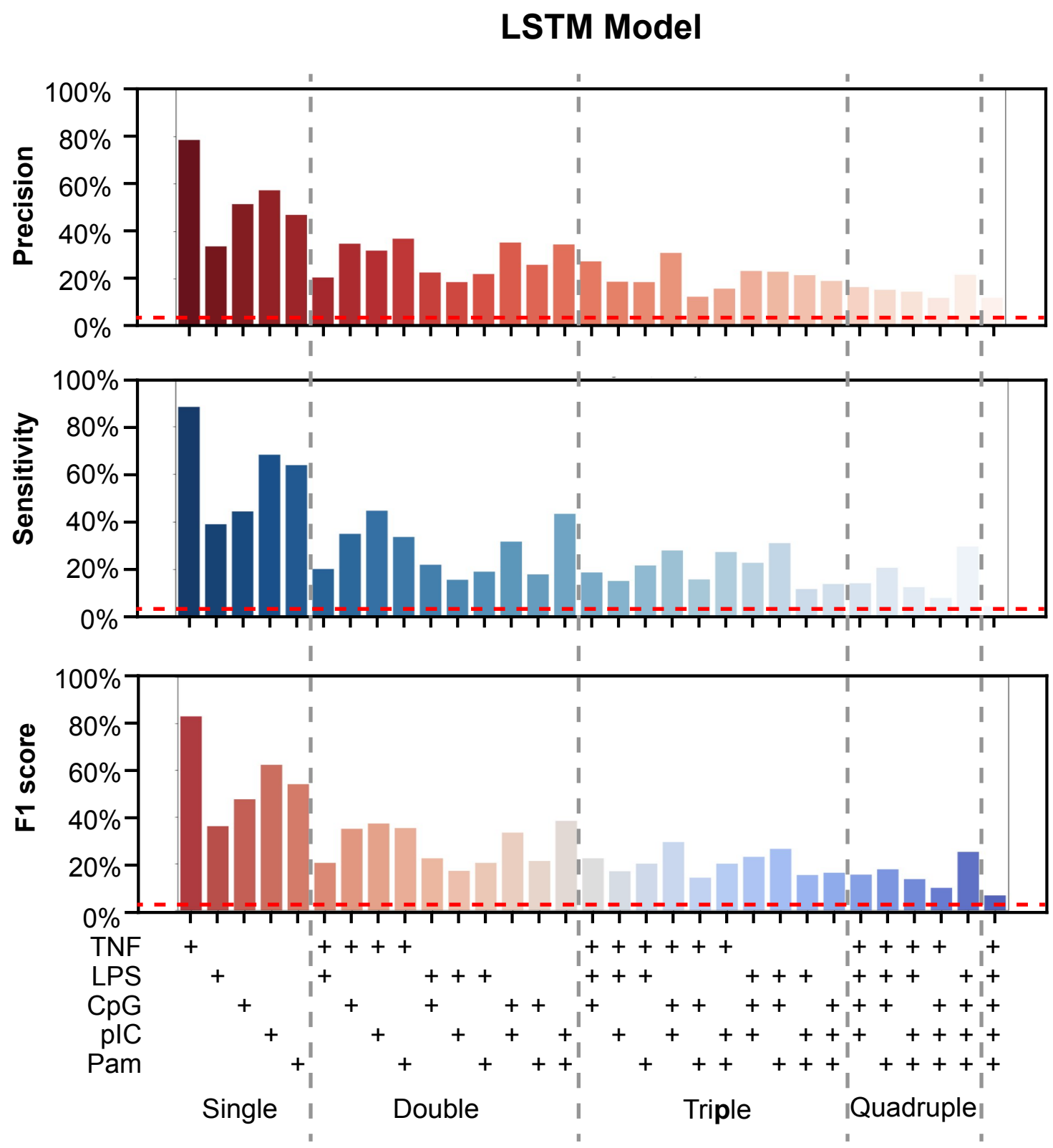

Appendix Figure S5. Predicting stimuli using a Long Short-Term Memory (LSTM) model

Bar plots of Precision, Sensitivity, and F1 score of a Long Short-Term Memory (LSTM) model trained on all 31 conditions using NFκB time-series trajectories as input to predict the stimulus conditions. 60% of the data were used for training, 20% for validation, and 20% for testing. The bar plots are calculated using the 20% validation + 20% testing, in total 40% of the dataset.

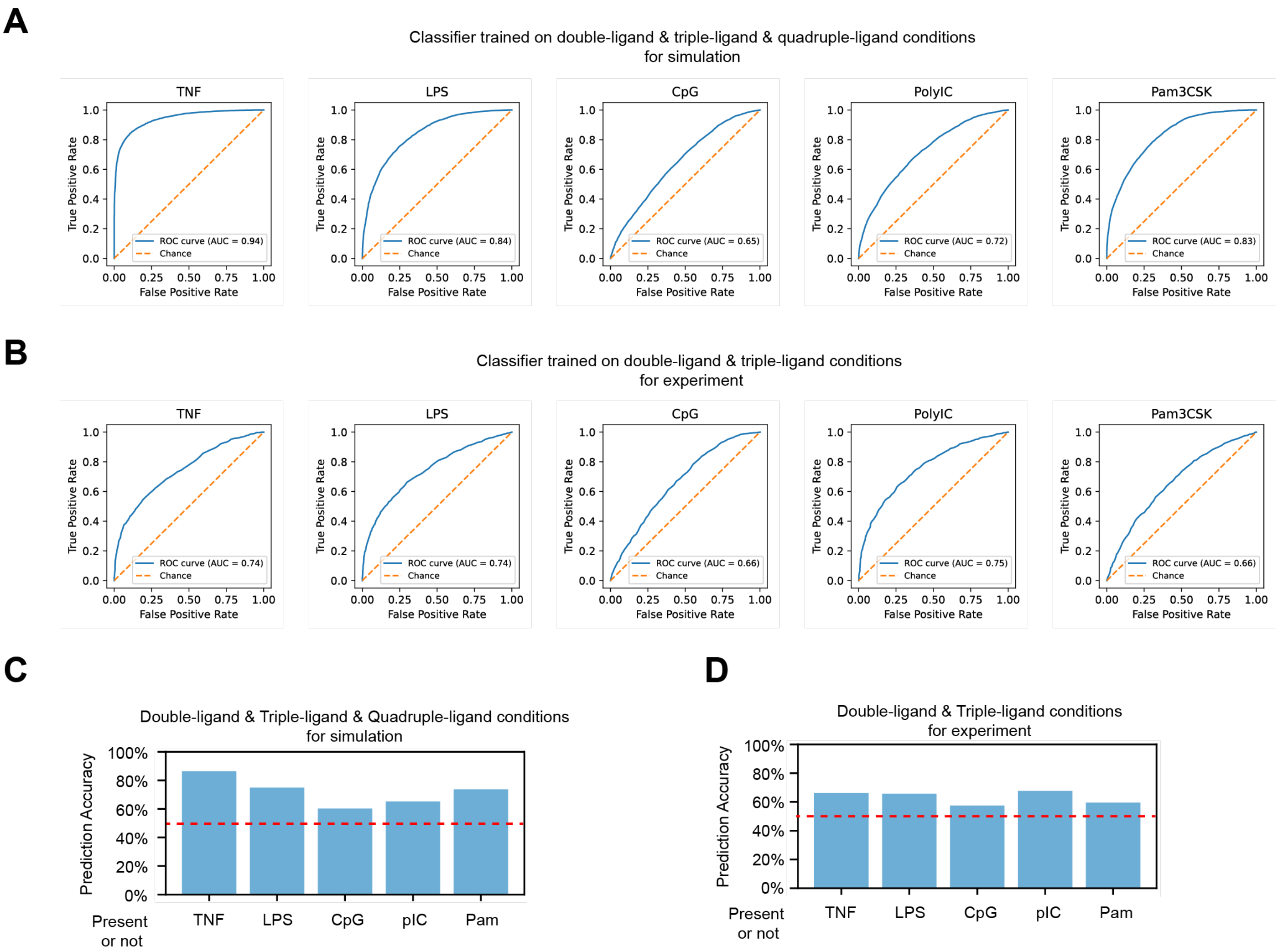

Appendix Figure S6. Classifier predictions of ligand presence under multi-ligand conditions

A-B. ROC curves for classifiers trained to distinguish TNF, LPS, CpG, pIC, and Pam presence versus absence, using all double-, triple-, and quadruple-ligand conditions in simulation data (**A**) or available experimental data (**B**).

C-D. Bar plots of classifier prediction accuracy for the same ligand presence/absence tasks in simulation data (**C**) or experimental data (**D**).

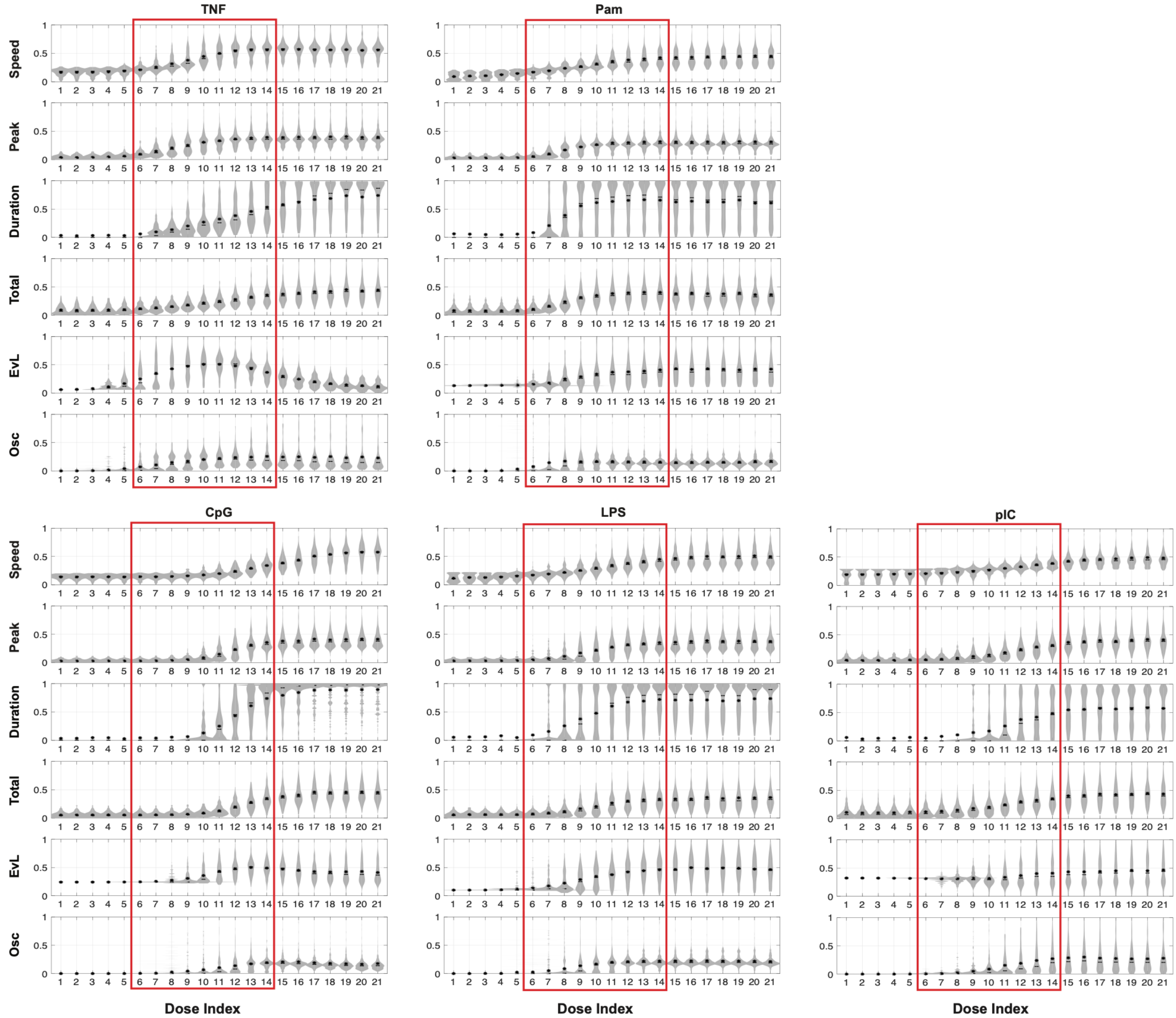

Appendix Figure S7. Selecting functionally relevant doses for each ligand based on NFκB signaling codon distributions.

Violin plots of the distributions of indicated signaling codons (labeled on the left) calculated from NFκB signaling trajectories in response to indicated ligand (labeled in the title) and dose (dose index specified on the x-axis). '*' indicates the mean value, and '-' indicates the median value. The red squares indicate the signaling codon distribution range covered by the selected doses, with D6, D8, D10, D12, and D14 selected for each ligand. The figure is from Guo et al. (2025).

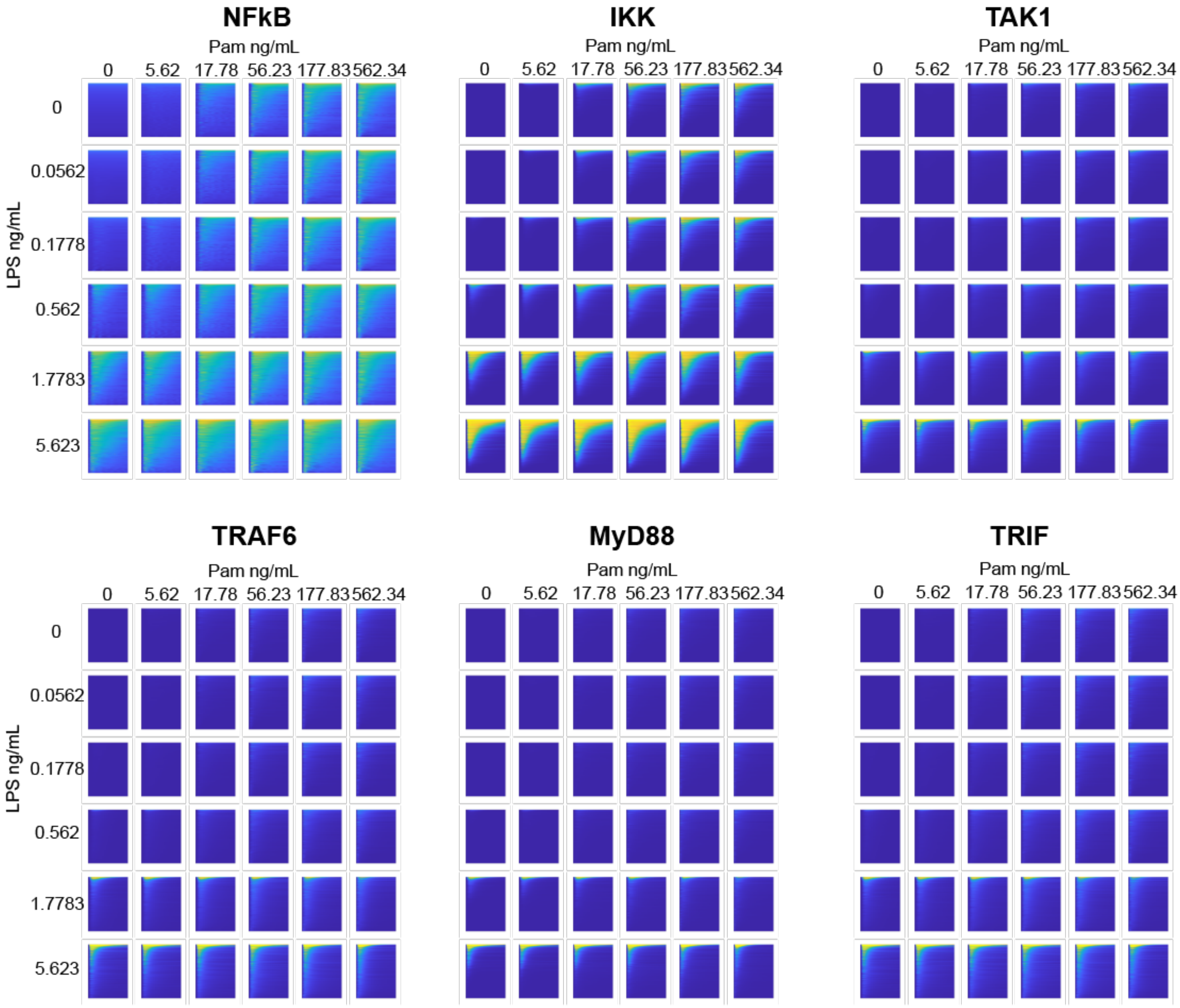

Appendix Figure S8. Exploring the dynamics of key signaling nodes within the NFκB activation pathway.

The heatmaps show simulated single-cell NFκB, IKK, TAK1, TRAF6, MyD88, and TRIF signaling trajectories in response to six doses of LPS (indicated on the left of each group of heatmaps) and Pam (indicated on the top). Within each heatmap, the y-axis denotes individual cells ordered by Total activity, the x-axis denotes time, and color represents the corresponding signaling activity.

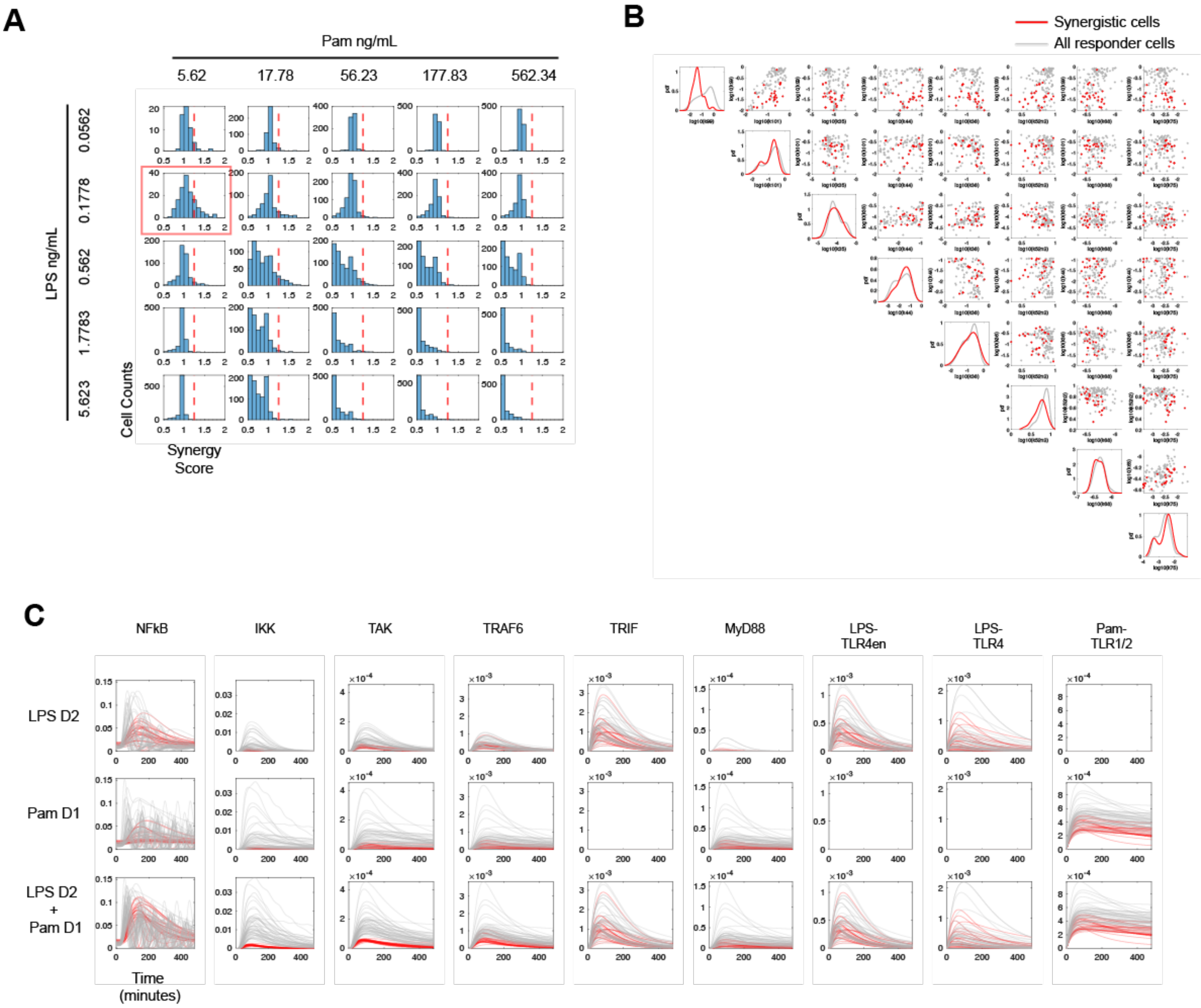

Appendix Figure S9. Single-cell synergy analysis under combined LPS + Pam stimulation

A. Histogram of synergy scores for responsive cells under combined LPS and Pam stimulation at different doses. LPS doses are indicated on the left, and Pam doses are indicated at the top.

B. Distribution curves and scatter plots of model parameters of synergistic cells (red, synergy score > 1.25) and all cells (grey), shown on a log scale. In off-diagonal panels, the parameter IDs are labeled on the x- and y-axes. In diagonal panels, the y-axis shows the probability density function (pdf), the x-axis shows the parameter value, and the biological relevance of each parameter function is illustrated in Figure 4B and detailed in Methods session "**Simulation of heterogenous NFκB dynamical responses**".

C. Single-cell time-series trajectories plot of NFκB, IKK, TAK1, TRAF6, MyD88, TRIF, LPS-TLR4en, LPS-TLR4, Pam-TLR1/2 signaling dynamics (indicated on the top) for synergistic cells (synergy score > 1.25, red lines) and non-synergistic cells (synergy score < 1, grey lines) in response to D2 LPS (top row), D1 Pam (middle row), or their combination (bottom row).

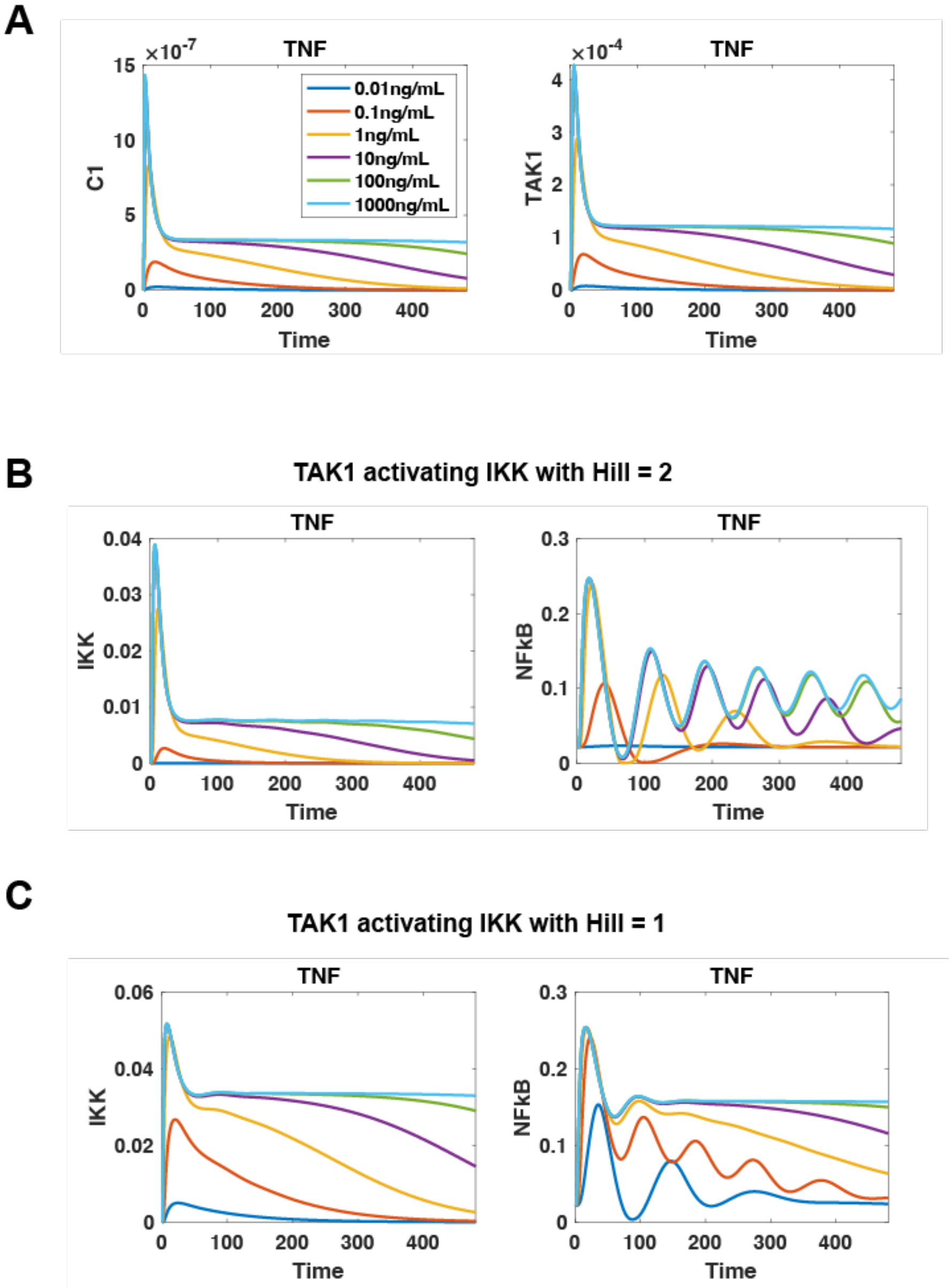

Appendix Figure S10. Representative cell signaling dynamics under different Hill coefficient of TAK1-

mediated IKK activation.

A. Simulated signaling dynamics within the TNFR module across TNF doses. The x-axis is time (up to 480 minutes), and the y-axis is the TNF-signaling complex C1 (left) or TAK1 activity (right). Colors indicate TNF doses (0.01-1000 ng/mL).
B. Simulated signaling dynamics within core module across TNF doses. TAK1-induced IKK activation is modeled with a Hill coefficient = 2. The x-axis is time (up to 480 minutes), and the y-axis is IKK activity (left) or NFκB activity (right). The TNF dose series is indicated by the same color scheme as in A.
C. Same as (B), but with Hill coefficient = 1 for TAK1-induced IKK activation. The TNF dose series is indicated by the same color scheme as in **A**.

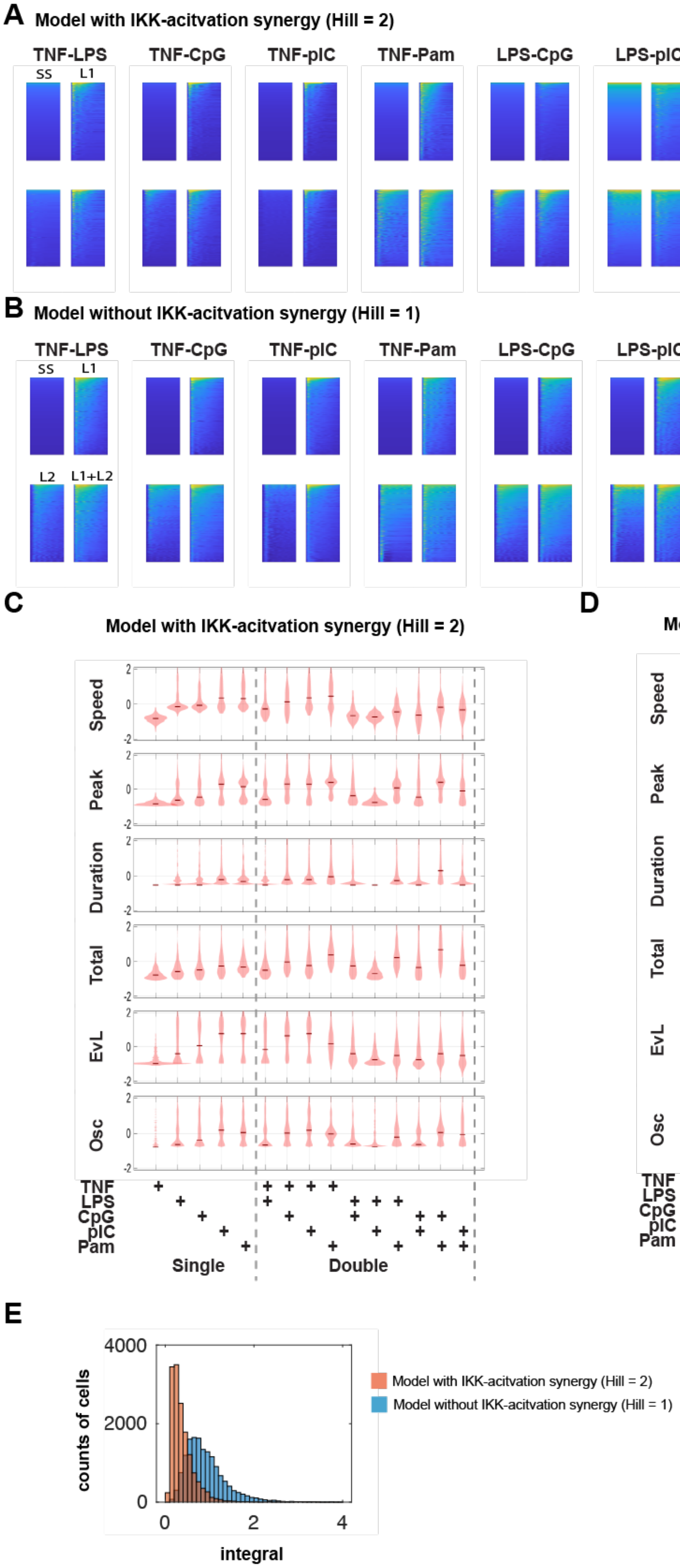

A Model with IKK-acitvation synergy (Hill = 2)
TNF-LPS
TNF-CpG
TNF-pIC
TNF-Pam
LPS-CpG
LPS-pIC
LPS-Pam
CpG-pIC
CpG-Pam
Pam-pIC
SS
L1
B Model without IKK-acitvation synergy (Hill = 1)
L2
L1+L2
C Model with IKK-acitvation synergy (Hill = 2)
D Model without IKK-acitvation synergy (Hill = 1)
Speed
Peak
Duration
Total
EvL
Osc
TNF
LPS
CpG
pIC
Pam
Single
Double
E
counts of cells
integral
Model with IKK-acitvation synergy (Hill = 2)
Model without IKK-acitvation synergy (Hill = 1)

Appendix Figure S11. Single-cell NFκB dynamic profiles under single and combinatorial ligand stimulation, with and without IKK-activation synergy.

A-B. Heatmaps of simulated single-cell NFκB trajectories under single-ligand (L1, L2) and combinatorial ligand (L1+L2) stimulation, using a model with IKK-activation synergy (A, Hill=2) or without (B, Hill = 1). Heatmaps are grouped into 4 for each stimulus pair; conditions within each group are ordered as indicated in the first group. In each subpanel, the x-axis represents time, and color intensity indicates NFκB abundance in accordance with the color bar, and each row represents a single cell prediction, ordered by total activity of the signaling.

C-D. Violin plots of signaling codon distributions from single ligand stimulation to combinatorial ligand stimulation, for model with IKK-activation synergy (C, Hill=2) or without (D, Hill = 1). Conditions are labeled at bottom. The mean value of the respective codon distribution is indicated.

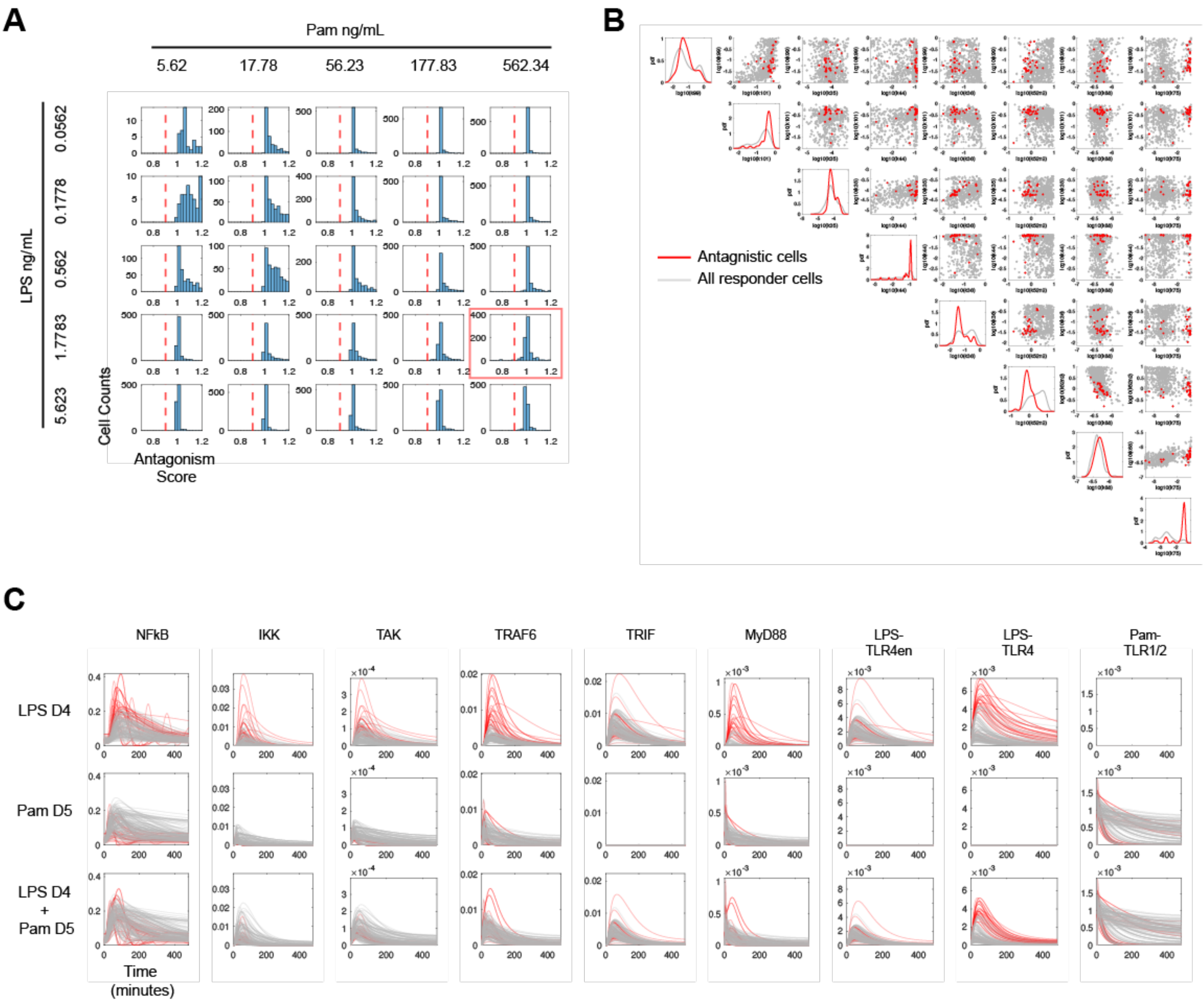

Appendix Figure S12. Single-cell antagonism analysis under combined LPS + Pam stimulation.

A. Histogram of antagonism scores for responsive cells under combined LPS and Pam stimulation at different doses. LPS doses are indicated on the left, and Pam doses are indicated at the top.
B. Distribution curves and scatter plots of parameters for antagonistic cells (curve, antagonism score < 0.9) and all cells (grey), shown in a log scale. In off-diagonal panels, the parameter IDs are labeled on the x- and y-axes, with the log of its numeric values indicated. In diagonal panels, the y-axis shows the probability density function (pdf), the x-axis shows the log of parameter values, and the biological relevance of each parameter function is illustrated in Figure 4B and detailed in Methods session “**Simulation of heterogenous NFκB dynamical responses**”.
C. Single-cell time-series trajectories of NFκB, IKK, TAK1, TRAF6, MyD88, TRIF, LPS-TLR4en, LPS-TLR4, Pam-TLR1/2 signaling dynamics (labeled on the top) for antagonistic cells (antagonism score < 0.9, red lines) and non-antagonistic cells (antagonism score > 1.1, grey lines) in response to D4 LPS (top row), D5 Pam (middle row), or their combination (bottom row).

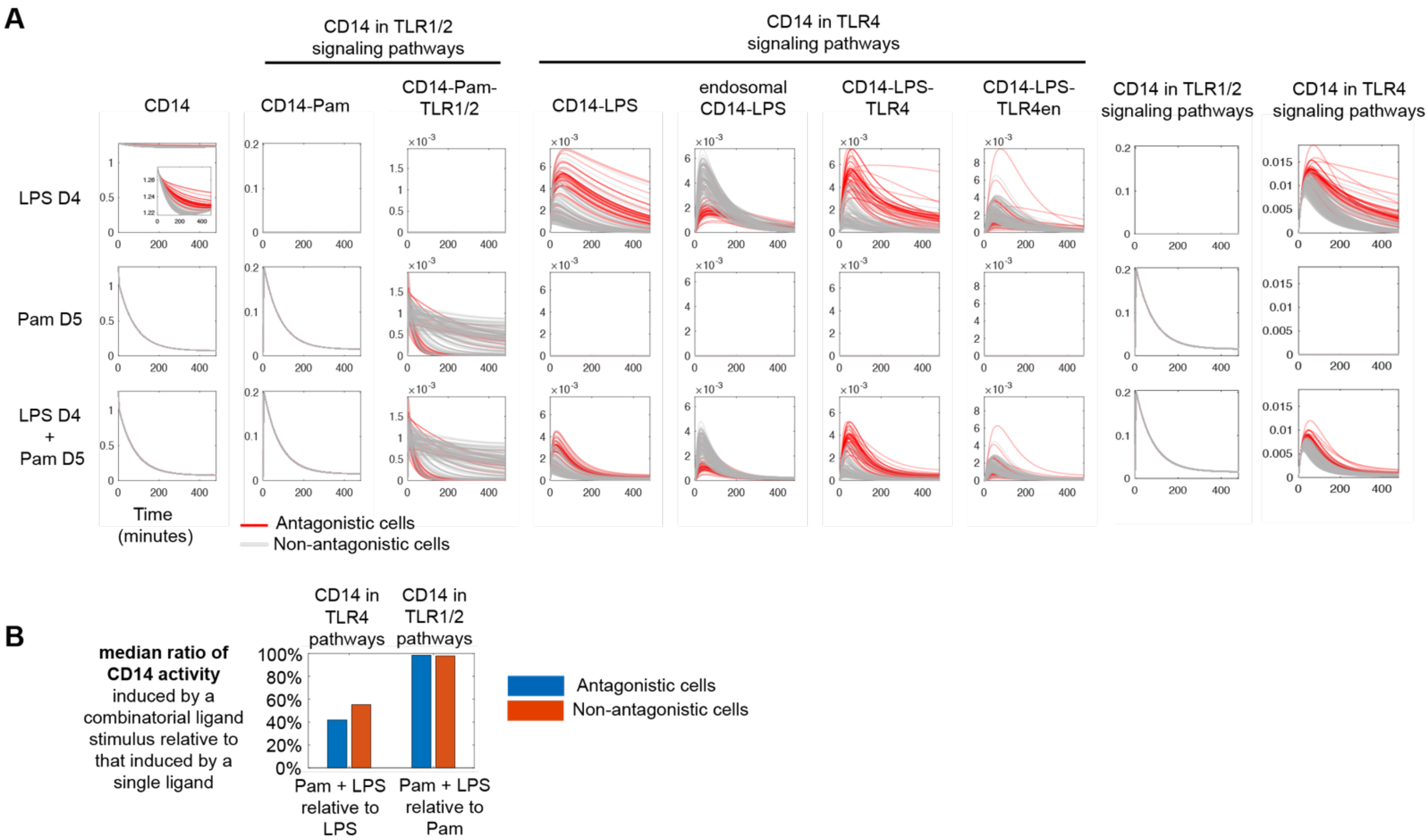

Appendix Figure S13. CD14 competition between TLR1/2 and TLR4 signaling pathways.

A. Single-cell time-series trajectories of free CD14, CD14-Pam, CD14-Pam-TLR1/2, CD14-LPS, endosomal CD14-LPS, CD14-LPS-TLR4, endosomal CD14-LPS-TLR4, total CD14 sequestered by the TLR1/2 pathway, and total CD14 sequestered by the TLR4 pathway signaling dynamics (labeled at the top) for antagonistic cells (antagonism score < 0.9, red lines) and non-antagonistic cells (antagonism score > 1.1, grey lines) in response to D4 LPS (top row), D5 Pam (middle row), or their combination (bottom row). **B.** Bar plots comparing the median ratio of total CD14 allocation in antagonistic and non-antagonistic cells, indicating changes under combined D4 LPS + D5 Pam stimulation relative to the corresponding single-ligand stimulation for the TLR1/2 or TLR4 pathway. For each single cell, the time-integrated total CD14 allocated to the TLR4 pathway was calculated under D4 LPS alone and under combined D4 LPS + D5 Pam stimulation, and the ratio of combined stimulation to LPS alone was computed. The median ratio across antagonistic cells (blue bars) and non-antagonistic cells (red bars) was then plotted respectively. The same calculation was performed for CD14 allocation to the TLR1/2 pathway, using D5 Pam alone as the single-ligand reference. Compared to non-antagonistic cells, antagonistic cells show unchanged or slightly increased CD14 allocation to the TLR1/2 pathway but reduced CD14 allocation to the TLR4 pathway under combined stimulation, suggesting that competition for CD14 limits TLR4 signaling and contributes to LPS–Pam antagonism.

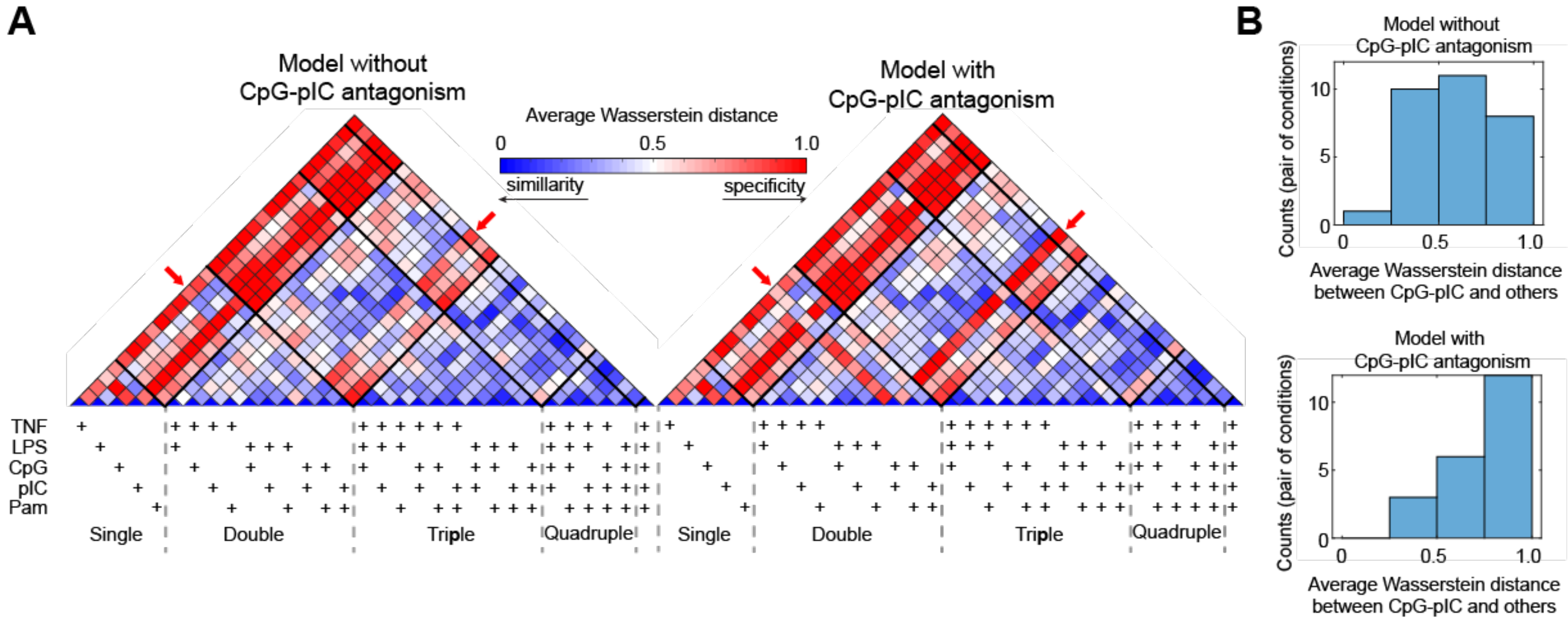

Appendix Figure S14. CpG–pIC antagonism enhances NFκB signaling-codon specificity.

A. Heatmaps of the average Wasserstein distance between the distributions of six NFκB signaling codons for each of 31 conditions (five single-ligand conditions and their combinations, detailed in Figure EV1A-B and Figure EV2A-B) for the original model that does not contain CpG-pIC antagonism and the updated model that does contain CpG-pIC antagonism. Conditions are identified on the diagonal axis. Color intensity indicates average Wasserstein distance in accordance with the color bar.

B. Histogram of the number of single and combinatorial ligand conditions (out of 30 total) whose average Wasserstein distance to the CpG-pIC condition falls within the indicated bin for each of the alternative models (as labeled in the title of each subpanel).

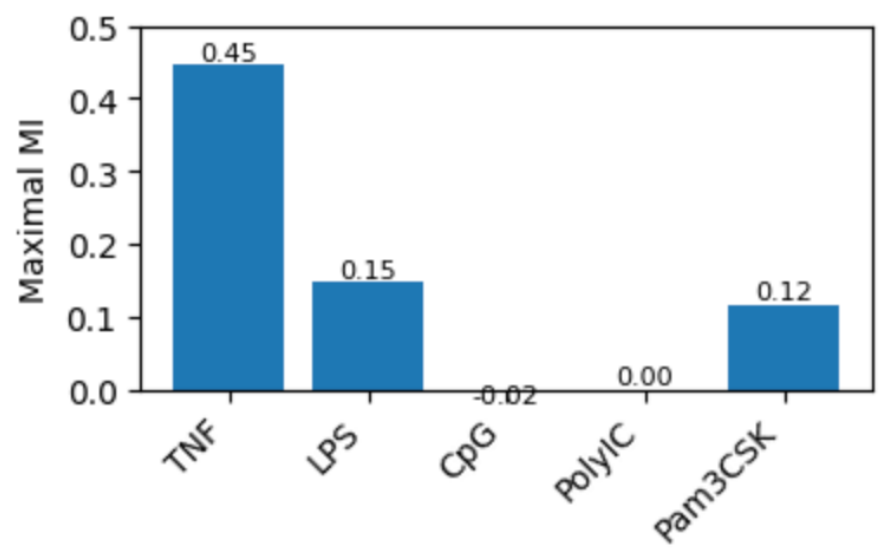

Appendix Figure S15. Channel capacity between NFκB signaling codons and ligand presence (corresponding to Appendix Figure S6C)

Channel capacity, defined as the maximal mutual information across all possible input distributions, was calculated (Ross, 2014) for the binary classification task of determining whether each ligand was present or absent using NFκB signaling codons as input. For each ligand, channel capacity was computed between six NFκB signaling codons and the corresponding binary stimulus label, for example, TNF present versus TNF absent. Bars show the channel capacity for TNF, LPS, CpG, polyIC, and Pam3CSK.

Appendix Table S1. Ligand doses used in the combinatorial dose-resolved synergy and antagonism analysis.

Concentrations (Dose indices D0–D5) applied for each of the five ligands. These dose values are referenced in the Methods ("Single-cell synergy and antagonism score") of the main manuscript.

Dose index	TNF (ng/mL)	LPS (ng/mL)	CpG (nM)	Pam (ng/mL)	pIC (μg/mL)
D0	0	0	0	0	0
D1	0.0562	0.0562	5.62	5.62	0.562
D2	0.178	0.178	17.8	17.8	1.78
D3	0.562	0.562	56.2	56.2	5.62
D4	1.78	1.78	178	178	17.8
D5	5.62	5.62	562	562	56.2
