# Supplementary material for: Macrophage response specificity to ligand mixtures is improved by signaling pathway antagonism
Source: Mol Syst Biol. 2026 Jul 29;22(10):1578–604. doi: 10.1038/s44320-026-00230-9 (PMC13639095; doi:10.1038/s44320-026-00230-9)
Supplement: Supplementary file 3 [file 44320_2026_230_MOESM2_ESM.pdf]

Macrophage response specificity to ligand mixtures is improved by signaling pathway antagonism

Xiaolu Guo, Supriya Sen, Julian Gonzalez, and Alexander Hoffmann

Corresponding author(s): Alexander Hoffmann (ahoffmann@ucla.edu) , Alexander Hoffmann (ahoffmann@ucla.edu)

Review Timeline:

Transfer from Review Commons:	18th Feb 26
Editorial Decision:	24th Mar 26
Revision Received:	1st Jun 26
Editorial Decision:	9th Jun 26
Revision Received:	18th Jun 26
Accepted:	18th Jun 26

Editor: Jingyi Hou

Transaction Report: This manuscript was transferred to Molecular Systems Biology following peer review at Review Commons.

Review #1

1. Evidence, reproducibility and clarity:

Evidence, reproducibility and clarity (Required)

The authors extend an existing mathematical model of NFkB signalling under stimulation of various single receptors, to model that describes responses to stimulation of multiple receptors simultaneously. They compare this model to experimental data derived from live-cell imaging of mouse macrophages, and modify the model to account for potential antagonism between TLR3 and TLR9 response due to competition for endosomal transport. Using this framework they show that, despite distinguishability decreasing with increasing numbers of heterogenous stimuli, macrophages are still able in principle to distinguish these to a statistically significant degree. I congratulate the authors on an interesting approach that extends and validates an existing mathematical model, and also provides valuable information regarding macrophage response.

There are no major issues affecting the scientific conclusions of the paper, however the lack of detail surrounding the mathematical model and the 'signaling codons' that are used throughout the paper make it difficult to read. This is exacerbated by the fact that I was unable to find Ref 25 which apparently describes the model, however I was able to piece together the essential components from the description in Ref 8 and the supplementary material.

Lots of the minor comments below stem from this, however there are also a few other places that could benefit from some additional clarification and explanation.

Significance:

'...it remains unclear complex...' -> '...it remains unclear whether complex...'

Introduction:
'temporal dynamics of NFkB' - it would be good to be more concrete regarding the temporal dynamics of what aspect of this (expression, binding, conformation, etc), if possible.

'signaling codons' - the behaviour of these is key to the entire paper, so even if they are well described in the reference, it would be good to have a short description as early as possible so that the reader can get an idea in their mind what exactly is being discussed here. Later, it would be good to have concrete description of exactly what these capture.

'This challenge…population of macrophages' - this seems a bit out of place, and is a bit of a run on sentence, so I suggest moving this to the next paragraph and working it into the first sentence there '…regulatory mechanisms, and this challenge could be addressed with a model parameterised to account for heterogeneous…Early models …', or something similar.

Ref 25: I can't find a paper with this title anywhere, so if it's an accepted preprint then it would be good to have this available as well. That said, I still think it would be difficult to grasp the work done in this paper without some description of the mathematical model here, at least schematically, if not the full set of ODEs. For example, there are numerous references to how this incorporates heterogeneous responses, the 'core module', etc, and the reader has no context of these if they aren't familiar with the structure of the model.

'A key challenge which is…' -> 'A key challenge is…'

'With model simulation …' -> a bit of a run on sentence, I suggest breaking after 'conditions'.

Results:

This section would benefit from a more in-depth description of the model and experimental setup. In particular for the experiment, the reader never really knows what this workflow for this is, nor what the model ingests as input, and what the predictions are of.

'..mechanistic model was trained…' - trained in this study, or in the previous referenced study?

'determined parameter distributions' - this is where it would be good to have more background on the model. What parameters are these, and what do they correspond to biologically? It would also be nice to see in the methods or supplementary material how this is done (maximum likelihood, etc).

'matching cells with similar core model…' - it's difficult to follow the logic as to why this is done, so I think this needs to be a little clearer. My guess would be that the assumption is that simulated cells with similar 'core' parameters have a similar downstream signalling response, and therefore the receptors can be 'transplanted'. So it would be nice to see exactly what these distributions are and what the effect of a bad match would be.

Some explanation of how this relates to the experimental data the parameters are fit on would also be useful. Is there a correspondence between individual simulated cells and the experimental data for the single ligand stimulation, and then the smallest set of these is taken? Is there also a matching from the simulated multi-receptor modules and the multi-receptor data, and if so, is this done in the same way?

'six signaling codons' - here it would be good to recapitulate what these represent, but also what the 'strength' and 'activity' correspond to (total integrated value, maximum value, etc)

'pre-defined thresholds' - no need to state these numerically in the text (although giving some sense of how/why these were chosen would give some context), but I couldn't find the values of these, nor values corresponding to the signaling codons.

'non-responder cells are likely a result of cellular heterogeneity in receptor modules rather than the core module' - is this the 'ill health' referenced earlier? If so make this clear.

It's also very difficult to follow this chain of logic, given that the reader at this point doesn't have any knowledge of what the 'core' module is, nor the significance of the thresholds on the signaling codons. I would suggest making this much clearer, with reference to each of these.

'...but the model represented these as independent mass action reactions' - the significance of this may not be clear to someone not familiar with biophysical models, so probably better to make it explicit.

'...we trained a random forest classifier...' - is this trained on the 'raw' experimental time series data, or on the signaling codons?

'We also applied a Long Short-Term Memory (LSTM) machine learning model...' - it might be good to reference these three approaches at the beginning of this section, otherwise they seem to come out of the blue a little.

'We then used machine learning classifiers...' - random forests, LSTMs, or a different model?

Discussion:

'...over statistical models...' - suggest maybe 'purely statistical models'

'We found that endosomal transport...' - A paper by Huang, et. al. (https://www.jneurosci.org/content/40/33/6428) observed a synergistic phagocytic response between CpC and pIC stimulation in microglia. This is still consistent with a saturation effect dependent on dose, but may be worth a mention.

'...features termed...' -> 'features, termed'

'...we applied a Long Short-Term Memory (LSTM) machine learning model..' - maybe make clear that this is on the time-series data (also LSTM has already been defined).

Materials and methods:

The descriptions in this section are quite vague, so I would suggest expanding this with more detail from the supplementary material, where things are quite well explained.

'sampling distribution' - not clear what this refers to in this context

'RelA-mVenus mouse strain' - it would be good to mention the relevance of the reporter for NFkB signaling

'...A random forest classifier...' -> a random forest classifier

2. Significance:

Significance (Required)

This study provides mechanistically interpretable insight on the important question of how immune cells perform target recognition in realistic scenarios, and also provides validation of existing mathematical models by extending these beyond their original domain. The paper uses 'signaling codons' as a proxy for information processing, however in this instance it is cross-validated with an LSTM model that is applied directly to the time series data. Nevertheless, the scope of the paper is such that it does not deal with the question of how these signals are transmitted or used in a downstream immune response. To my knowledge, this is the first time that a well established existing mathematical model of signalling response has been extended and applied to heterogeneous ligand mixtures. These results will be of interest to those studying immune cell responses, and to those interested in basic research on mathematical models of signaling and cellular information processing more generally.

My background is in biophysical models, machine learning, and signaling in cancer. I have a basic understanding of immunology, but no experience in experimental cell biology.

3. How much time do you estimate the authors will need to complete the suggested revisions:

Estimated time to Complete Revisions (Required)

(Decision Recommendation)

Between 1 and 3 months

4. *Review Commons* values the work of reviewers and encourages them to get credit for their work. Select 'Yes' below to register your reviewing activity at [Web of Science Reviewer Recognition Service]() (formerly Publons); note that the content of your review will not be visible on Web of Science.

Web of Science Reviewer Recognition

No

Review #2

1. Evidence, reproducibility and clarity:

Evidence, reproducibility and clarity (Required)

Guo et al. developed a heterogeneous, single-cell ODE model of NFκB signaling parameterized on five individual ligands (TNF, Pam, LPS, CpG, pIC) and extended it, via core-module parameter matching, to predict responses to all 31 combinations of up to five ligands. They found that simulated responder fractions and signaling codon features generally agreed with live-cell imaging data . A notable discrepancy emerged for the CpG (TLR9) + pIC (TLR3) pair: experiments exhibited non-integrative antagonism unpredicted by the original model. This issue was resolved by incorporating a Hill-type term for competitive, limited endosomal trafficking of these ligands. Finally, by decomposing NFκB trajectories into six "signaling codons" and applying Wasserstein distances plus random-forest and LSTM classifiers, the authors showed that stimulus-response specificity (SRS) declines with ligand complexity but remains statistically significant even for quintuple mixtures.

This is a well written and scientifically sound manuscript about complexities of cellular signaling, especially considering the limitations of in vitro experiments in recapitulating in vivo dynamics. Here are a few comments and recommendations:

1. The modeling approach used in this manuscript, while interesting, might need further validation. Inferring multi-ligand receptor parameters by matching single-ligand cells on core-module similarity may not capture true co-variation in receptor expression or adaptor availability. Single cell measurements of receptor expressions could be done (e.g. via flow cytometry) to ground this assumption in real data. If the authors think this is out of scope for this manuscript, they could fit core-matched single cell models with two receptor modules from scratch to the two-ligand experimental data. Would this fitted model produce similar receptor parameters compared to the presented approach? At least the authors should add a bit more explanation for why their modeling approach is better (or valid) than fitting the models with 2/3/4/5 receptor modules from scratch to the experimental data.
2. The refined model posits competitive, saturable endosomal transport for CpG and pIC, but no direct measurements of endosomal uptake rates or compartmental saturation thresholds are provided, leaving the Hill parameters under-constrained. The authors could produce dose-response curves for CpG and pIC individually and in combination across a range of concentrations to fit the Hill parameters for competitive uptake. If this is out of scope for this paper, the authors should at least comment on why the endosome hypothesis is better than others e.g. crosstalks and other parallel pathway activations. Especially given that even the refined model simulations with Hill equations for CpG and pIC do not quite match with the experimental data (Fig 2 B,E).
3. Authors asses the distinguishability of single-ligand stimuli and combinatorial ligands stimuli using the simulations from the refined model. While this is informative, the simulated data could propagate deviations from the experimental data to the classifiers. How would the classifiers fare when the experimental data is used to assess the single-stimulus distinguishability? The authors could use the experimental data they already have and confirm their main claim of the paper, that cells retain stimulus-response specificity even with multiple ligand exposure. In short, how would Fig 3E look when trained/validated on available experimental data?
4. While the approach of presented here with multiple simultaneous ligand exposures is a major step towards the in vivo-like conditions, the temporal aspect is still missing. That is, temporal phasing i.e. sequential exposure to multiple ligands as one would expect in vivo rather than all at once. This is probably out of scope for this paper but the authors could comment how how their work could be taken forward in such direction and would the SRS be better or worse in such conditions.

5. There is no caption for Figure 3F in the figure legend nor a reference in the main text.

2. Significance:

Significance (Required)

General assessment: This is a good manuscript in it's present form which could get better with revision. There needs more supporting data and validation to back the main claim presented in the manuscript.

Significance/impact/readership: When revised this manuscript could be of interest to a broad community involving single cells biology, cell and immune signaling, and mathematical modeling. Especially the models presented here could be used a starting point to more complex and detailed modeling approaches.

3. How much time do you estimate the authors will need to complete the suggested revisions:

Estimated time to Complete Revisions (Required)

(Decision Recommendation)

Between 1 and 3 months

4. *Review Commons* values the work of reviewers and encourages them to get credit for their work. Select 'Yes' below to register your reviewing activity at Web of Science Reviewer Recognition Service (formerly Publons); note that the content of your review will not be visible on Web of Science.

Web of Science Reviewer Recognition

Yes

Review #3

1. Evidence, reproducibility and clarity:

Evidence, reproducibility and clarity (Required)

The authors investigate experimentally single macrophages' NF-kB responses to five ligands, separately and to 3 pairs of ligands. Using the single ligand stimulations, they train an existing mathematical model to replicate single-cell NF-kB nuclear trajectories. From what I understand, for each single cell trajectory in response to a given ligand, the best fit parameters of the core module and the receptor module (specific for the given ligand) are found.
Then (again, from what I understand), single ligand models are used to generate responses to combinations of ligands. The parametrizations of single ligand models (to be combined) are chosen to have the most similar core modules. It is not described how the responses to more than one ligand are calculated - I expect that respective receptor modules work in parallel, providing signals to the core module. After observing that the response to CpG+pIC is lower (in terms of duration and total) than for CpG alone, the model is modified to account for competition for endosomal transport required by both ligands.

Having the trained model, simulations of responses to all 31 combinations of ligands are performed, and each NF-κB trajectory is described by six signaling codons-Speed, Peak, Duration, Total, Early vs. Late, and Oscillations. Next, these codons are used to reconstruct (using a random forest model) the stimuli (which may be the combination of ligands). The single and even the two ligand stimuli are relatively well recognized, which is interpreted as the ability of macrophages to distinguish ligands even if present in combination.

Major comments

1. The demonstrated ability to recognize stimuli is based on several key assumptions that can hardly be met in reality.

a) The cell knows the stimulation time, and then it can use speed as a codon. Look on fig. S4A: The trajectories in response to plC are similar to those in response to TNF, but just delayed.

b) The increase of stimulus concentration typically increases Peak, Duration, and Total, so a similar effect can be achieved by changing the ligand or concentration.

c) Distinguishing a given ligand in the presence of some others, even stronger bases, on the assumption that these ligands were given at the same time, which is hardly justified.
2. For single ligands, it would be nice to see how the random forest classifier works on experimental data, not only on in silico data (even if generated by a fitted model).
3. My understanding of ligand discrimination is such that it is rather based on a

combination of pathways triggered than solely on a single transcription factor response trajectory, which varies with ligand concentration and ligand concentration time profile (no reason to assume it is OFF-ON-OFF). For example, some of the considered ligands (plC and CpG) activate IRF3/IRF7 in addition to NF-kB, which leads to IFN production and activation of STATs. This should at least be discussed.

Technical comments

1. Reference 25: X. Guo, A. Adelaja, A. Singh, W. Roy, A. Hoffmann, Modeling single-cell heterogeneity in signaling dynamics of macrophages reveals principles of information transmission. Nature Communications (2025)
does not lead to any paper with the same or a similar title and author list. This Ref is given as a reference to the model. Fortunately, Ref 8 is helpful. Nevertheless, authors should include a schematic of the model.
2. Also Mendeley Data DOI:10.17632/bv957x6frk.1 and GitHub https://github.com/Xiaolu-Guo/Combinatorial_ligand_NFkB lead to nowhere.
3. Dataset 1 is not described. Possibly it contains sets of parameters of receptor modules (different numbers of sets for each module, why?), but the names of parameters never appear in the text, which makes it impossible to reproduce the data.
4. It is difficult to understand how the simulations in response to more than one ligand are performed.

2. Significance:

Significance (Required)

A lot of work has been done, the methodology is interesting, but the biological conclusions are overstated.

3. How much time do you estimate the authors will need to complete the suggested revisions:

Estimated time to Complete Revisions (Required)

(Decision Recommendation)

Between 1 and 3 months

4. *Review Commons* values the work of reviewers and encourages them to get credit for their work. Select 'Yes' below to register your reviewing activity at [Web of Science Reviewer Recognition Service] (formerly Publons); note that the content of your review will not be visible on Web of Science.

Web of Science Reviewer Recognition

Yes

Response to Reviewers

We thank the Reviewers for their appreciative comments (Reviewer 1: "first time that a well-established existing mathematical model of signaling response extended and applied to heterogeneous ligand mixtures") and constructive suggestions for improvement. In this extensive revision, we have not only addressed the suggestions comprehensively but also extended our analysis of signaling antagonism to all doses and at the single-cell level using novel computational workflows. This resulted in the discovery of several mechanisms of antagonism and synergy that are dose-dependent, and dependent on the cell-specific state of the signaling network, thereby manifesting in only a subset of cells.

We have addressed Reviewer comments: we have made substantial revisions to improve clarity, rigor, and biological interpretation. Below we briefly summarize the main concerns raised by Reviewers 1-3 and how we have addressed them.

(1) We have rewritten the Methods section to clarify our approaches. We have also added the explanation of methodology and the rationale in the main text to improve readability and comprehensiveness (Addressing Reviewer #1 comments). This includes explaining and justifying the signaling codon approaches (Reviewer 1), our core-module parameter matching methodology and discussion (Reviewer #1, point 11, Reviewer #2, point 1), and the model schematic (Reviewer #1, point 5).

(2) For one of our major conclusions – that macrophages may distinguish stimuli in the context of ligand mixtures – we have validated these results with experiments, which increases confidence in this conclusion (Reviewer #2, point 3, Reviewer #3, point 2).

(3) We have updated the model for CpG-pIC competition using Michaelis–Menten kinetics without any additional parameters, rather than introducing new free parameters. This change removes parameter freedom for fitting combinatorial conditions, leading to a more constrained and mechanistically grounded model whose predictions align better with experimental data (Updated Figures 2 and S2; Reviewer #2, point 2).

(4) We have addressed all other editorial and clarification-related concerns as well, as detailed in our point-by-point response below.

In addition, we have extended the scope of the manuscript. We have extended our analysis of ligand combinations across a broad dose range, from non-responsive to saturated conditions. This led to several additional discoveries. For example, we show that ultrasensitive IKK activation can underlie synergistic combinations of ligands at low doses. In contrast, beyond the CpG-poly(I:C) antagonism, we identify that competition

for CD14 uptake by LPS and Pam can generate antagonism between these ligands within specific dose ranges.

Importantly, such antagonism or synergy is not evident in all cells in the population. It may also not be picked up by studies of the mean behavior. With our new computational workflow that allows for single-cell resolution we identify the conditions that must be met by the signaling network state, for antagonism or synergy to take place.

Further, we examine the hypothesis that such signaling pathway interactions affect stimulus-response specificity in combinatorial stimulus conditions. By comparing models with and without this antagonism, we demonstrate that antagonistic interactions can improve stimulus-response specificity in complex ligand mixtures.

These additional analyses provide a new mechanistic understanding of cellular information processing and elucidate how synergy and antagonism can mechanistically shape signaling fidelity in response to complex ligand mixtures.

Point-by-Point Response

Reviewer #1

Evidence, reproducibility and clarity

The authors extend an existing mathematical model of NFkB signalling under stimulation of various single receptors, to model that describes responses to stimulation of multiple receptors simultaneously. They compare this model to experimental data derived from live-cell imaging of mouse macrophages, and modify the model to account for potential antagonism between TLR3 and TLR9 response due to competition for endosomal transport. Using this framework they show that, despite distinguishability decreasing with increasing numbers of heterogenous stimuli, macrophages are still able in principle to distinguish these to a statistically significant degree. I congratulate the authors on an interesting approach that extends and validates an existing mathematical model, and also provides valuable information regarding macrophage response.

Response: We thank the reviewer for this appreciative assessment and for the careful reading of our work. The constructive comments helped us substantially improve the rigor and clarity of the manuscript.

In addition to revising the text for clarity, we have extended our analysis to systematically investigate dose-response behavior for each pair of ligand combination. Using the experimentally validated model, we explored 10 ligand pairs across a range of doses from non-responsive to saturating. This allowed us to identify mechanistic regimes in which synergy and antagonism arise at the single-cell level. In particular, we found that low-dose synergy can be explained by ultrasensitive IKK activation (Figure 4 and corresponding supplementary figures), while antagonism can emerge from

competition for shared components such as CD14 (Figure 5 and corresponding supplementary figures). We further show that antagonism can enhance condition distinguishability in ligand mixtures, thereby contributing to stimulus-response specificity (Figure 5 and corresponding supplementary figures).

There are no major issues affecting the scientific conclusions of the paper, however the lack of detail surrounding the mathematical model and the 'signaling codons' that are used throughout the paper make it difficult to read. This is exacerbated by the fact that I was unable to find Ref 25 which apparently describes the model, however I was able to piece together the essential components from the description in Ref 8 and the supplementary material.

Response: This comment helped us to improve the writing. We apologize that the key reference 25 was still not publicly available. It is now published in Nature Communications. In addition, we have added more details to clarify the mathematical model as well as the signaling codons, in results and in methods. Please see below for details.

Lots of the minor comments below stem from this, however there are also a few other places that could benefit from some additional clarification and explanation.

Significance:
1. '...it remains unclear complex...' -> '...it remains unclear whether complex...'
Response: We have rewritten the Significance (now it is Synopsis).

Introduction:
2. 'temporal dynamics of NFkB' - it would be good to be more concrete regarding the temporal dynamics of what aspect of this (expression, binding, conformation, etc), if possible.
Response: It refers to the presence of NFκB into nucleus, which represents active NFκB capable of activating gene expression. We have clarified this (Lines 59-61 in introduction paragraph 2). "*Upon stimulation, NFκB translocates into the nucleus, … activating immune gene expression (10, 15–19).*"

3. 'signaling codons' - the behaviour of these is key to the entire paper, so even if they are well described in the reference, it would be good to have a short description as early as possible so that the reader can get an idea in their mind what exactly is being discussed here. Later, it would be good to have concrete description of exactly what these capture.

Response: We thank the reviewer for this comment. We have added one whole paragraph in the early introduction to describe the concept of Signaling Codons which allow quantitative characterization of NFkB stimulus-response-specific dynamics (Lines 60-67). We have also added more concrete description of Signaling Codons in the results as well as adding an illustration for the signaling codons (Lines 169-175, Figure S2B).

4. 'This challenge...population of macrophages' - this seems a bit out of place, and is a bit of a run on sentence, so I suggest moving this to the next paragraph and working it into the first sentence there '...regulatory mechanisms, and this challenge could be addressed with a model parameterised to account for heterogeneous...Early models ...', or something similar.

Response: We thank the reviewer for this suggestion, we have revised this as suggested. This improves the logic flow (Lines 87-88).

5. Ref 25: I can't find a paper with this title anywhere, so if it's an accepted preprint then it would be good to have this available as well. That said, I still think it would be difficult to grasp the work done in this paper without some description of the mathematical model here, at least schematically, if not the full set of ODEs. For example, there are numerous references to how this incorporates heterogeneous responses, the 'core module', etc, and the reader has no context of these if they aren't familiar with the structure of the model.
Response: We apologize that Ref 25 was not on PubMed. Now it’s published, and we have updated the corresponding information. This comment also helped us to improve the writing by adding a description of the mathematical model in the Introduction (Lines 95-105), the results (Lines 129-141), and a detailed description of the model in the Methods (**Simulation of heterogenous NFκB dynamical responses.**)

We have also added the schematic of the model topology in Figure S1 (adapted from previous publications Guo et al 2025, Adelaja et al 2021) to make sure the paper is self-contained.

6. 'A key challenge which is...' -> 'A key challenge is...'
Response: We have revised the Introduction and removed this sentence.

7. 'With model simulation ...' -> a bit of a run on sentence, I suggest breaking after 'conditions'.
Response: We have revised the introduction and removed this sentence.

Results:

8. This section would benefit from a more in-depth description of the model and experimental setup. In particular for the experiment, the reader never really knows what this workflow for this is, nor what the model ingests as input, and what the predictions are of.

Response: This comment helped us to improve clarity by adding an in-depth description of the model and experimental setup. We have revised the Results as suggested (Lines 129-141). We also appended the corresponding revision here for reviewer reference.

"*This mechanistic model was trained on single-ligand response experimental datasets, capturing the single-ligand stimulus-response specificity of the population of macrophages while accounting for cellular heterogeneity. Specifically, quantitative NFκB dynamic trajectory data from hundreds of single macrophages responding to five single ligands (TNF, pIC, Pam, CpG, LPS) at 3-5 doses was obtained from live cell imaging experiments. The mathematical model (Figure S1) consists of a 52-dimensional system of ordinary differential equations, including 52 intracellular species, 101 reactions and 133 parameters, and is divided into five receptor modules, which respond to the corresponding ligands respectively, and the IKK-NFκB core module that contains the prominent IκBα negative feedback loop. By fitting the single-cell experimental data set with a non-linear mixed effect statistical model (coupling with 52-dimensional NFκB ODE model), the parameter distributions for the single-cell population were inferred. Analyzing the resulting simulated NFκB trajectories with Information theoretic and machine learning classification analyses confirmed that the virtual cell model simulations reproduced key SRS performance characteristics of live macrophages.*"

9. '..mechanistic model was trained...' - trained in this study, or in the previous referenced study?

Response: The mechanistic model was trained in a previous study (Guo et al 2025 Nature Comm), and we have clarified this in the revision (Lines 127 - 129).

10. 'determined parameter distributions' - this is where it would be good to have more background on the model. What parameters are these, and what do they correspond to biologically? It would also be nice to see in the methods or supplementary material how this is done (maximum likelihood, etc).

Response: This comment helps us to clarify the predetermined parameter distributions. We have revised the methods to include this information (***Simulation of heterogenous***

NFκB dynamical responses, paragraph 3). We have appended the corresponding text here for reviewer's convenience.

"The ODE model was then fitted to the population of single-cell trajectories to recapitulate the cell-to-cell heterogeneity in the experimental data (2). This is achieved by solving the non-linear mixed effects model (NLME) through stochastic approximation of expectation maximation algorithm (SAEM) (3–6). Seventeen parameters were estimated. Within the core module, the estimated parameters included the rates governing TAK1 activation (k52, k65), the time delays of IκBα transcription regulated by NFκB (k99, k101), and the total cellular NFκB abundance (tot NFκB). Within the receptor module, receptor synthesis rates (k54 for TNF, k68 for Pam, k85 for CpG, k35 for LPS, k77 for pIC), degradation rates of the receptor–ligand complexes (k56, k61, k64 for TNF; k75 for Pam; k93 for CpG; k44 for LPS; k83 for pIC), and endosomal uptake rates (k87 for CpG; k36 and k40 for LPS; k79 for pIC) were fitted. All remaining parameters were fixed at literature-suggested values (1). The single-cell parameters inferred from experimental individual-cell trajectories then served as empirical distributions for generating the new dataset (see SupplementaryDataset2)."

11. 'matching cells with similar core model...' - it's difficult to follow the logic as to why this is done, so I think this needs to be a little clearer. My guess would be that the assumption is that simulated cells with similar 'core' parameters have a similar downstream signalling response, and therefore the receptors can be 'transplanted'. So it would be nice to see exactly what these distributions are and what the effect of a bad match would be.

Response: We thank the reviewer for this comment. In the revision, we have explained the rationale for matching cells with similar core module (Lines 145-152).

"*Previous work determined parameter distributions for only the cognate receptor module (and the core module) that provided the best fit for the relevant single ligand experimental data (Figure 1A, Step 1), but other receptor modules' parameter values were not determined. To simulate stimulus responses to more than two ligands, we imputed the other ligand-receptor module parameters using shared core-module parameters as common variables and employing nearest-neighbor hot-deck imputation (35). In this setup, the core module functions as an "anchor" to harmonize two or more receptor-specific parameter distributions.*"

This nearest-neighbor hot-deck imputation approach (the core module matching method) was shown to outperform other approaches, including random matching and rescaled-similarity matching (Guo et al. 2025, Supplementary Figure S11). For the reviewer's convenience, we have also appended the corresponding figure below.

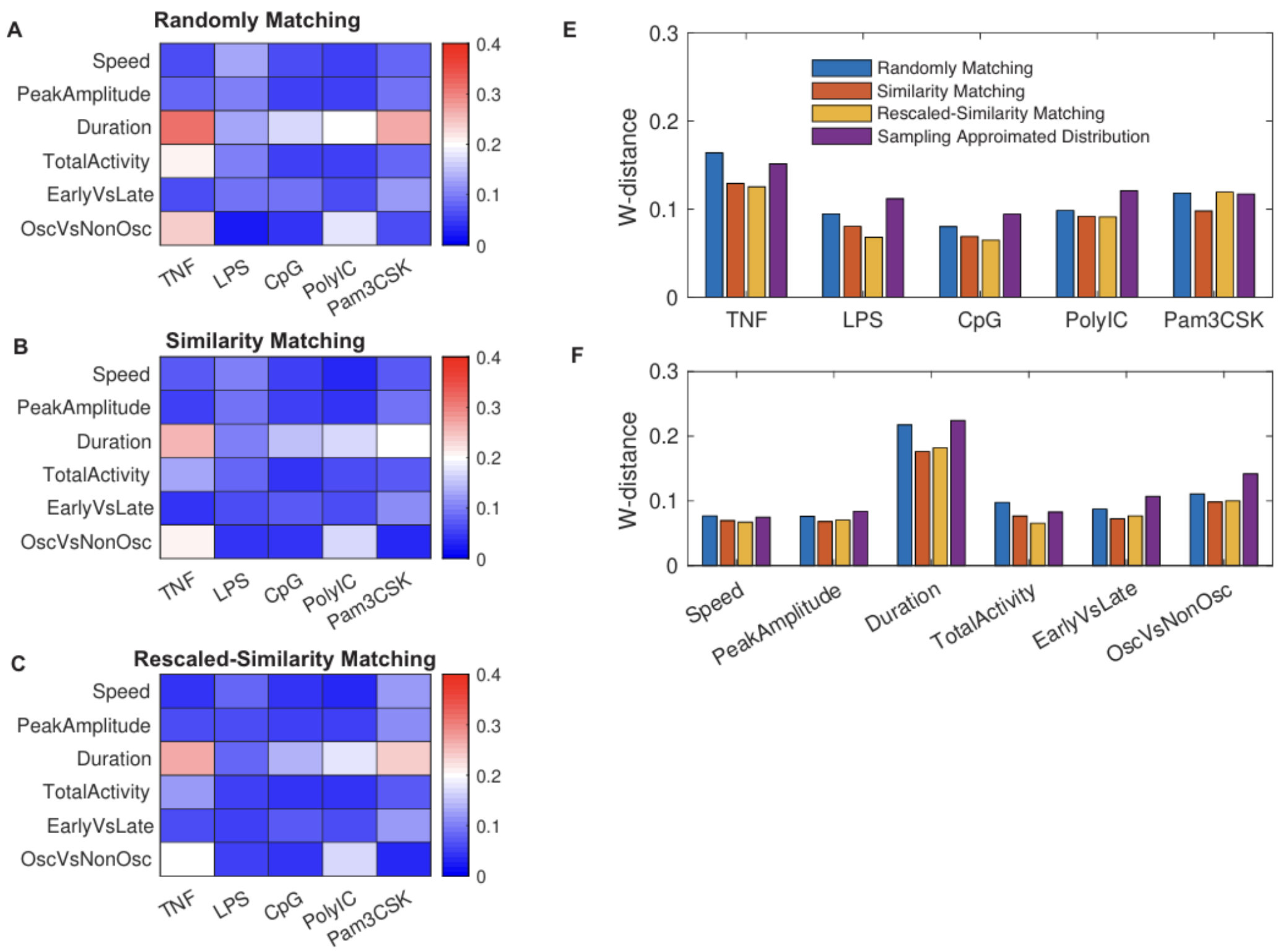

Figure S11 from (Guo et al., 2025). Assessment of matching techniques for predicting single-cell responses to various ligand stimuli (a-d). Heatmaps illustrating the Wasserstein distance between the signaling codon distributions predicted by the model and those observed in experiments. The analysis employs four distinct matching methods to align the five ligand-receptor module parameters: (a) "Random Matching", (b) "Similarity Matching" (the method used in our study), (c) "Rescaled-Similarity Matching", and (d) "Sampling Approximated Distribution". In the heatmaps, rows represent signaling codons, columns denote ligands, and the color intensity indicates the Wasserstein distance, providing a visual metric of similarity between model predictions and experimental data. e-f. Histogram of the average Wasserstein distance between the model-predicted and experimentally observed signaling codon distributions, summarized across signaling codons (e) and ligands (f).

12. Some explanation of how this relates to the experimental data the parameters are fit on would also be useful. (a) Is there a correspondence between individual simulated cells and the experimental data for the single ligand stimulation, and then the smallest set of these is taken? Is there also a matching from the simulated multi-receptor modules and the multi-receptor data, and if so, is this done in the same way?
Response: This comment to help us clarify the correspondence relationship between model simulations and experimental data.

Yes—there is a correspondence between individual simulated cells and the previously published experimental data (Guo et al., 2025b) for single-ligand stimulation. We have revised the first paragraph of the Results (Lines 136–148) and the Methods (Lines 544-557) to clarify how the model simulations were fit to the previous experimental dataset. See Reviewer 1, Comments 10 for the updates in Methods. We have pasted in the revised Results section below for the reviewer's reference.

"*By fitting the single-cell experimental data set with a non-linear mixed effect statistical model (coupling with 52-dimensional NFκB ODE model), the parameter distributions for the single cell population were inferred.*"

13. 'six signaling codons' - here it would be good to recapitulate what these represent, but also what the 'strength' and 'activity' correspond to (total integrated value, maximum value, etc)
Response: We thank the reviewer for the suggestion and have clarified this point (Lines 169-175, Figure S2B).

14. 'pre-defined thresholds' - no need to state these numerically in the text (although giving some sense of how/why these were chosen would give some context), but I couldn't find the values of these, nor values corresponding to the signaling codons.
Response: We appreciate the reviewer's comment. We have added this information in the figure legend (Figure 1B-C) and Method -- "Responder fraction" (Lines 666-672). Specifically, for the model simulation data, the integral thresholds are 0.4 (μM·h), 0.5 (μM·h), and 0.6 (μM·h). The peak thresholds are 0.12 (μM), 0.14 (μM), and 0.16 (μM). For the experimental data, the integral thresholds are 0.2 (A.U.·h), 0.3 (A.U.·h), and 0.4 (A.U.·h). The peak thresholds are 0.14 (A.U.), 0.18 (A.U.), and 0.22 (A.U.). Thresholds were selected so that the medium threshold yields 50% responder cells under single-ligand conditions, while the responder ratio remains unsaturated under three-ligand stimulation.

15. 'non-responder cells are likely a result of cellular heterogeneity in receptor modules rather than the core module' - is this the 'ill health' referenced earlier? If so make this clear.
Response: Yes, this is the 'ill health' referenced earlier, and we have clarified this (Lines 198-199).

16. It's also very difficult to follow this chain of logic, given that the reader at this point doesn't have any knowledge of what the 'core' module is, nor the significance of the thresholds on the signaling codons. I would suggest making this much clearer, with reference to each of these.
Response: We apologize for the poor explanation. We have now explained in the Introduction (Lines 95-106) and the results (Lines 129-141) how the model is structured into receptor-proximal modules that converge on the common core module. We have also added a schematic for clarity (Figure S1). For further clarification of the math models, we have significantly revised the Methods (***Simulation of heterogenous NFκB***

dynamical responses). The defined thresholds are clarified in the Methods -- "***Responder fraction***".

17. '...but the model represented these as independent mass action reactions' - the significance of this may not be clear to someone not familiar with biophysical models, so probably better to make it explicit.
Response: We thank the reviewer for this reminder, and we have added a description of the significance of this point (Lines 225-227).

18. '...we trained a random forest classifier...' - is this trained on the 'raw' experimental time series data, or on the signaling codons?
Response: It is trained on the signaling codons calculated from model simulations of NFκB trajectories. We have clarified this (Lines 260-261).

19. 'We also applied a Long Short-Term Memory (LSTM) machine learning model...' - it might be good to reference these three approaches at the beginning of this section, otherwise they seem to come out of the blue a little.
Response: We have added the references of these three approaches in the beginning of this section (Lines 242-246).

20. 'We then used machine learning classifiers...' - random forests, LSTMs, or a different model?
Response: We have clarified that this as random forest classifier (Line 276).

Discussion:

21. '...over statistical models...' - suggest maybe 'purely statistical models'
Response: We thank the reviewer for this suggestion. We have rewritten the whole Discussion to include the new insights of antagonism and synergy and their roles in maintaining unexpectedly high SRS performance. Thus, this sentence was removed.

22. 'We found that endosomal transport...' - A paper by Huang, et. al. (https://www.jneurosci.org/content/40/33/6428) observed a synergistic phagocytic response between CpC and pIC stimulation in microglia. This is still consistent with a saturation effect dependent on dose, but may be worth a mention.
Response: We thank the reviewer for referring this interesting paper to us, and this comment helps us to improve the Discussion of inflammatory signaling pathways

besides NFκB. This paper demonstrates synergistic effects between CpG and pIC in inhibiting tumor growth and promoting cytokine production (Huang et al., 2020), such as IFN-β and TNF-α, whose expression is also regulated by the IRF and MAPK signaling pathways (Luecke et al., 2021; Sheu et al., 2023). This finding does not contradict our findings that CpG and pIC act antagonistically in the NFκB signaling pathway because of the combinatorial pathways that act on gene expression: CpG can activate the MAPK signaling pathway (Luecke et al., 2024) but not the IRF signaling pathway, whereas pIC activates the IRF signaling pathway (Akira and Takeda, 2004) but only weakly the MAPK pathway. Therefore, their combination can synergistically regulate inflammatory responses. We have added this to the discussion (Lines 515-522).

23. '...features termed...' -> 'features, termed'
Response: We thank the reviewer for their carefully reading, and we have rewritten the Discussion.

24. '...we applied a Long Short-Term Memory (LSTM) machine learning model..' - maybe make clear that this is on the time-series data (also LSTM has already been defined).
Response: We thank the reviewer for their carefully reading, and we have rewritten the Discussion.

Materials and methods:

25. The descriptions in this section are quite vague, so I would suggest expanding this with more detail from the supplementary material, where things are quite well explained.
Response: We thank the reviewer for this suggestion, and we have rewritten the whole Methods as suggested.

26. 'sampling distribution' - not clear what this refers to in this context
Response: We have clarified this in the revision (Methods -- ***Simulation of heterogenous NFκB dynamical responses***, paragraph 3).
"The single-cell signaling-pathway parameter values used for bootstrapping sampling to generate model simulations are given in Supplementary dataset 2."

27. 'RelA-mVenus mouse strain' - it would be good to mention the relevance of the reporter for NFkB signaling
Response: We have added the relevance of the reporter for NFkB signaling (Methods, Lines 624-626).

28. '...A random forest classifier...' -> a random forest classifier

Response: We have rewritten the methods.

Significance

29. This study provides mechanistically interpretable insight on the important question of how immune cells perform target recognition in realistic scenarios, and also provides validation of existing mathematical models by extending these beyond their original domain. The paper uses 'signaling codons' as a proxy for information processing, however in this instance it is cross-validated with an LSTM model that is applied directly to the time series data. Nevertheless, the scope of the paper is such that it does not deal with the question of how these signals are transmitted or used in a downstream immune response. To my knowledge, this is the first time that a well established existing mathematical model of signalling response has been extended and applied to heterogeneous ligand mixtures. These results will be of interest to those studying immune cell responses, and to those interested in basic research on mathematical models of signaling and cellular information processing more generally.

My background is in biophysical models, machine learning, and signaling in cancer. I have a basic understanding of immunology, but no experience in experimental cell biology.

Response: We thank the reviewer for highlighting the novelty of our study. We appreciate the reviewer's recognition that our work advances the understanding of cellular information processing in the context of ligand mixtures, particularly as the first to extend computational models to investigate signaling fidelity under mixed-ligand conditions.

We agree that this work will interest computational biologists focused on signaling network modeling and information processing. In addition, we believe it will also be valuable for all signaling biologists, as we provide fundamental insights. For experimental biologists in particular, our model provides an efficient, quantitative framework for exploring and generating testable hypotheses.

We would also like to gently emphasize that evaluating specificity within signaling pathways is as essential as studying downstream functional responses. While immune function outcomes are certainly important, they rely on the upstream signaling pathways that first respond to environmental cues. Understanding how these signaling pathways achieve specificity and discriminability is therefore crucial. For example, this is particularly relevant for drug development targeting pathways such as NFκB, where

assessing the direct signaling output—NFκB activation dynamics—can provide valuable insight into the effects of pharmacological interventions.

Reviewer #2

Evidence, reproducibility and clarity

Guo et al. developed a heterogeneous, single-cell ODE model of NFκB signaling parameterized on five individual ligands (TNF, Pam, LPS, CpG, pIC) and extended it, via core-module parameter matching, to predict responses to all 31 combinations of up to five ligands. They found that simulated responder fractions and signaling codon features generally agreed with live-cell imaging data. A notable discrepancy emerged for the CpG (TLR9) + pIC (TLR3) pair: experiments exhibited non-integrative antagonism unpredicted by the original model. This issue was resolved by incorporating a Hill-type term for competitive, limited endosomal trafficking of these ligands. Finally, by decomposing NFκB trajectories into six "signaling codons" and applying Wasserstein distances plus random-forest and LSTM classifiers, the authors showed that stimulus-response specificity (SRS) declines with ligand complexity but remains statistically significant even for quintuple mixtures.
This is a well written and scientifically sound manuscript about complexities of cellular signaling, especially considering the limitations of in vitro experiments in recapitulating in vivo dynamics.

Response: We thank the reviewer for carefully reading the manuscript and for this endorsement. We have significantly improved the manuscript thanks to the reviewer's insightful comments (see below for point-to-point responses).

Besides addressing the reviewer's questions, we have further extended our work to investigate how ligand pairs interact across all doses and how those interactions affect stimulus-response specificity. As the reviewer pointed out, experimental studies are limited in recapitulating the multitude of complex physiological contexts. The model is helpful to explore more complex scenarios beyond the feasibility of in-vitro experimental setups. Using computational simulations, we have further explored 360 conditions generated from 10 ligand pairs, each evaluated at 6 doses spanning non-responsive to saturating levels, and with each condition considered 1000 cells to capture the heterogeneity of the population.

From this extended analysis, we identified the mechanistic bases for observations of both synergy and antagonism. Synergy for certain low-dose ligand combinations can be explained by ultrasensitive IKK activation (Figure 4), while antagonism between LPS and Pam arises from competition for the cofactor CD14 (Figure 5). We show that these phenomena are dependent on the signaling network state and therefore are not

observed in all cells of the population. We define the network conditions that must be met for antagonism and synergy to occur. Importantly, we then show that antagonism can contribute to stimulus-response specificity in ligand mixtures (Figure 5).

Here are a few comments and recommendations:

1. The modeling approach used in this manuscript, while interesting, might need further validation. Inferring multi-ligand receptor parameters by matching single-ligand cells on core-module similarity may not capture true co-variation in receptor expression or adaptor availability. Single cell measurements of receptor expressions could be done (e.g. via flow cytometry) to ground this assumption in real data. If the authors think this is out of scope for this manuscript, they could fit core-matched single cell models with two receptor modules from scratch to the two-ligand experimental data. Would this fitted model produce similar receptor parameters compared to the presented approach? At least the authors should add a bit more explanation for why their modeling approach is better (or valid) than fitting the models with 2/3/4/5 receptor modules from scratch to the experimental data.

Response: We thank the reviewer for this comment, this helped us improve the explanation of the methodology, the rationale, and the validation. The methodology is based on the well-established statistical method of nearest-neighbor hot-deck imputation (Andridge and Little, 2010). In this implementation, the core module functions as a stabilizing "anchor" (common variables) to harmonize various receptor-specific parameter distributions. Similar methodologies have been successfully applied to correct batch effects or integrate single-cell RNAseq datasets using anchor cell types (Stuart et al., 2019). Our workflow has been validated on single-ligand stimuli conditions in a previous study (Guo et al., 2025) (See below 3rd paragraph). Here, we used this method to generate predictions for ligand mixtures and have validated them with experimental studies of the dual-ligand stimuli, and we found that our predictions align well with the experimental data. As the reviewer suggested in point 3, in the revision, we also added experimental validation on the binary classifiers of macrophage determines whether specific stimuli are presented in the ligand mixture. The question we are interested in in this work is how macrophage process ligand-specific information in the context of ligand mixtures. For this question, the experimental results align with the model predictions, reaching consistent conclusions.

In the revision, we have explained the rationale for using the nearest-neighbor hot-deck imputation by matching cells with similar core module (Lines 143-150).

"*Previous work determined parameter distributions for only the cognate receptor module (and the core module) that provided the best fit for the single ligand experimental data*

(Figure 1A, Step 1), and other receptor modules parameter information is missing. To simulate stimulus responses to more than two ligands, we imputed the other ligand–receptor module parameters using shared core-module parameters as common variables and employing nearest-neighbor hot-deck imputation (35). In this setup, the core module functions as an “anchor” to harmonize two or more receptor-specific parameter distributions. This was achieved by by minimizing Euclidean distance between the core module parameters associated with the independently parameterized single-ligand models (Figure 1A, Step 2). ”

In Guo et al. (2025) (see Supplementary Figure S11), the nearest-neighbor hot-deck imputation approach (core module similarity matching method) was compared with other approaches, including random matching and rescaled-similarity matching. The results show that, after matching, the core module method best preserves the single-ligand stimulus signaling codon distributions. For the reviewer’s convenience, we have also appended the figure in the response to Reviewer 1, Comment 11.

The advantage of our workflow is that it does not need to be fit to new experimental data and still gives reliable predictions on signaling dynamics. For the reviewer’s interest, we have tried to fit core-matched single cell models with two receptor modules. As fitting parameters require sufficiently large and high-quality datasets, single-ligand stimulation data with more than 1,000 cells can be adequate to estimate 6~7 parameters (Guo et al., 2025) (approx. 1400 cells to 2000 cells per ligand). However, our current experimental dataset for combinatorial-ligand conditions contains only 500~1,000 cells, and we have tested these datasets but results show a poor fit of heterogeneous signaling dynamics. This is due to an insufficient number of cells for estimating 8~10 parameters. We estimate that at least ~1,500 cells would be needed for reliable parameter estimation under dual-ligand stimulation (and more cells may be needed for combinatorial ligand stimuli involving more ligands). This is currently not feasible to obtain for mixed ligands given the large number of combinatorial conditions.

Overall, in this paper, the nearest-neighbor hot-deck imputation approach is presented as a feasible and acceptable approach that best reflects our current understanding of the signaling network. Importantly, it helps identify potential gaps by highlighting discrepancies between model predictions and experimental observations.

2. (a) The refined model posits competitive, saturable endosomal transport for CpG and pIC, but no direct measurements of endosomal uptake rates or compartmental saturation thresholds are provided, leaving the Hill parameters under-constrained. The authors could produce dose-response curves for CpG and pIC individually and in combination across a range of concentrations to fit the Hill parameters for competitive uptake. (b) If this is out of scope for this paper, the authors should at least comment on

why the endosome hypothesis is better than others e.g. crosstalks and other parallel pathway activations. Especially given that even the refined model simulations with Hill equations for CpG and pIC do not quite match with the experimental data (Fig 2 B,E).

Response: (a) The reviewer's comments helped us to improve our work by employing the Michaelis-Menten Kinetics for substrate competition reactions, which increases the mathematic rigor of the CpG-pIC competition model. In this updated model, there is no free parameters to tune, as all the Vmax, Kd, should be consistent with the single-ligand scenario. And the Hill is same as single-ligand case, equal to 1.

The comments on examining dose-response curves for CpG and pIC inspired us to extend the dose-response curves for all ligand pair combination, allowing us to identify the synergy in low-dose ligand pairs and antagonism for high-dose LPS-Pam, besides CpG-pIC (new Figure 4 & 5).

(b) Regarding alternative hypotheses for antagonism—such as crosstalk or parallel-pathway activation: any antagonistic effect would have to arise from negative regulation acting within the first 30 min. However, IκBα-mediated feedback only becomes appreciable after ~30 min (Hoffmann et al., 2002), and A20-dependent attenuation requires ≥2 h (Werner et al., 2005). Beyond these delayed feedback, NFκB activation depends primarily on phosphorylation and K63-linked ubiquitination, for which no mechanism produces true antagonism; at most, combinatorial inputs saturate the response to the level of the strongest single ligand. We have added this rationale to the Discussion to explain why we favor the endosome saturation hypothesis over other mechanisms (Lines 459-465). While this may not capture every nuance, it represents the simplest model extension capable of reproducing the observed antagonism.

3. Authors asses the distinguishability of single-ligand stimuli and combinatorial ligands stimuli using the simulations from the refined model. While this is informative, the simulated data could propagate deviations from the experimental data to the classifiers. How would the classifiers fare when the experimental data is used to assess the single-stimulus distinguishability? The authors could use the experimental data they already have and confirm their main claim of the paper, that cells retain stimulus-response specificity even with multiple ligand exposure. In short, how would Fig 3E look when trained/validated on available experimental data?

Response: We thank the reviewer's valuable comments, and they helped us strengthen the rigor of our analysis by incorporating cross-model testing. Specifically, we refined our analysis of ligand presence/absence classification by including ROC AUC and balanced accuracy metrics. This adjustment accounts for the fact that the experimental data did not cover all combinatorial conditions, thereby mitigating potential biases from data imbalance and threshold choice. The experimental results are qualitatively

consistent with the simulations, though—as expected—they show somewhat lower ligand distinguishability compared to the noise-free simulated dataset. We have updated Figures 3E–F (previously Figure 3E), added Figure S8, and revised the manuscript accordingly (Lines 292–301). For the reviewer's convenience, we have also pasted in the revised manuscript text below.

"Classifiers trained to distinguish TNF-present from TNF-absent conditions achieved a Receiver Operating Characteristic-Area Under the Curve (ROC AUC) of 0.96, significantly above the 0.5 baseline (Figure 3D, Figure S8A). Extending this analysis to other ligands, cells detected LPS (0.85), Pam (0.84), pIC (0.73), and CpG (0.63) in mixtures (Figure 3D, S8A). Using experimental data from double- and triple-ligand stimuli (Figure 1D), ROC AUC values were TNF 0.74, LPS 0.74, Pam 0.66, pIC 0.75, and CpG 0.66 (Figure 3E, S8B). Classifier accuracies yielded consistent results (Figure S8C-D). These results indicated a remarkable capability of preserving ligand-specific dynamic features within complex NFκB signal trajectories that enable nuclear detection of extracellular ligands even in complex stimulus mixtures."

4. While the approach of presented here with multiple simultaneous ligand exposures is a major step towards the in vivo-like conditions, the temporal aspect is still missing. That is, temporal phasing i.e. sequential exposure to multiple ligands as one would expect in vivo rather than all at once. This is probably out of scope for this paper but the authors could comment how how their work could be taken forward in such direction and would the SRS be better or worse in such conditions.

Response: We thank the reviewer for this insightful comment. We have added "the temporal aspect of multiple ligand exposures" to the discussion (Lines 503-510), and we pasted the corresponding paragraph here for reviewer's references (black fonts are previous version, and blue fonts is the revised new texts):

"*Cells may be expected to interpret not only the combination of signals but also their timing and duration to mount appropriate transcriptional responses (58, 59). For example, acute inflammation integrates pathogen-derived cues with pro- and anti-inflammatory signals over a timeframe of hours to days (58), to coordinate the pathogen removal and tissue repairing process. Investigating sequential stimulus combinations in our model is therefore crucial for understanding how cells process complex physiological inputs. Simulations that account for longer timescales may require additional feedback mechanisms, as described in some of our previous studies for NFκB (15, 60).* "

5. There is no caption for Figure 3F in the figure legend nor a reference in the main text.

Response: In the revised manuscript we actually removed Figure 3F.

Significance

General assessment: This is a good manuscript in it's present form which could get better with revision. There needs more supporting data and validation to back the main claim presented in the manuscript.

Significance/impact/readership: When revised this manuscript could be of interest to a broad community involving single cells biology, cell and immune signaling, and mathematical modeling. Especially the models presented here could be used a starting point to more complex and detailed modeling approaches.

Response: We thank the reviewer for this endorsement. The reviewer's constructive suggestion helped us significantly improve the clarity and rigor of our main conclusion.

In summary, we have strengthened the computational framework in several ways. We improved the model's fit to experimental single-ligand training data and reformulated the antagonistic CpG-pIC model using Michaelis–Menten kinetics, thereby reducing parameter arbitrariness and increasing mechanistic interpretability. These changes led to better agreement between model predictions and experimental observations for combinatorial ligand responses (Updated Figure 2 and Figure S2), which we hope will further increase experimentalists' confidence in the modeling results. We have also validated one key conclusion ("cells retain stimulus-response specificity even with multiple ligand exposure") using the experimental dataset, and it aligns with the model predictions.

In addition, we have further extended our analysis and the scope. Inspired by the reviewer's advice (and Reviewer 3's comment 1b) on dose-combination study for CpG-pIC pair, we expanded our research to dose-response relationships for all dual-ligand combinations (Lines 302-406, Figure 4-5). This additional comprehensive analysis allowed us to identify the mechanism of synergistic and antagonistic effects in single-cell responses and to pinpoint the corresponding dose ranges among different ligand pairs.

Interestingly, we found that IKK ultrasensitive activation may lead to low-dose ligand combinations synergistic response for single cells. We also found that CD14 uptake competition between LPS and Pam may lead to antagonistic/non-integrative combination. Our simulation-based finding of non-integrative combination of LPS-Pam stimuli aligns with previous independent experimental finding of non-integrative response for LPS and Pam combination (Kellogg et al., 2017), and this independent experimental study validated our model prediction.

We further analyzed stimulus-response specificity under conditions predicted to exhibit synergy or antagonism. Our results indicate that antagonistic combinations of ligands can increase stimulus-response specificity in the context of ligand mixtures.

Reviewer #3

Evidence, reproducibility and clarity

The authors investigate experimentally single macrophages' NF-kB responses to five ligands, separately and to 3 pairs of ligands. Using the single ligand stimulations, they train an existing mathematical model to replicate single-cell NF-kB nuclear trajectories. From what I understand, for each single cell trajectory in response to a given ligand, the best fit parameters of the core module and the receptor module (specific for the given ligand) are found.

Then (again, from what I understand), single ligand models are used to generate responses to combinations of ligands. The parametrizations of single ligand models (to be combined) are chosen to have the most similar core modules. It is not described how the responses to more than one ligand are calculated - I expect that respective receptor modules work in parallel, providing signals to the core module. After observing that the response to CpG+pIC is lower (in terms of duration and total) than for CpG alone, the model is modified to account for competition for endosomal transport required by both ligands.

Having the trained model, simulations of responses to all 31 combinations of ligands are performed, and each NF-κB trajectory is described by six signaling codons-Speed, Peak, Duration, Total, Early vs. Late, and Oscillations. Next, these codons are used to reconstruct (using a random forest model) the stimuli (which may be the combination of ligands). The single and even the two ligand stimuli are relatively well recognized, which is interpreted as the ability of macrophages to distinguish ligands even if present in combination.

We thank the reviewer for careful reading of the manuscript.

Major comments

1) The demonstrated ability to recognize stimuli is based on several key assumptions that can hardly be met in reality.

Response: We thank the reviewer for this comment, which prompted us to carefully reflect on the rigor of our work, inspired us to extend our analysis to a broad range of ligand-dose combinations, and helped us improve clarifying the limitations of our approach. Please see our detailed responses below.

a) The cell knows the stimulation time, and then it can use speed as a codon. Look on fig. S4A: The trajectories in response to pIC are similar to those in response to TNF, but just delayed.

Response: We thank the reviewer for this comment. We updated the model parameterization to better fit to the single-ligand pIC condition (Lines 557-559). In the updated model, the simulated responses to TNF and pIC are quite different (Fig. S2A-B, Fig. S5A-B). Specifically, the Peak, Duration, EarlyVsLate, and Total signaling codons have different values. In addition, the literature suggests that timing difference of NFκB activation are sufficient to elicit differences in downstream gene expression responses, especially for the early response genes (ERG) and intermediate response genes (ING) (Figure 1 in Ando, et al, 2021). For reviewer's convenience, we have also appended the figures. Specifically, within the first 60 minutes, ctrl exhibit higher Speed of NFκB activation, and the NFκB regulated ERG and ING show differences in the first 60 minutes (Below Fig 1a,b). Ando et al then identified the gene regulatory mechanism that is able to distinguish between differences in the Speed codon. Importantly, this mechanism does not require knowledge of t=0, i.e. when the timer was started.

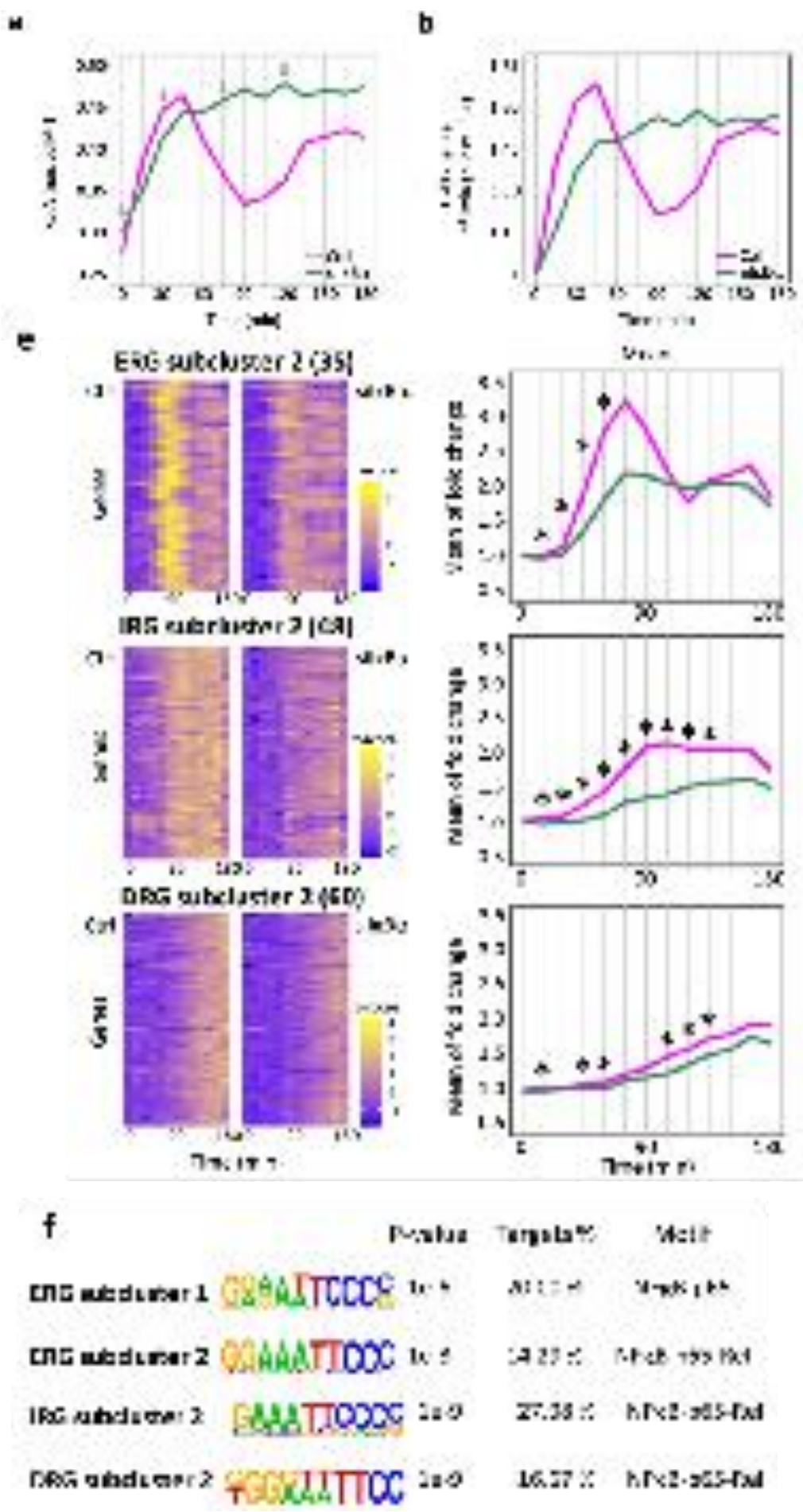

Figure 1 From Ando et al., 2021. Time-course nuclear NFκB abundance. (**a**) and fold change (**b**) from fixed-cell immunofluorescence of MCF-7 cells treated with 1 ng mL^{-1} TNF in Ctrl (Control) and siIκBα (IκBα knockdown). Line graphs represent the means of two biological replicates. Number of cells used to calculate the mean for each time point ranged from 1336 to 2374 cells. Gray arrows indicate the time points where RNA-seq data was measured. Statistical significance was observed for interpolated (same method used in mathematical modeling) time-course nuclear NFκB abundance (p value: 1.039e−4) and fold change (p value < 2.2e−16) between Ctrl and siIκBα from before stimulation and 75 min (the first peak time point of early response genes) after stimulation by one-tailed Wilcoxon rank-sum test. **e** Two subclusters for each TNF-induced cluster (ERG, IRG, and DRG) in Ctrl (outlier PCSK5 excluded in line graph). For these 6 subclusters, statistical tests were performed for fold change in expression between Ctrl and siIκBα (*p value < 0.01 by one-tailed Wilcoxon rank-sum test). **f** κB motifs that were enriched at promoter regions (±50 bps TSS) of ERG subclusters 1 and 2, IRG subcluster 2, and DRG subcluster 2.

The signaling codon Speed, which is based on derivatives, is one way to quantify such timing differences in activation. It was selected from a library of more than 900 different dynamic features using an information maximizing algorithm (Adelaja et al., 2021). It is possible that other ways of measuring time, e.g. time to half-max, might not be distinguished that well by these regulatory mechanisms.

b) The increase of stimulus concentration typically increases Peak, Duration, and Total, so a similar effect can be achieved by changing the ligand or concentration.

Response: This ("the increase of stimulus concentration typically increases Peak, Duration, and Total") is not an assumption. What the reviewer described ("a similar effect can be achieved by changing the ligand or concentration") may occur or may not. The six informative signaling codons can vary under different ligands or doses. For example, with increasing doses of Pam, the NFκB response shows a higher peak, potentially making it appear more like LPS stimulation. However, as the Pam dose increases, the response duration decreases, which distinguishes it from LPS stimulation (See experimental data shown in Figure 4A, second row, and Figure 3A, second row in Luecke et al., (2024), we also pasted the corresponding figure below for reviewer's convenience).

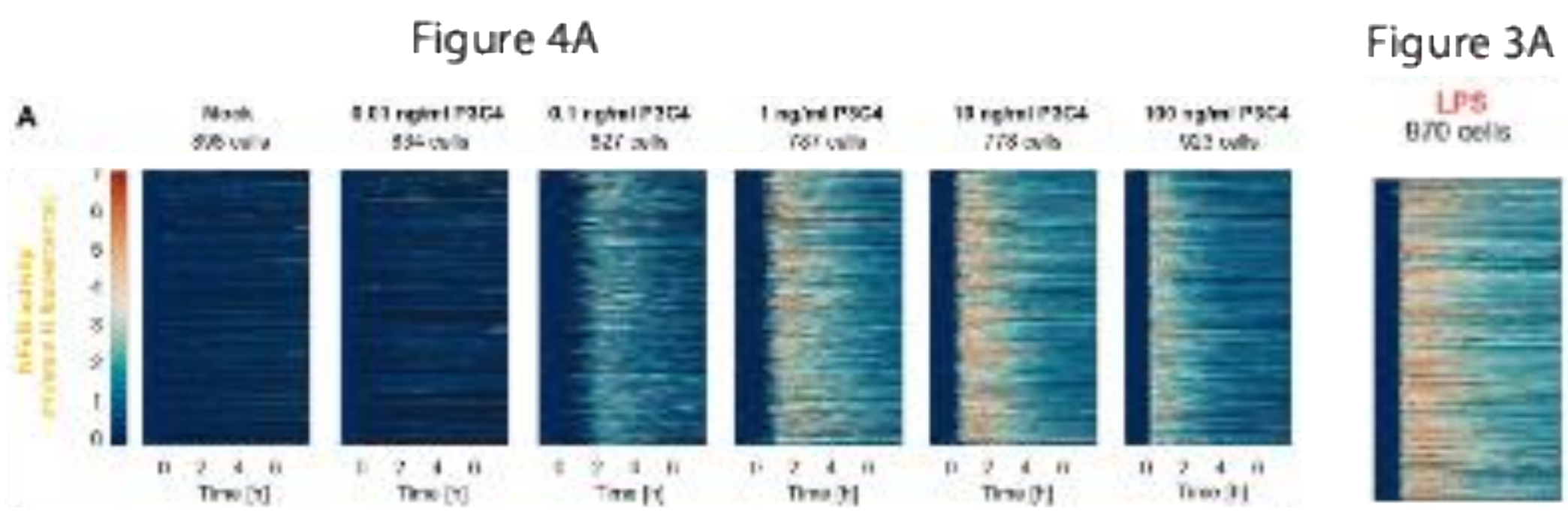

Figure 4A and Figure 3A from Luecke et al., (2024). Figure 4A: NFκB activity dynamics in the single cells in response to 0, 0.01, 0.1, 1, 10, and 100 ng/ml P3C4 stimulation. Eight hours were measured by fluorescence microscopy of reporter hMPDMs. Each row of the heatmap represents the p38 or NFκB signaling trajectory of one cell. Trajectories are sorted by the maximum amplitude of p38 activity. Data from two pooled biological replicates are depicted. Total # of cells: 898, 834, 827, 787, 778, and 923. Figure 3A: NFκB activity dynamics in the single cells in response to 100 ng/ml LPS stimulation. Eight hours were measured by fluorescence microscopy of reporter hMPDMs. Each row of the heatmap represents the NFκB signaling trajectory of one cell (with p38 measured shown in the original paper). Trajectories are sorted by the maximum amplitude of p38 activity. Data from two pooled biological replicates are depicted.

Inspired by the reviewer's comment (and also Reviewer 2's comments), in the revision, we expanded our research to dose-response relationships for all dual-ligand combinations (Lines 302-406, Figure 4-5). This additional comprehensive analysis allowed us to identify the mechanism of synergistic and antagonistic effects in single-

cell responses and to pinpoint the corresponding dose ranges among different ligand pairs.

Interestingly, we found that IKK ultrasensitive activation may lead to synergistic responses to low-dose ligand combinations but only in a subset of single cells. We also found that CD14 uptake competition between LPS and Pam may lead to antagonistic/non-integrative combination. Our simulation-based finding of non-integrative combination of LPS-Pam stimuli aligns with previous independent experimental findings of non-integrative response for LPS and Pam combination (Kellogg et al., 2017).

c) Distinguishing a given ligand in the presence of some others, even stronger bases, on the assumption that these ligands were given at the same time, which is hardly justified.

Response: We agree with the reviewer that ligands could be given at different times. Considering time delays between ligands (the inset and also removal) dramatically adds to the combinatorial complexity. Some initial studies by the Tay lab are beginning to explore some scenarios of time-shifted ligand pairs (Wang et al 2025). Here we focus on a systematic exploration of all ligand combinations at 6 different doses. The fact that we do not consider time delays is not an assumption but admittedly a limitation that may well be addressed in future studies. We have included a brief discussion of this issue in the discussion (Lines 503-514). We've appended here for reviewer's convenience.

"*Cells may be expected to interpret not only the combination of signals but also their timing and duration to mount appropriate transcriptional responses (Kumar et al., 2004; Son et al., 2023). For example, acute inflammation integrates pathogen-derived cues with pro- and anti-inflammatory signals over a timeframe of hours to days (Kumar et al., 2004), to coordinate the pathogen removal and tissue repairing process. Investigating sequential stimulus combinations in our model is therefore crucial for understanding how cells process complex physiological inputs. Simulations that account for longer timescales may require additional feedback mechanisms, as described in some of our previous studies for NFκB (Werner et al., 2008, 2005).* "

We would like to suggest that despite (or maybe because) limiting our study to coincident stimuli, we made some noteworthy discoveries.

2) For single ligands, it would be nice to see how the random forest classifier works on experimental data, not only on in silico data (even if generated by a fitted model).

Response: This comment and Reviewer 2 comment 3 have helped us strengthen the rigor of our analysis by incorporating cross-model testing. We pasted the response below.

Specifically, we refined our analysis of ligand presence/absence classification by including ROC AUC and balanced accuracy metrics. This adjustment accounts for the fact that the experimental data did not cover all combinatorial conditions, thereby mitigating potential biases from data imbalance and threshold choice. The experimental results are qualitatively consistent with the simulations, though—as expected—they show somewhat lower ligand distinguishability compared to the noise-free simulated dataset. We have updated Figures 3E–F (previously Figure 3E), added Figure S8, and revised the manuscript accordingly (Lines 292–301). For the reviewer's convenience, we have also included the revised manuscript text below.

"Classifiers trained to distinguish TNF-present from TNF-absent conditions achieved a Receiver Operating Characteristic-Area Under the Curve (ROC AUC) of 0.96, significantly above the 0.5 baseline (Figure 3D, Figure S8A). Extending this analysis to other ligands, cells detected LPS (0.85), Pam (0.84), pIC (0.73), and CpG (0.63) in mixtures (Figure 3D, S8A). Using experimental data from double- and triple-ligand stimuli (Figure 1D), ROC AUC values were TNF 0.74, LPS 0.74, Pam 0.66, pIC 0.75, and CpG 0.66 (Figure 3E, S8B). Classifier accuracies yielded consistent results (Figure S8C-D). These results indicated a remarkable capability of preserving ligand-specific dynamic features within complex NFκB signal trajectories that enable nuclear detection of extracelular ligands even in complex stimulus mixtures."

3) My understanding of ligand discrimination is such that it is rather based on a combination of pathways triggered than solely on a single transcription factor response trajectory, which varies with ligand concentration and ligand concentration time profile (no reason to assume it is OFF-ON-OFF). For example, some of the considered ligands (plC and CpG) activate IRF3/IRF7 in addition to NF-kB, which leads to IFN production and activation of STATs. This should at least be discussed.

Response: We thank the reviewer for this comment and fully agree. In the previous version, we discussed different signaling pathways combinatorically distinguishing stimulus. In the revision, we have extended this discussion to include the example of pIC and CpG activation, as suggested (Lines 515-522). We pasted the corresponding text below.

"Furthermore, innate immune responses do not solely rely on NFκB but also involve the critical functions of AP1, p38, and the IRF3-ISGF3 axis. The additional pathways are likely activated in a coordinated manner and provide additional information (Luecke et al., 2021). This is exemplified by the studies demonstrating synergistic effects between CpG and pIC in inhibiting tumor growth and promoting cytokine production (Huang et al., 2020), such as IFNβ and TNFα, whose expression is also regulated by the IRF and MAPK signaling pathways (Luecke et al., 2021; Sheu et al., 2023)*. Therefore the inclusion of parallel pathways of AP1 and MAPK, as well as the type I interferon network*

(Cheng et al., 2015; Davies et al., 2020; Hanson and Batchelor, 2022; Luecke et al., 2024; Paek et al., 2016; Peterson et al., 2022) *are next steps for expanding the mathematical models presented here."*

Technical comments

1) Reference 25: X. Guo, A. Adelaja, A. Singh, W. Roy, A. Hoffmann, Modeling single-cell heterogeneity in signaling dynamics of macrophages reveals principles of information transmission. Nature Communications (2025)
does not lead to any paper with the same or a similar title and author list. This Ref is given as a reference to the model. Fortunately, Ref 8 is helpful. Nevertheless, authors should include a schematic of the model.

Response: We apologize for the paper not being accessible on time. It is now. We have also added a schematic of the model as suggested (Figure S1) and have added detailed description of the model and simulations in introduction (Lines 95-106), results (Lines 129-141), and methods (***Simulation of heterogenous NFκB dynamical responses***).

2) Also Mendeley Data DOI:10.17632/bv957x6frk.1 and GitHub https://github.com/Xiaolu-Guo/Combinatorial_ligand_NFkB lead to nowhere.

Response: We thank the reviewer for this comment, and we have made the GitHub codes public. Mendeley Data DOI:10.17632/bv957x6frk.1 can be accessed via the shared link: https://data.mendeley.com/preview/bv957x6frk?a=6d56e079-d7b0-482e-951f-8a8e06ee8797

and will be public once the paper accepted.

3) Dataset 1 is not described. Possibly it contains sets of parameters of receptor modules (different numbers of sets for each module, why?), but the names of parameters never appear in the text, which makes it impossible to reproduce the data.

Response: We thank the reviewer for this comment, and we have added the description of the dataset (**S3 SupplementaryDataset2_NFkB_network_single_cell_parameter_distribution.xlsx**) and added the parameter names in the methods (**Simulation of heterogenous NFκB dynamical responses**).

4) It is difficult to understand how the simulations in response to more than one ligand are performed.

Response: We thank the reviewer for this comment, and we have improved the explanation of the methods (Results, Lines 145-152) and included a detailed description

of the model and simulations for combinatorial ligands (Methods, **Predicting heterogeneous single-cell responses to combinatorial-ligand stimulation**).

Significance

A lot of work has been done, the methodology is interesting, but the biological conclusions are overstated.

Response: We thank the reviewer for their interest in the methodology. We have revised the title, the abstract, and added the discussion about our finding to more accurately document what we have found. In the revision, we have increased the clarity and rigor of the work. For the key conclusion that macrophages maintain some level of NFκB signaling fidelity in response to ligand mixtures, we have validated the binary classifier results on experimental data as reviewer suggested.

In the revision, we have also extended our methodology to explore further, the dose-response curves for different dosage combination for ligand pairs. This further work allowing us identified the synergistic and antagonistic regimes. By comparing the stimulus response specificity for antagonistic model vs the non-antagonistic model, we demonstrated that signaling antagonism may increase the distinguishability of presence or absence of specific ligands within complex ligand mixtures. This provides a mechanism of how signaling fidelity is maintained to the surprising degree we reported.

REFERENCES

Adelaja, A., Taylor, B., Sheu, K.M., Liu, Y., Luecke, S., Hoffmann, A., 2021. Six distinct NFκB signaling codons convey discrete information to distinguish stimuli and enable appropriate macrophage responses. Immunity 54, 916-930.e7. https://doi.org/10.1016/j.immuni.2021.04.011

Akira, S., Takeda, K., 2004. Toll-like receptor signalling. Nat Rev Immunol 4, 499–511. https://doi.org/10.1038/nri1391

Andridge, R.R., Little, R.J.A., 2010. A Review of Hot Deck Imputation for Survey Non-response. Int Stat Rev 78, 40–64. https://doi.org/10.1111/j.1751-5823.2010.00103.x

Cheng, Z., Taylor, B., Ourthiague, D.R., Hoffmann, A., 2015. Distinct single-cell signaling characteristics are conferred by the MyD88 and TRIF pathways during TLR4 activation. Sci Signal 8, ra69. https://doi.org/10.1126/scisignal.aaa5208

Davies, A.E., Pargett, M., Siebert, S., Gillies, T.E., Choi, Y., Tobin, S.J., Ram, A.R., Murthy, V., Juliano, C., Quon, G., Bissell, M.J., Albeck, J.G., 2020. Systems-Level Properties of EGFR-RAS-ERK Signaling Amplify Local Signals to Generate Dynamic Gene Expression Heterogeneity. Cell Systems 11, 161-175.e5. https://doi.org/10.1016/j.cels.2020.07.004

Guo, X., Adelaja, A., Singh, A., Roy, W., Hoffmann, A., 2025a. Modeling single-cell heterogeneity in signaling dynamics of macrophages reveals principles of information transmission. Nature Communications.

Guo, X., Adelaja, A., Singh, A., Wollman, R., Hoffmann, A., 2025b. Modeling heterogeneous signaling dynamics of macrophages reveals principles of information transmission in stimulus responses. Nat Commun 16, 5986. https://doi.org/10.1038/s41467-025-60901-3

Hanson, R.L., Batchelor, E., 2022. Coordination of MAPK and p53 dynamics in the cellular responses to DNA damage and oxidative stress. Molecular Systems Biology 18, e11401. https://doi.org/10.15252/msb.202211401

Huang, Y., Zhang, Q., Lubas, M., Yuan, Y., Yalcin, F., Efe, I.E., Xia, P., Motta, E., Buonfiglioli, A., Lehnardt, S., Dzaye, O., Flueh, C., Synowitz, M., Hu, F., Kettenmann, H., 2020. Synergistic Toll-like Receptor 3/9 Signaling Affects Properties and Impairs Glioma-Promoting Activity of Microglia. J. Neurosci. 40, 6428–6443. https://doi.org/10.1523/JNEUROSCI.0666-20.2020

Kellogg, R.A., Tian, C., Etzrodt, M., Tay, S., 2017. Cellular Decision Making by Non-Integrative Processing of TLR Inputs. Cell Rep 19, 125–135. https://doi.org/10.1016/j.celrep.2017.03.027

Kumar, R., Clermont, G., Vodovotz, Y., Chow, C.C., 2004. The dynamics of acute inflammation. Journal of Theoretical Biology 230, 145–155. https://doi.org/10.1016/j.jtbi.2004.04.044

Luecke, S., Guo, X., Sheu, K.M., Singh, A., Lowe, S.C., Han, M., Diaz, J., Lopes, F., Wollman, R., Hoffmann, A., 2024. Dynamical and combinatorial coding by MAPK p38 and NFκB in the inflammatory response of macrophages. Molecular Systems Biology 20, 898–932. https://doi.org/10.1038/s44320-024-00047-4

Luecke, S., Sheu, K.M., Hoffmann, A., 2021. Stimulus-specific responses in innate immunity: Multilayered regulatory circuits. Immunity 54, 1915–1932. https://doi.org/10.1016/j.immuni.2021.08.018

Paek, A.L., Liu, J.C., Loewer, A., Forrester, W.C., Lahav, G., 2016. Cell-to-Cell Variation in p53 Dynamics Leads to Fractional Killing. Cell 165, 631–642. https://doi.org/10.1016/j.cell.2016.03.025
Peterson, A.F., Ingram, K., Huang, E.J., Parksong, J., McKenney, C., Bever, G.S., Regot, S., 2022. Systematic analysis of the MAPK signaling network reveals MAP3K-driven control of cell fate. Cell Systems 13, 885-894.e4. https://doi.org/10.1016/j.cels.2022.10.003
Sheu, K.M., Guru, A.A., Hoffmann, A., 2023. Quantifying stimulus-response specificity to probe the functional state of macrophages. Cell Systems 14, 180-195.e5. https://doi.org/10.1016/j.cels.2022.12.012
Son, M., Wang, A.G., Keisham, B., Tay, S., 2023. Processing stimulus dynamics by the NF-κB network in single cells. Exp Mol Med 55, 2531–2540. https://doi.org/10.1038/s12276-023-01133-7
Stuart, T., Butler, A., Hoffman, P., Hafemeister, C., Papalexi, E., Mauck, W.M., Hao, Y., Stoeckius, M., Smibert, P., Satija, R., 2019. Comprehensive Integration of Single-Cell Data. Cell 177, 1888-1902.e21. https://doi.org/10.1016/j.cell.2019.05.031
Werner, S.L., Barken, D., Hoffmann, A., 2005. Stimulus Specificity of Gene Expression Programs Determined by Temporal Control of IKK Activity. Science 309, 1857–1861. https://doi.org/10.1126/science.1113319
Werner, S.L., Kearns, J.D., Zadorozhnaya, V., Lynch, C., O'Dea, E., Boldin, M.P., Ma, A., Baltimore, D., Hoffmann, A., 2008. Encoding NF-kappaB temporal control in response to TNF: distinct roles for the negative regulators IkappaBalpha and A20. Genes Dev 22, 2093–2101. https://doi.org/10.1101/gad.1680708

24th Mar 2026

Manuscript Number: MSB-2026-13639-T
Title: Macrophage response specificity to ligand mixtures is improved by signaling pathway antagonism
Author: Xiaolu Guo
Supriya Sen
Julian Gonzalez
Alexander Hoffmann

Dear Alex,

Thank you for submitting your manuscript to Molecular Systems Biology. We have now received the enclosed reports from the three reviewers who evaluated the previous version of the manuscript at Review Commons. As you will see from the comments below, Reviewers #1 and #2 support publication of the manuscript after addressing a number of relatively minor issues. Reviewer #3, however, continues to raise concerns regarding two central claims: (1) that stimulus-response specificity is partly encoded in NF-κB dynamics, and (2) that temporal coding conveys ligand identity.

During our pre-decision cross-commenting process, both Reviewers #1 and #2 acknowledged Reviewer #3's concerns. The reviewers' additional comments are included following their reports.

In light of these comments, our recommendations are as follows:

- You may consider performing mutual information analyses to quantify how much stimulus information is contained in NF-κB dynamics. This analysis could provide additional support for your conclusions but is optional.

- Independently, please carefully review the wording of the abstract, conclusions, and other relevant sections to ensure that the claims accurately reflect what the data demonstrate. Particular attention should be paid to terms such as "encode," "recognize," "temporal coding," and "identity," as well as related wording, ensuring that the language aligns precisely with the evidence and does not imply stronger conclusions than are supported by the data.

Please also address all other remaining issues raised by the reviewers. As our editorial policy allows a single round of major revision, it is essential that your responses are as complete as possible. We encourage you to take this opportunity to clarify the key claims and ensure that the manuscript's conclusions are fully supported. Please feel free to contact me if you wish to discuss any of the points in further detail.

On a more editorial level, we would ask you to address the following issues:

- Please provide a .docx formatted version of the manuscript text (including legends for main figures, EV figures and tables). Please make sure that the changes are highlighted to be clearly visible.

- Please provide individual production quality figure files as .eps, .tif, .jpg (one file per figure). When assembling figures, please refer to our figure preparation guideline in order to ensure proper formatting and readability in print as well as on screen: https://media.springernature.com/original/springer-cms/rest/v1/content/27825798/data/v1

-Please provide a .docx formatted letter INCLUDING the reviewers' reports and your detailed point-by-point responses to their comments. As part of the EMBO Press transparent editorial process, the point-by-point response is part of the Review Process File (RPF), which will be published alongside your paper.

-Please note that all corresponding authors are required to supply an ORCID ID for their name upon submission of a revised manuscript.

-We replaced Supplementary Information with Expanded View (EV) Figures and Tables that are collapsible/expandable online. A maximum of 5 EV Figures can be typeset. EV Figures should be cited as 'Figure EV1, Figure EV2" etc... in the text and their respective legends should be included in the main text after the legends of regular figures.

Additional Tables/Datasets should be labeled and referred to as Table EV1, Dataset EV1, etc. Legends have to be provided in a separate tab in case of .xls files. Alternatively, the legend can be supplied as a separate text file (README) and zipped together with the Table/Dataset file.

For the figures and tables that you do NOT wish to display as Expanded View figures, they should be bundled together with their

legends in a single PDF file called *Appendix*, which should start with a short Table of Content. Each legend should be below the corresponding Figure/Table in the Appendix. Appendix figures and tables should be referred to in the main text as: "Appendix Figure S1, Appendix Figure S2, Appendix Table S1" etc. See detailed instructions regarding expanded view here: https://link.springer.com/journal/44320/submission-guidelines#cms-Expanded-View-data.

-Before submitting your revision, primary datasets (and computer code, where appropriate) produced in this study need to be deposited in an appropriate public database.
Please remember to provide a reviewer password if the datasets are not yet public.
The accession numbers and database should be listed in a formal "Data Availability" section (placed after Materials & Method) that follows the model below (see also https://link.springer.com/partners/embo-press/editorial-policies#Data%20availability%20statement). Please note that the Data Availability Section is restricted to new primary data that are part of this study.
Data availability
The datasets (and computer code) produced in this study are available in the following databases:
- RNA-Seq data: Gene Expression Omnibus GSE46843 (https://www.ncbi.nlm.nih.gov/geo/query/acc.cgi?acc=GSE46843)
- [data type]: [name of the resource] [accession number/identifier/doi] ([URL or identifiers.org/DATABASE:ACCESSION])
*** Note - All links should resolve to a page where the data can be accessed. ***

- At EMBO Press we ask authors to provide source data for the main manuscript figures. You will receive a separate email with instructions for providing source data with your revised manuscript, including how to upload and organize the files.
Additional information on source data and instruction on how to label the files are available < https://link.springer.com/journal/44320/submission-guidelines#cms-Source-data >

- Our journal encourages inclusion of *data citations in the reference list* to directly cite datasets that were re-used and obtained from public databases. Data citations in the article text are distinct from normal bibliographical citations and should directly link to the database records from which the data can be accessed. In the main text, data citations are formatted as follows: "Data ref: Smith et al, 2001". In the Reference list, data citations must be labeled with "[DATASET]". A data reference must provide the database name, accession number/identifiers and a resolvable link to the landing page from which the data can be accessed at the end of the reference. Further instructions are available at < https://link.springer.com/journal/44320/submission-guidelines#cms-Reference-guidelines >.

- We updated our journal's competing interests policy in January 2022 and request authors to consider both actual and perceived competing interests. Please review the policy (https://link.springer.com/partners/embo-press/editorial-policies#Competing%20interest%20disclosures) and update your competing interests if necessary.
Please use the heading "Disclosure statement and competing interests".

- All Materials and Methods need to be described in the main text using our 'Structured Methods' format. According to this format, the Methods section includes a Reagents and Tools Table (listing key reagents, experimental models, software and relevant equipment and including their sources and relevant identifiers) followed by a Methods and Protocols section describing the methods, ideally using a step-by-step protocol format. The aim is to facilitate adoption of the methodologies across labs.

Please download and fill our Reagents and Tools Table template (.docx), which you can find in our author guidelines: https://link.springer.com/journal/44320/submission-guidelines#structuredmethods.

When submitting your revised manuscript, please do not include the Reagents and Tools Table in the Methods section of the manuscript but upload it as a separate file choosing the file type "Reagent Table".

-Regarding data quantification:
Please ensure to specify the name of the statistical test used to generate error bars and P values, the number (n) of independent experiments (please specify technical or biological replicates) underlying each data point and the test used to calculate p-values in each figure legend. Discussion of statistical methodology can be reported in the materials and methods section, but figure legends should contain a basic description of n, P and the test applied.
Graphs must include a description of the bars and the error bars (s.d., s.e.m.).
Please also include scale bars in all microscopy images.

- Please provide a "standfirst text" summarizing the study in one or two sentences (approximately 250 characters, including space), three to four "bullet points" highlighting the main findings and a " visual abstract" (550px width and 400-600 px height, PNG format) to highlight the paper on our homepage.
Please refer to published papers for examples.

When you resubmit your manuscript, please download our CHECKLIST (https://media.springernature.com/original/springer-cms/rest/v1/content/27825796/data/v1) and include the completed form in your submission.
Please note that the Author Checklist will be published alongside the paper as part of the transparent process .

If you feel you can satisfactorily deal with these points and those listed by the referees, you may wish to submit a revised version of your manuscript. Please attach a covering letter giving details of the way in which you have handled each of the points raised by the referees. A revised manuscript may be once again subject to review and you probably understand that we can give you no guarantee at this stage that the eventual outcome will be favorable.

I look forward to receiving the revised manuscript soon.

Kind regards,
Jingyi

Jingyi Hou, PhD
Senior Editor
Molecular Systems Biology

We realize that it is difficult to revise to a specific deadline. In the interest of protecting the conceptual advance provided by the work, we recommend a revision within 3 months (22nd Jun 2026). Please discuss the revision progress ahead of this time with the editor if you require more time to complete the revisions. Use the link below to submit your revision:

https://msb.msubmit.net/cgi-bin/main.plex

IMPORTANT: Please read our guidelines for revised submissions: https://link.springer.com/journal/44320/submission-guidelines#cms-Revised-submissions

*** PLEASE NOTE *** As part of the EMBO Press transparent editorial process initiative (see our Editorial at https://dx.doi.org/10.1038/msb.2010.72), Molecular Systems Biology publishes online a Review Process File with each accepted manuscripts. This file will be published in conjunction with your paper and will include the anonymous referee reports, your point-by-point response and all pertinent correspondence relating to the manuscript. If you do NOT want this File to be published, please inform the editorial office at contact@molsystbiol.org within 14 days upon receipt of the present letter.

Reviewer #1:

Many thanks for the updated manuscript and your work in responding to the reviewer comments. I have no further questions related to the previous comments, and I am satisfied that all issues are addressed.

I have several comments on the new material introduced in this revision, none of which impact the validity of the results.

* One of the key new results introduced in this revision is the finding of synergy via ultrasensitivity of IKK activation. However the section that describes this is a little confusing. This section makes ultrasensitivity synonymous with the hill coefficient, which seems to imply that this is the result of a tuning parameter rather than any emergent ultrasensitivity (or the Hill coefficient as a mechanistically interpretable 'cooperativity' parameter). Perhaps all that is required here is to be a bit more specific as to the mechanism this describes.

The observation of the NFkB oscillations is interesting, but it's not clear what the significance is for synergy. It seems that synergistic cells don't have oscillations, but then a Hill coefficient of 1 also doesn't have oscillations, so it's not clear what the message here is.

Here this discussion would also be improved by briefly mentioning the effect of the Hill coeffcient on the dynamics, where it enters in the model, and what typical values are.

* For the sequestration of CD14, it would also be nice to see a plot of 'free' CD14 over time, or similar, that directly shows the availability of CD14 in both cases, rather than inferring it from the earlier peaking of Pam.

* A description of (or at least a reference to) the the PAWN sensitivity analysis method would be useful.

Minor presentation issues/typos:
* The discussion mentions information theory techniques, but there isn't anything I would describe specifically as information theory in the paper (no mention in entropy, mutual information, etc).
* Add references to TensorFlow and the Random forest methods described in the methods section
* Some missing definitions in the figures (rand para, KS)
* Some typos in the figures and captions, acivation -> activation, indiced -> induced, antaginistic -> antagonistic

Reviewer #2:

The revised manuscript is improved on many fronts compared to the original. The new sections on IKK ultrasensitivity and CD14 mediated antagonism are quite interesting. The original message (title) of the manuscript was 'Macrophages maintain signaling fidelity in response to ligand mixtures'. In the revised version the authors attempt to decipher the mechanism(s) behind the response specificity. While they provide evidence for some cases (pairs of ligands), I am not sure if the mechanism is the main point of this work (i.e. the title). The antagonism (e.g. via unavailability of cofactors or endosomes) could be only relevant if cells are stimulated with all the ligands at the same time. I leave it to the authors to make any changes in this regard.

That said, I think the manuscript will be of interest to a broad audience and is acceptable for MSB in its current version. I can't think of any further changes for significant improvement.

Reviewer #3:

Comments on Macrophage response specificity to ligand mixtures is improved by signaling pathway antagonism by Gou et al.

The authors investigate experimentally single macrophages' NF-kB responses to five ligands, separately and to 3 pairs of ligands. Using single-ligand stimulation, they train an existing mathematical model to replicate single-cell NF-kB nuclear trajectories. The trajectories of the remaining ligand combinations (pairs, triples, quadruples) are obtained in numerical simulations.
After revisions, the paper is sound methodologically and is based on a larger single-cell dataset. As such, it deserves publication. My main problem is related to two abstract-level claims:

1) The stimulus-response specificity (SRS) is partly encoded in the
dynamics of the NFκB transcription factor.
2) NFκB temporal coding can convey the presence of specific ligands within mixtures.

The SRS is assessed either 3 methods:

(1) Wasserstein distance of signaling codon distributions between conditions;
(2) random forest classifier using signaling codons; and
(3) Long Short-Term Memory (LSTM) classifier using time series data.

The second and third approaches are used to calculate the precision, accuracy, and F1 score for a specific ligand or a combination of ligand predictions (Fig. 3C and Fig. S7).

The demonstrated ability to recognize stimuli is based on several key assumptions that are difficult to meet in reality. a) cells know when stimulation is given, b) specific stimulation doses are provided (cells may distinguish a given stimulus from other stimuli if they are present in some doses but not in others, c) stimulation has no temporal pattern, which could strongly influence the temporal pattern of NF-kB activation. The authors only partially addressed these comments.

Additionally, when we look into accuracy and precision for single stimuli (or combinations of stimuli) recognition (Fig. 3C and Fig S7), we may see that possibly less than 1 bit of information about the type of stimuli is transmitted. This is because when K different symbols are sent with equal probabilities, and for each symbol, accuracy = precision = p, mutual information per symbol, $I_K(p)$, equals

$$I_K(p) = \log_2(K) + p \log_2(p) + (1-p) \log_2((1-p)/(K-1)).$$

(assuming that errors are distributed uniformly across the remaining K-1 symbols).

One can easily check that for K=5, 1 bit of information is transmitted when p = 0.75 and for K=10 when p = 0.58 (and less than 1 bit when p is smaller). We may see that for single stimuli, accuracy or precision exceeds 0.75 only for TNF while for other stimuli is below 0.6. For pairs, it is always below 0.5.

This implies that less than 1 bit of information is encoded in the NF-kB profile for stimuli or stimulus pairs. Therefore, saying that the type of stimuli or their combination is encoded in the NF-kB profile is misleading. My (just) private opinion is that cells do not recognize stimuli; if they do, they use more than one transcription factor to do so.

I urge authors to calculate MI (what I did is only a crude estimate) and modify the conclusions accordingly.

Nevertheless, impressed by the approach and the volume of valuable experimental data, I think the study deserves publication, with modified conclusions.

Pre-decision cross-commenting

Reviewer #2

The comments from Reviewer #3 are valid, especially about the mutual information about the mutual information. I am not an expert in information theory but I think both of Reviewer #3's problems can be summarized in the following question: If we are given an unlabeled trajectory of NFkB temporal dynamics, can (or how likely) we deduce the ligand or the combination thereof that generated that trajectory? I think figure 3-D and 3-E partially answer this question but I am not sure if the presence/absence predictions are made only on the trajectories with stimulus containing the corresponding ligand. I think the authors should have an opportunity to comment on this issue. As they already have the data, MI calculation should be straightforward and that may lead to some relevant adjustment to the overall message of the paper.

Reviewer #1

I think that Reviewer 3's comments are reasonable, and agree that it should be reasonably straightforward for the authors to implement the suggestions. However I also agree that to rigorously demonstrate what the authors claim would be considerably more involved. In particular, the use of the term 'temporal coding' without any explanation implies that the signal is somehow 'encoded' into the temporal pattern, where they only indicate an association with these temporal oscillations. This might just be poor choice of wording, as without any claims to this effect the rest of the paper is reasonably solid.

Manuscript Number: MSB-2026-13639-T
Title: Macrophage response specificity to ligand mixtures is improved by signaling pathway antagonism
Author: Xiaolu Guo
Supriya Sen
Julian Gonzalez
Alexander Hoffmann

Dear Alex,

Thank you for submitting your manuscript to Molecular Systems Biology. We have now received the enclosed reports from the three reviewers who evaluated the previous version of the manuscript at Review Commons. As you will see from the comments below, Reviewers #1 and #2 support publication of the manuscript after addressing a number of relatively minor issues. Reviewer #3, however, continues to raise concerns regarding two central claims: (1) that stimulus-response specificity is partly encoded in NF-κB dynamics, and (2) that temporal coding conveys ligand identity.

During our pre-decision cross-commenting process, both Reviewers #1 and #2 acknowledged Reviewer #3's concerns. The reviewers' additional comments are included following their reports.

In light of these comments, our recommendations are as follows:

- You may consider performing mutual information analyses to quantify how much stimulus information is contained in NF-κB dynamics. This analysis could provide additional support for your conclusions but is optional.

Response: We performed mutual information analysis and discussed the results in the Discussion, with a more detailed explanation provided in our response to Reviewer 3. Through this analysis, we realized that mutual information is highly sensitive to perfect or near-perfect ligand discrimination but less sensitive to cases with modest error rates. For example, in a binary classifier, a 0% error rate corresponds to 1 bit of mutual information, whereas a 10% error rate, corresponding to 90% accuracy, reduces the mutual information to approximately 0.5 bits, representing an almost 50% decrease. In addition, 60% accuracy yields mutual information close to 0 bits, indicating that mutual information has limited ability to distinguish classifier performance below this range.

In our analyses, the binary classifiers shown in Appendix Fig. S6C achieved approximately 60–90% accuracy in determining whether a specific ligand was present or absent based on NFκB dynamics across combinatorial stimulation conditions. Consistently, mutual information analysis yielded 0–0.5 bits for these binary classifiers (new Appendix Fig. S15), in agreement with the classifier results. While for our main conclusion, this analysis does not provide additional information beyond the machine-learning classifier analysis shown in Appendix Fig. S6C, or Figure 3D after correction for unbalanced data size, it led to an interesting insight that different metrics are sensitive to different regimes of stimulus-response specificity and can therefore be useful in different scenarios. We therefore included these results in Appendix Fig. S15 and added a discussion of this point in the Discussion.

We also emphasize that the approaches used in our study provide insight into non-perfectly distinguishable cases by addressing: (1) the extent to which the presence or absence of a ligand in a given stimulation condition can be detected from NFκB signaling dynamics; (2) which ligands or stimulation conditions are more distinguishable than others; and (3) what mechanisms may influence these differences. We have revised our conclusions to more precisely describe what is supported by the data.

A more detailed explanation is provided in our response to Reviewer 3.

- Independently, please carefully review the wording of the abstract, conclusions, and other relevant sections to ensure that the claims accurately reflect what the data demonstrate. Particular attention should be paid to terms such as "encode," "recognize," "temporal coding," and "identity," as well as related wording, ensuring that the language aligns precisely with the evidence and does not imply stronger conclusions than are supported by the data.

Response: We thank the editor's suggestion, and we have softened our conclusions:

(Abstract, Line 22) "*Following iterative refinement of the model, we quantified SRS with Wasserstein distance and machine learning classification and found that that NFκB temporal coding can **partially** convey the presence of specific ligands within mixtures*".

(Synopsis, Lines 34-36) *"Guo et al developed computational simulations of single-macrophage NFκB dynamics in response to ligand combinations that revealed that macrophages may **partially** detect the identity of specific ligands within ligand mixtures."*

(Synopsis, Lines 43-44) "*Macrophages NFκB signaling dynamics preserve **partial** stimulus specificity in complex ligand-mixture contexts, and this is fueled by signaling antagonism.*"

(Results, Lines 302-304) "*These results indicated a **notable** capability of preserving ligand-specific information within NFκB dynamics, **potentially** enabling nuclear detection of extracellular ligands even in complex stimulus mixtures.*"

(Discussion, Lines 425-427) "*Our results demonstrate that macrophages maintain **partial** NFκB signaling SRS in complex ligand-mixture contexts.*"

We would respectfully emphasize that "temporal coding" is not a main conclusion of the present study, but rather a well-supported concept established by previous publications (Selimkhanov et al., 2014; Kellogg et al., 2017; Lane et al., 2019; Adelaja et al., 2021; Rahman et al., 2024). Our work and analyses are built upon this established framework. We have revised the introduction to better clarify this point (lines 56-58).

Please also address all other remaining issues raised by the reviewers. As our editorial policy allows a single round of major revision, it is essential that your responses are as complete as possible. We encourage you to take this opportunity to clarify the key claims and ensure that the manuscript's conclusions are fully supported. Please feel free to contact me if you wish to discuss any of the points in further detail.

Response: We have addressed all remaining issues raised by the reviewer. These comments helped us improve the manuscript by more precisely describing our key claims, strengthening the logical flow, and improving the clarity and professionalism of the writing (see responses to reviewers for more details).

On a more editorial level, we would ask you to address the following issues:

- Please provide a .docx formatted version of the manuscript text (including legends for main figures, EV figures and tables). Please make sure that the changes are highlighted to be clearly visible.

Response: We have revised the manuscript text to include figure legends of main and EV figures. Changes are highlighted in blue texts.

- Please provide individual production quality figure files as .eps, .tif, .jpg (one file per figure). When assembling figures, please refer to our figure preparation guideline in order to ensure proper formatting and readability in print as well as on screen: https://media.springernature.com/original/springer-cms/rest/v1/content/27825798/data/v1

Response: We have revised all the main and EV figures to align with the figure preparation guideline.

-Please provide a .docx formatted letter INCLUDING the reviewers' reports and your detailed point-by-point responses to their comments. As part of the EMBO Press transparent editorial process, the point-by-point response is part of the Review Process File (RPF), which will be published alongside your paper.

Response: We have provided point-by-point response letter to reviewers.

-Please note that all corresponding authors are required to supply an ORCID ID for their name upon submission of a revised manuscript.

Response: We would request to add ORCID ID for all authors, and here are the corresponding ORCID:

Xiaolu Guo: 0000-0002-5740-2428

Supriya Sen: 0000-0002-5022-6388

Julian Gonzalez: 0009-0004-2043-7581

Alexander Hoffmann (corresponding author): 0000-0002-5607-3845

-We replaced Supplementary Information with Expanded View (EV) Figures and Tables that are collapsible/expandable online. A maximum of 5 EV Figures can be typeset. EV Figures should be cited as 'Figure EV1, Figure EV2" etc... in the text and their respective legends should be included in the main text after the legends of regular figures.

Response: We have replaced 5 of the Supplementary figures with EV Figures, and labeled them as Figure EV1, EV2, …, EV5 as required. Corresponding legends are included in the main texts.

Additional Tables/Datasets should be labeled and referred to as Table EV1, Dataset EV1, etc. Legends have to be provided in a separate tab in case of .xls files. Alternatively, the legend can be supplied as a separate text file (README) and zipped together with the Table/Dataset file.
Response: We have changed the label for the additional table and dataset, and they are correctly referred in the manuscript.

For the figures and tables that you do NOT wish to display as Expanded View figures, they should be bundled together with their legends in a single PDF file called *Appendix*, which

should start with a short Table of Content. Each legend should be below the corresponding Figure/Table in the Appendix. Appendix figures and tables should be referred to in the main text as: "Appendix Figure S1, Appendix Figure S2, Appendix Table S1" etc. See detailed instructions regarding expanded view here: https://link.springer.com/journal/44320/submission-guidelines#cms-Expanded-View-data.

Response: We have put all additional figures into Appendix PDF file, and added table of context.

-Before submitting your revision, primary datasets (and computer code, where appropriate) produced in this study need to be deposited in an appropriate public database.
Please remember to provide a reviewer password if the datasets are not yet public.
The accession numbers and database should be listed in a formal "Data Availability" section (placed after Materials & Method) that follows the model below (see also https://link.springer.com/partners/embo-press/editorial-policies#Data%20availability%20statement). Please note that the Data Availability Section is restricted to new primary data that are part of this study.
Data availability
The datasets (and computer code) produced in this study are available in the following databases:
- RNA-Seq data: Gene Expression Omnibus GSE46843 (https://www.ncbi.nlm.nih.gov/geo/query/acc.cgi?acc=GSE46843)
- [data type]: [name of the resource] [accession number/identifier/doi] ([URL or identifiers.org/DATABASE:ACCESSION])
*** Note - All links should resolve to a page where the data can be accessed. ***
Response: We have published dataset at Mendeley Data and provided the link in the Data Availability session. DOI: 10.17632/hcg272hzyd.1

- At EMBO Press we ask authors to provide source data for the main manuscript figures. You will receive a separate email with instructions for providing source data with your revised manuscript, including how to upload and organize the files.
Additional information on source data and instruction on how to label the files are available < https://link.springer.com/journal/44320/submission-guidelines#cms-Source-data >
Response: We have organized and provided all Source data as required. We will upload all Source data.

- Our journal encourages inclusion of *data citations in the reference list* to directly cite datasets that were re-used and obtained from public databases. Data citations in the article text are distinct from normal bibliographical citations and should directly link to the database records from which the data can be accessed. In the main text, data citations are formatted as follows: "Data ref: Smith et al, 2001". In the Reference list, data citations must be labeled with "[DATASET]". A data reference must provide the database name, accession number/identifiers and a resolvable link to the landing page from which the data can be accessed at the end of the reference. Further instructions are available at < https://link.springer.com/journal/44320/submission-guidelines#cms-Reference-guidelines >.
Response: We have not re-used published datasets.

- We updated our journal's competing interests policy in January 2022 and request authors to consider both actual and perceived competing interests. Please review the policy

(https://link.springer.com/partners/embo-press/editorial-policies#Competing%20interest%20disclosures) and update your competing interests if necessary.
Please use the heading "Disclosure statement and competing interests".
Response: We have added disclosure statement and competing interests.

- All Materials and Methods need to be described in the main text using our 'Structured Methods' format. According to this format, the Methods section includes a Reagents and Tools Table (listing key reagents, experimental models, software and relevant equipment and including their sources and relevant identifiers) followed by a Methods and Protocols section describing the methods, ideally using a step-by-step protocol format. The aim is to facilitate adoption of the methodologies across labs.

Please download and fill our Reagents and Tools Table template (.docx), which you can find in our author guidelines: https://link.springer.com/journal/44320/submission-guidelines#structuredmethods.

When submitting your revised manuscript, please do not include the Reagents and Tools Table in the Methods section of the manuscript but upload it as a separate file choosing the file type "Reagent Table".

Response: We have provided Reagent and tools table and submitted as a separate file, and structured Method session as required.

-Regarding data quantification:
Please ensure to specify the name of the statistical test used to generate error bars and P values, the number (n) of independent experiments (please specify technical or biological replicates) underlying each data point and the test used to calculate p-values in each figure legend. Discussion of statistical methodology can be reported in the materials and methods section, but figure legends should contain a basic description of n, P and the test applied. Graphs must include a description of the bars and the error bars (s.d., s.e.m.).
Please also include scale bars in all microscopy images.
Response: We have added statistical tests for corresponding Figures (Figs. 2, 4, and 5, as well as Figs. EV4 and EV5), and now report the corresponding p-values. We have also revised the relevant figure legends to include the statistical information. In addition, we added a “Statistics and Reproducibility” section to describe the statistical methodology and provide information on replicates.

- Please provide a "standfirst text" summarizing the study in one or two sentences (approximately 250 characters, including space), three to four "bullet points" highlighting the main findings and a " visual abstract" (550px width and 400-600 px height, PNG format) to highlight the paper on our homepage.
Please refer to published papers for examples.
Response: we have provided the Synopsis (in main text) and Visual abstract.

When you resubmit your manuscript, please download our CHECKLIST (https://media.springernature.com/original/springer-cms/rest/v1/content/27825796/data/v1) and include the completed form in your submission.
Please note that the Author Checklist will be published alongside the paper as part of the

transparent process .
Response: We have completed the CHECKLIST form.

If you feel you can satisfactorily deal with these points and those listed by the referees, you may wish to submit a revised version of your manuscript. Please attach a covering letter giving details of the way in which you have handled each of the points raised by the referees. A revised manuscript may be once again subject to review and you probably understand that we can give you no guarantee at this stage that the eventual outcome will be favorable.
Response: We have included a covering letter giving details of the way in which you have handled each of the points raised by the referees.

I look forward to receiving the revised manuscript soon.

Kind regards,
Jingyi

Jingyi Hou, PhD
Senior Editor
Molecular Systems Biology

We realize that it is difficult to revise to a specific deadline. In the interest of protecting the conceptual advance provided by the work, we recommend a revision within 3 months (22nd Jun 2026). Please discuss the revision progress ahead of this time with the editor if you require more time to complete the revisions. Use the link below to submit your revision:

https://msb.msubmit.net/cgi-bin/main.plex

IMPORTANT: Please read our guidelines for revised submissions:
https://link.springer.com/journal/44320/submission-guidelines#cms-Revised-submissions

*** PLEASE NOTE *** As part of the EMBO Press transparent editorial process initiative (see our Editorial at https://dx.doi.org/10.1038/msb.2010.72), Molecular Systems Biology publishes online a Review Process File with each accepted manuscripts. This file will be published in conjunction with your paper and will include the anonymous referee reports, your point-by-point response and all pertinent correspondence relating to the manuscript. If you do NOT want this File to be published, please inform the editorial office at contact@molsystbiol.org within 14 days upon receipt of the present letter.

Point-by-point responses to reviewers' comments

Reviewer #1:

Many thanks for the updated manuscript and your work in responding to the reviewer comments. I have no further questions related to the previous comments, and I am satisfied that all issues are addressed.

I have several comments on the new material introduced in this revision, none of which impact the validity of the results.

* One of the key new results introduced in this revision is the finding of synergy via ultrasensitivity of IKK activation. However the section that describes this is a little confusing. This section makes ultrasensitivity synonymous with the hill coefficient, which seems to imply that this is the result of a tuning parameter rather than any emergent ultrasensitivity (or the Hill coefficient as a mechanistically interpretable 'cooperativity' parameter). Perhaps all that is required here is to be a bit more specific as to the mechanism this describes.

Response: We thank the reviewer for this helpful comment. We agree that our original description of this section was not sufficiently clear. We do not intend to use the Hill coefficient simply as a tunable fitting parameter or to equate it directly with mechanistic cooperativity. Rather, in this context it serves to represent an ultrasensitive input–output relationship in TAK-mediated IKK activation, which is the property relevant to the emergence of synergy in the model.

More specifically, we found that synergy arises in the low-dose regime. In synergistic cells, upstream parameter settings that produce low IKK activity are also required. Under these conditions, TAK-mediated IKK activation can display a synergistic response when the input–output relationship is ultrasensitive (Hill coefficient = 2), whereas this does not occur in the linear case (Hill coefficient = 1). In this sense, ultrasensitivity in TAK-mediated IKK activation is required for synergy at the level of IKK.

At the same time, synergy at the NFκB level is not determined by IKK ultrasensitivity alone. Its transmission to NFκB also depends on downstream network properties, particularly the dynamical regime of the IKK–IκBα–NFκB module. Our downstream parameter analysis further shows that synergy generated at the IKK level can be propagated to NFκB only when this regulatory network is in a non-oscillatory regime, where IκBα negative feedback does not mask or reshape the upstream synergistic input.

To address this concern, we have revised the Results section (Lines 341-343, 344-350) to clarify this, and to avoid the impression that ultrasensitivity is being introduced merely as a tuning parameter or as equivalent to NFκB response synergy.

The observation of the NFkB oscillations is interesting, but it's not clear what the significance is for synergy. It seems that synergistic cells don't have oscillations, but then a Hill coefficient of 1 also doesn't have oscillations, so it's not clear what the message here is.
Response: We thank the reviewer for this point. We agree that the significance of the oscillatory versus non-oscillatory regime for synergy needed to be clarified. We here distinguish between the generation of synergy and its transmission to NFκB. Ultrasensitivity in TAK-mediated IKK activation (Hill = 2) is required to generate synergy at the level of IKK. On the other hand, the

non-oscillatory regime of the IKK–IκBα–NFκB module is not itself sufficient to create synergy, but it is required for upstream IKK synergy to be transmitted to the NFκB response. Therefore, when the Hill coefficient is 1, the downstream module may still be non-oscillatory, but no upstream IKK synergy is generated and thus no NFκB synergy is observed. We have revised the text to make this distinction more explicit (Lines 342-350).

The reviewer's comment helps us to realize that the paragraph of testing Hill=1 and Hill = 2 for oscillation under TNF condition is confusing and a distraction from the main logic flow (LPS and Pam stimulation). In the revised manuscript, we removed that paragraph, and expanded the paragraph explaining synergy mechanism to focus on the main logic flow (Lines 342-350). Figure S14 is now only inside the parentheses, briefly explain why Hill = 2 in the original model (Adelaja 2021).

Here this discussion would also be improved by briefly mentioning the effect of the Hill coeffcient on the dynamics, where it enters in the model, and what typical values are.

Response: In the revision, we have included an explanation of how the Hill coefficient influences the dynamics of IKK activation. Specifically, we now state that the Hill coefficient enters in the TAK-mediated activation of IKK and controls the steepness of this input–output relationship. In our analysis, a Hill coefficient of 1 represents a linear response (at low dose), whereas a value greater than 1 represents an ultrasensitive response that can enable synergy at low input levels. Specifically, the ultrasensitivity under Hill coefficient of 2 allows wider range of TAK activation inducing low activation IKK, further allowing TNF-stimulated NFκB to be oscillatory, because TNF-induced low TAK activation constrains IKK activation to a low range within the oscillatory regime (Adelaja 2021). Since IKK serves as the input to the IKK–IκBα–NFκB feedback network, the Hill coefficient affects how upstream TAK activity is transmitted into downstream NFκB dynamics. In this context, a Hill coefficient of 2 is a typical value used in the model allowing for TNF-stimulated oscillation.

* For the sequestration of CD14, it would also be nice to see a plot of 'free' CD14 over time, or similar, that directly shows the availability of CD14 in both cases, rather than inferring it from the earlier peaking of Pam.

Response: We thank the reviewer for this comment. In response, we have added plots showing the time course of free CD14, as well as the dynamics of all CD14-bound species. As shown in Appendix Fig. S13, in antagonistic cells, only 42% of the CD14 engaged in the LPS signaling pathway under single-ligand stimulation remains available under the combinatorial ligand condition, compared with 55% in non-antagonistic cells. In contrast, for the Pam signaling pathway, 98%–99% of CD14 engaged under single-ligand stimulation remains available under the combinatorial ligand condition in both antagonistic and non-antagonistic cells. These results further support our conclusion that the antagonism mechanism arises from competition for CD14 between Pam and LPS. We have added the figure in the revision (Lines 389, Appendix Fig. S13).

* A description of (or at least a reference to) the the PAWN sensitivity analysis method would be useful.

Response: In the revision, we have added description of PAWN sensitivity analysis in Method "*Parameter sensitivity analysis*" (Lines 843-851) and added the reference in main text & Method (Lines 331, 379, Lines 843-851).

Minor presentation issues/typos:
* The discussion mentions information theory techniques, but there isn't anything I would describe specifically as information theory in the paper (no mention in entropy, mutual information, etc).

Response: Based on reviewer 3's comment, we have added mutual information results in the discussion (Lines 494-498, Appendix Fig. S15).

* Add references to TensorFlow and the Random forest methods described in the methods section.

Response: We thank the reviewer for the kind reminder, and we have added references for TensorFlow and the Random Forest python package, as well as the original model references (Lines 775-777, 796-797).

* Some missing definitions in the figures (rand para, KS)

Response: We added the missed definitions (Fig. EV4, EV5, and figure legends).

* Some typos in the figures and captions, acivation -> activation, indiced -> induced, antaginistic -> antagonistic

Response: We thank the reviewer for carefully reading our manuscript in detail, and we have fixed all the typos in the figures and captions.

Reviewer #2:

The revised manuscript is improved on many fronts compared to the original. The new sections on IKK ultrasensitivity and CD14 mediated antagonism are quite interesting. The original message (title) of the manuscript was 'Macrophages maintain signaling fidelity in response to ligand mixtures'. In the revised version the authors attempt to decipher the mechanism(s) behind the response specificity. While they provide evidence for some cases (pairs of ligands), I am not sure if the mechanism is the main point of this work (i.e. the title). The antagonism (e.g. via unavailability of cofactors or endosomes) could be only relevant if cells are stimulated with all the ligands at the same time. I leave it to the authors to make any changes in this regard.
Response: We thank the reviewer for the endorsement. and we would respectfully emphasize that current title that antagonism improving the stimulus response specificity more highlighting the finding of the paper leveraging the mechanistic modeling. The previous title might be not clearly determined/specified, especially as reviewer 3 pointed out, whether 70~80 % distinguishability of present-or-not should be considered as maintaining signaling fidelity (compared to random guess, which 50% distinguishability) or not sufficient fidelity (compared with 100% distinguishability). Via modeling and experiments, we identified CpG-pIC antagonism and CD14 competition LPS-Pam antagonism mechanism and found that CpG-pIC antagonism improved the stimulus response specificity. The antagonism may happen when CpG and pIC co-stimulated or LPS and Pam co-occurrence, not necessarily when all ligands presented.

That said, I think the manuscript will be of interest to a broad audience and is acceptable for

MSB in its current version. I can't think of any further changes for significant improvement.

Response: We thank the reviewer for this generous endorsement.

Reviewer #3:

Comments on Macrophage response specificity to ligand mixtures is improved by signaling pathway antagonism by Gou et al.

The authors investigate experimentally single macrophages' NF-kB responses to five ligands, separately and to 3 pairs of ligands. Using single-ligand stimulation, they train an existing mathematical model to replicate single-cell NF-kB nuclear trajectories. The trajectories of the remaining ligand combinations (pairs, triples, quadruples) are obtained in numerical simulations. After revisions, the paper is sound methodologically and is based on a larger single-cell dataset. As such, it deserves publication. My main problem is related to two abstract-level claims:

1) The stimulus-response specificity (SRS) is partly encoded in the
dynamics of the NFκB transcription factor.

Response: The reviewer's comment helped us to revise the introduction and avoid the confusion of temporal coding as a conclusion of this paper. In general, this conclusion that SRS is partially encoded in dynamics of transcription factor is not a main claims from this paper, but a well-supported conclusion in a series of previous publications over a decade (Selimkhanov et al., 2014; Kellogg et al., 2017; Lane et al., 2019; Adelaja et al., 2021; Rahman et al., 2024). Specifically, NFkB dynamics by itself also encodes stimuli information (Ade 2021, Science 2014) in context of single ligands. Our work is built on these rigorously and extensively supported works. In the revision, we have further clarified temporal coding concepts as previous findings in the introduction (Lines 56-58), which is one basis of our work. We hope this can avoid the confusion of this being our claims.

"The temporal dynamics of nuclear NFκB activity has been shown to mediate SRS following exposure of macrophages to cytokines or immune threats , a phenomenon known as temporal coding in signaling pathways (Selimkhanov et al., 2014; Kellogg et al., 2017; Lane et al., 2019; Adelaja et al., 2021; Rahman et al., 2024)."

For the reviewer's reference, we have also included selected studies and corresponding figures that support temporal coding by transcription factors and signaling networks, including temporal coding by NFκB dynamics.

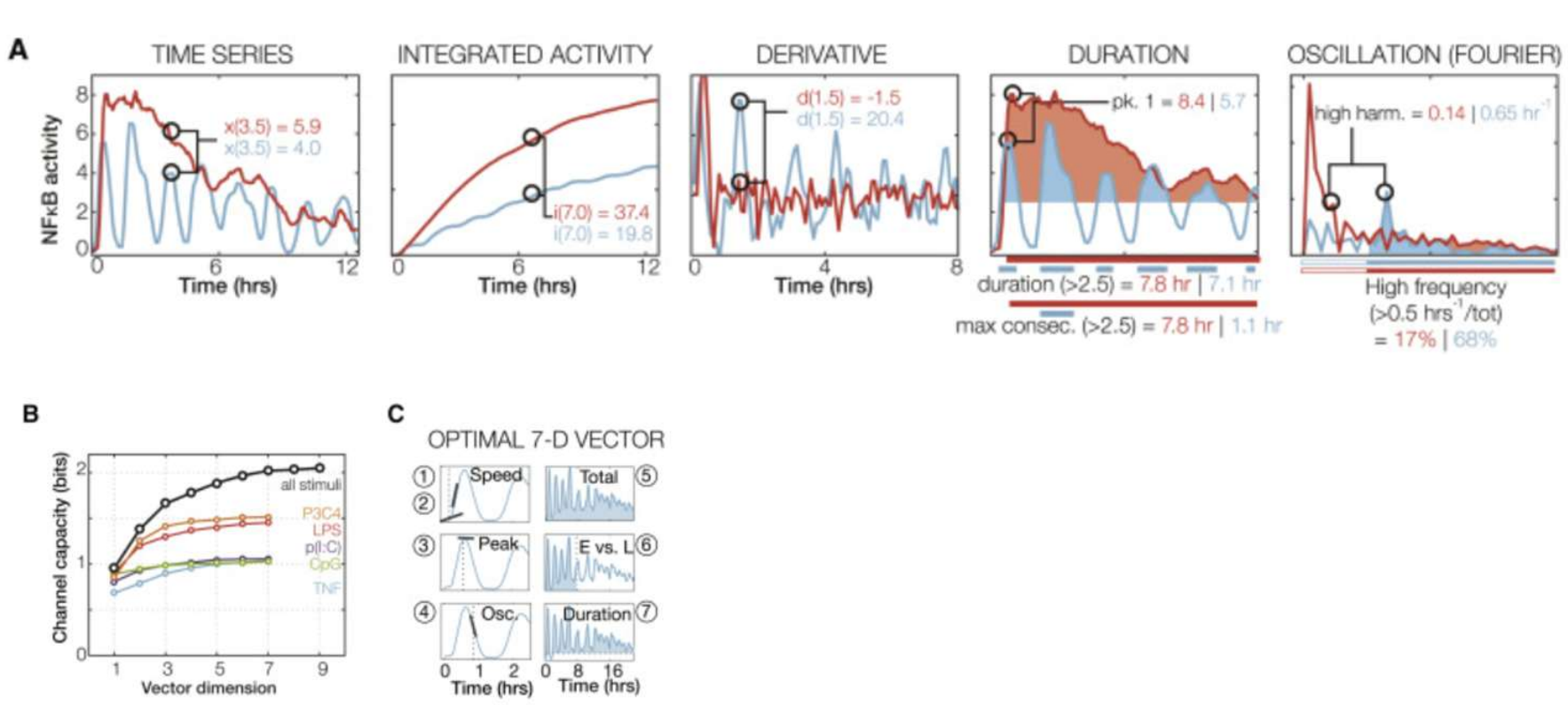

Adelaja et al. (2021, *Immunity*, Figure 2A–C). Figure 2A illustrates representative dynamic features extracted from NFκB trajectories, including time series, integrated activity, derivative, duration, and oscillatory content. Figure 2B shows channel capacity calculated from combinations of NFκB dynamic features for ligand-specific dose-response discrimination. Figure 2C further identifies the most informative NFκB dynamical features, termed “signaling codons,” that convey stimulus information.

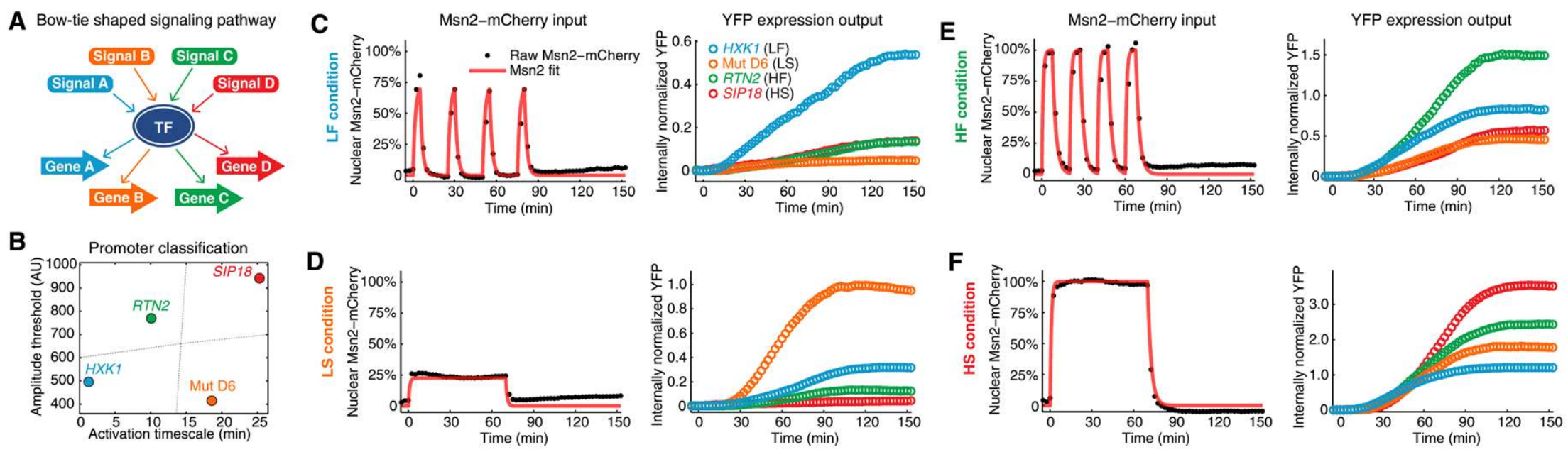

Hansen and O’Shea (2016, *Current Biology*, Figure 1). Figure 1C–F illustrates how different temporal activation dynamics of the yeast transcription factor Msn2 can be decoded by downstream promoters into distinct gene-expression programs. In these panels, the left plots show Msn2-mCherry input dynamics, whereas the right plots show the corresponding YFP reporter expression outputs from different promoters.

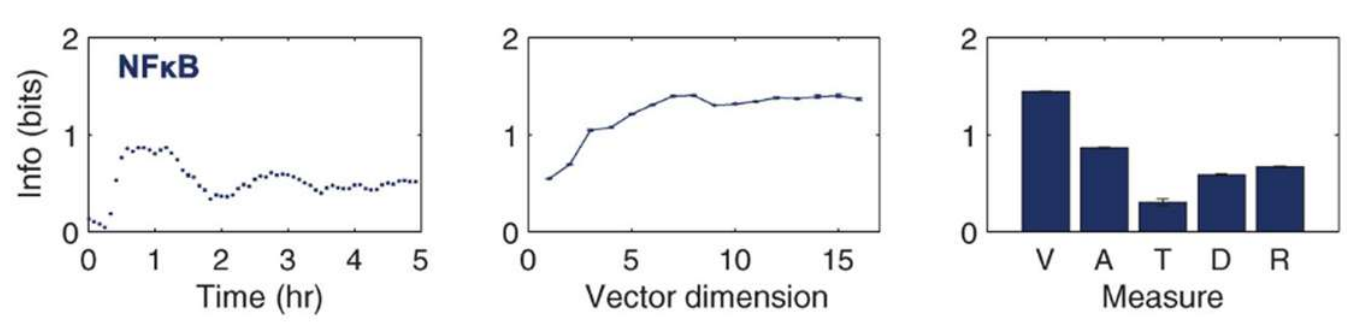

Fig. 2 Information transmission capacity of static and dynamic ERK, Ca^{2+}, and NF-κB responses.
(**A**) Information transmission capacity calculated from static scalar response distribution based on single–time point measurements. (**B**) Information transmission capacity calculated from multivariate dynamic responses as a function of the dimension of the multivariate vector. The multivariate vector was subsampled using a uniform grid centered on the middle time point (fig. S19). (**C**) Comparison of the multivariate vector (V) measurement to the following scalar responses: maximum response amplitude (A), maximum response time (T), maximal rate of response (D), ratio of maximum response amplitude to initial response amplitude (R). Error bars are SEMs from six biological replicates for ERK and four for Ca^{2+}, and SDs from five jackknife iterations for NF-κB (tables S1 to S3). The multivariate vector information transfer was significantly greater than all scalar measures ($P < 0.05$, Student’s *t* test, table S6).

Selimkhanov et al. (2014, *Science*, Figure 2). Figure 2B shows that multivariate dynamic responses encode greater information transmission capacity than static single-time-point measurements for ERK, Ca^{2+}, and NFκB signaling. Figure 2C compares information transmission from the full dynamic response vector with scalar dynamic features, showing that NFκB dynamic trajectories can encode more stimulus information than individual summary features alone.

2) NFκB temporal coding can convey the presence of specific ligands within mixtures.
Response: In the revision, we have softened this conclusion.

The SRS is assessed either 3 methods:

(1) Wasserstein distance of signaling codon distributions between conditions;
(2) random forest classifier using signaling codons; and
(3) Long Short-Term Memory (LSTM) classifier using time series data.

The second and third approaches are used to calculate the precision, accuracy, and F1 score for a specific ligand or a combination of ligand predictions (Fig. 3C and Fig. S7).

The demonstrated ability to recognize stimuli is based on several key assumptions that are difficult to meet in reality. a) cells know when stimulation is given, b) specific stimulation doses are provided (cells may distinguish a given stimulus from other stimuli if they are present in some doses but not in others, c) stimulation has no temporal pattern, which could strongly influence the temporal pattern of NF-kB activation. The authors only partially addressed these comments.
Response: We thank the reviewer for this valuable comment, we would gently push back on these comments.
a) the cell naturally sensing the stimuli via receptor-ligand binding; thus, cells can detect, via the changes of receptor-ligand abundance, the stimulation/changes of environmental cues; different ligand receptor complex formulation evolving different mechanisms, thus allowing for NFkB response priority/timing differences, thus introducing different gene expression at different stages. We would emphasize that cells can detect timing of stimuli is not an assumption, instead it is a naturally process of how cell processing environmental cues.
b) Yes, this is an assumption. Previous studies have demonstrated that the highest ligand doses produce the most distinguishable responses. We also believe this assumption is physiologically relevant, because cells must remain robust to negligible immune threats while retaining the capacity to distinguish critical immune challenges rather than noise. In our framework, we represent such critical threats as high doses of individual ligands or their mixtures. We have now clarified this as an assumption and explained this in the modeling explanation (Lines 242-245).
c) We have responded to this point in the previous revision, and we have appended the corresponding response here: “We agree with the reviewer that ligands could be given at different times. Considering time delays between ligands (the inset and also removal) dramatically adds to the combinatorial complexity. Some initial studies by the Tay lab are beginning to explore some scenarios of time-shifted ligand pairs (Wang et al 2025). Here we focus on a systematic exploration of all ligand combinations at 6 different doses. The fact that we do not consider time delays is not an assumption but admittedly a limitation that may well be addressed in future studies. We have included a brief discussion of this issue in the discussion (Lines 518-529). We would like to suggest that despite (or maybe because) limiting our study to coincident stimuli, we made some noteworthy discoveries. ”

Additionally, when we look into accuracy and precision for single stimuli (or combinations of stimuli) recognition (Fig. 3C and Fig S7), we may see that possibly less than 1 bit of information about the type of stimuli is transmitted. This is because when K different symbols are sent with equal probabilities, and for each symbol, accuracy = precision = p, mutual information per symbol, I_K(p), equals

I_K(p) = log_2(K) + p log_2(p) + (1-p) log_2((1-p)/(K-1)).

(assuming that errors are distributed uniformly across the remaining K-1 symbols).

One can easily check that for K=5, 1 bit of information is transmitted when p = 0.75 and for K=10 when p = 0.58 (and less than 1 bit when p is smaller). We may see that for single stimuli, accuracy or precision exceeds 0.75 only for TNF while for other stimuli is below 0.6. For pairs, it is always below 0.5.

This implies that less than 1 bit of information is encoded in the NF-kB profile for stimuli or stimulus pairs. Therefore, saying that the type of stimuli or their combination is encoded in the NF-kB profile is misleading. My (just) private opinion is that cells do not recognize stimuli; if they do, they use more than one transcription factor to do so.

I urge authors to calculate MI (what I did is only a crude estimate) and modify the conclusions accordingly.

Response: We appreciate the reviewer's insightful comment, which helps improve the accuracy and clarity of our writing and to extend discussion insights. We agree that our results do not demonstrate that macrophages exhibit perfect SRS, in the sense of distinguishing all stimuli perfectly. Therefore, we have revised the manuscript to soften our conclusions and avoid this potential confusion:

(Abstract, Line 22) "*Following iterative refinement of the model, we quantified SRS with Wasserstein distance and machine learning classification and found that that NFκB temporal coding can* ***partially*** *convey the presence of specific ligands within mixtures*".

(Synopsis, Lines 43-44) "*Macrophages NFκB signaling dynamics preserve* ***partial*** *stimulus specificity in complex ligand-mixture contexts, and this is fueled by signaling antagonism.*"

(Results, Lines 302-304) "*These results indicated a* ***notable*** *capability of preserving ligand-specific information within NFκB dynamics,* ***potentially*** *enabling nuclear detection of extracellular ligands even in complex stimulus mixtures.*"

(Discussion, Lines 425-426) "*Our results demonstrate that macrophages maintain* ***partial*** *NFκB signaling SRS in complex ligand-mixture contexts.*"

We also calculated mutual information as suggested (Ross, 2014). When considering the five single-ligand conditions together, the estimated channel capacity (maximal MI) was 1.3116 bits. When considering all 31 stimulation conditions, the estimated channel capacity was 1.6691 bits.

The MI calculated here is higher than 1 bit because classifier performance is strongly affected by the number of conditions included in the classification task, with performance decreasing as the number of total conditions increases (Adelaja et al., 2021). Similarly, in our case, the classifier performance for the five single-ligand conditions was substantially lower when the classifier was trained across all 31 conditions (Figure 3C in main text) than when it was trained only on the five single-ligand conditions (Figures below). Therefore, although the reviewer's estimate may provide a reasonable approximation under specific assumptions, our calculation shows that including the five ligand conditions together with the untreated condition can yield an MI value greater than 1 bit.

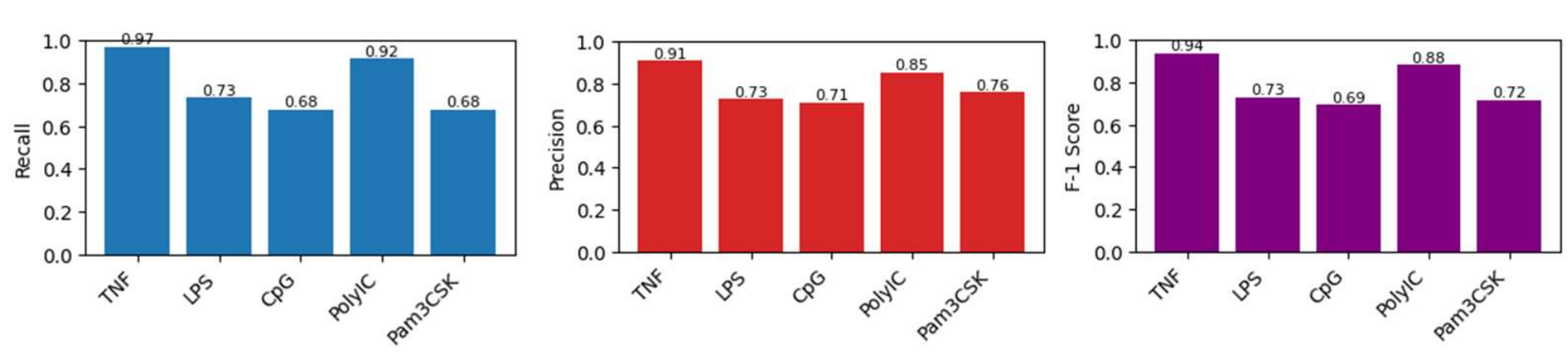

Figure. Classifier performance when trained on five single-ligand conditions. Bar plots show the performance of classifiers trained to identify five single-ligand stimulation conditions based on NFκB dynamic features. Recall, precision, and F1 score are shown for each ligand condition: TNF, LPS, CpG, polyIC, and Pam3CSK.

Regarding whether additional signaling pathways could compensate for stimulus recognition when quantified by channel capacity, the previous study by Luecke et al. reported approximately 0.70–0.74 bits of information for four single-ligand conditions and showed that inclusion of p38 provided little enhancement of SRS, increasing it to 0.74–0.79 bits (Luecke et al., 2024; Figure 3) (For reviewer's reference, we also put the figure below).

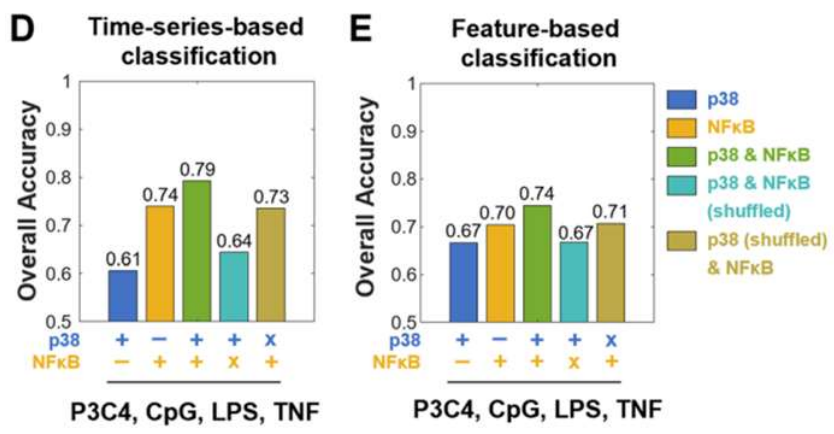

The reviewer's comments prompted us to carefully consider the mutual information (MI) metric and its relationship to machine-learning classifier performance. MI or channel capacity analysis can provide an overall assessment of whether all conditions are separable, and such metrics strongly reward (highly sensitive for) near-perfect classification. For example, considering a two-category classifier, based on the channel capacity formula for symmetric channels (also given by the reviewer) (Cover & Thomas, 2012), a 0% error rate corresponds to 1 bit of channel capacity. However, even a 1% error rate reduces the channel capacity to approximately 0.92 bits; a 5% error rate reduces it to approximately 0.71 bits; a 10% error rate, corresponding to 90% accuracy, reduces it to approximately 0.53 bits; and 80% accuracy corresponds to approximately 0.28 bits. Thus, MI metrics are highly sensitive for evaluating perfectly or near-perfectly distinguishable conditions but have limited sensitivity for assessing differences in distinguishability among imperfectly classified cases.

Error rate ε	Accuracy	Capacity C (bits/use)
1%	99%	0.919
5%	95%	0.714
10%	90%	0.531
20%	80%	0.278
40%	60%	0.029

We then calculated the channel capacity (Ross, 2014) for the binary classifier "present or not" for each ligand:

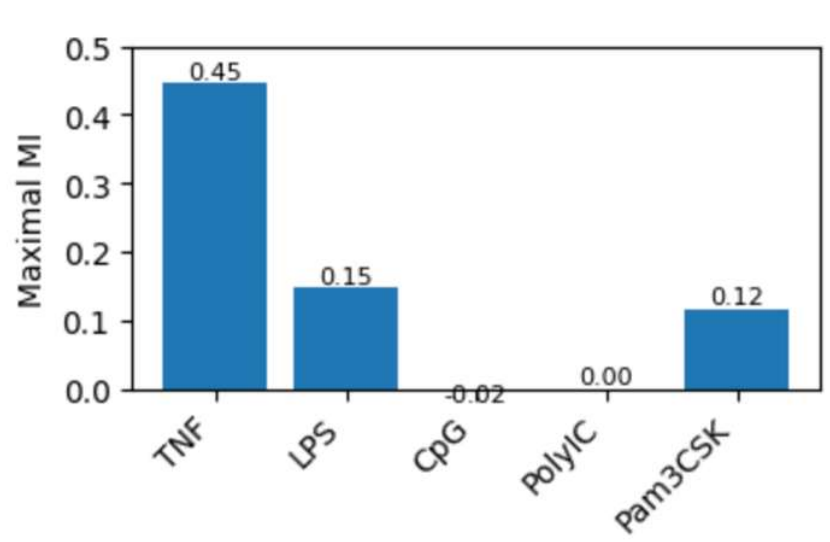

The results further illustrate that MI strongly favors near-perfect discrimination. For example, the TNF present-versus-absent classifier achieved 89% prediction accuracy (Appendix Fig. S6C), corresponding to a maximal MI, optimized over all possible input distributions, of 0.45 bits. In contrast, classifiers with lower accuracies showed substantially smaller MI values: the LPS

present-versus-absent and Pam present-versus-absent classifiers, both with accuracies below 80%, yielded maximal MI values of 0.12 bits and 0.15 bits, respectively. Classifiers with approximately 60% accuracy had MI values close to 0 bits. Thus, MI is highly sensitive to near-perfect distinguishability but has limited dynamic range for distinguishing among imperfectly classified cases. The reviewer's comments prompted us to examine this issue more carefully, leading to this interesting observation, which we have now added on supplementary figure (Appendix Fig. S15) and included in the Discussion section (Lines 494-498).

In the revised manuscript, we now use more cautious language, such as "partially distinguishable," to clarify this point. We would emphasize that to determine which ligands or specific stimulation conditions are better recognized than others, and to identify the mechanisms that may influence these differences, the analysis we did is appropriate. Mutual information, while providing global measure of classification performance, is less suited to answering such condition-specific mechanistic questions.

Nevertheless, impressed by the approach and the volume of valuable experimental data, I think the study deserves publication, with modified conclusions.
Response: we thank the reviewer for carefully reading our manuscript and the valuable comments. As reviewer suggested, we have modified our conclusions in abstract, synopsis, introduction, and results, to more accurately describe what the data shows (Lines 22, 34-36, 43-44, 302-304, 425-426).

Pre-decision cross-commenting

Reviewer #2

The comments from Reviewer #3 are valid, especially about the mutual information about the mutual information. I am not an expert in information theory but I think both of Reviewer #3's problems can be summarized in the following question: If we are given an unlabeled trajectory of NFkB temporal dynamics, can (or how likely) we deduce the ligand or the combination thereof that generated that trajectory? I think figure 3-D and 3-E partially answer this question but I am not sure if the presence/absence predictions are made only on the trajectories with stimulus containing the corresponding ligand. I think the authors should have an opportunity to comment on this issue. As they already have the data, MI calculation should be straightforward and that may lead to some relevant adjustment to the overall message of the paper.
Response: We thank the reviewer for these comments, we have responded to Reviewer #3's comments (please refer to responses Reviewer 3).

We totally agree with the reviewer's summary of the questions, whether macrophage can determine specific ligand is presented or not based on the NFkB trajectory, and also, if yes, to what extent. All Figure 3 is aiming for answering that question, using different approaches. For Figure 3D and Figure 3E, the presence/absence predictions are made on the trajectories for all ligand mixtures, not only on the trajectories with stimulus containing the corresponding ligand. All ligand mixtures are classified into two categories: ligand-presented, ligand-not-presented. This binary present-or-not classifiers model a fundamental function of macrophages: determining whether specific immune threats are present in a complex environment (Lavin et al., 2015; Sheu & Hoffmann, 2022).

As we mentioned in response to Reviewer 3, MI metrics strongly reward near-perfect classification. For example, for a two-category classifier, for a two-category classifier, a 0% error rate corresponds to 1 bit of channel capacity. However, even just a 10% error rate, corresponding to 90% accuracy, reduces it to approximately 0.5 bits; and 70% accuracy corresponds to approximately 0.1 bits. As what we expected, calculation of MI for these binary classifiers led to 0~0.5 bits or less for each ligand (Appendix Fig. S15). For more details, please refer to response to Reviewer 3.

We realized that our previous conclusion may cause confusion of implying perfect SRS, and we have now softened our conclusions:

(Abstract, Line 22) "*Following iterative refinement of the model, we quantified SRS with Wasserstein distance and machine learning classification and found that that NFκB temporal coding can* ***partially*** *convey the presence of specific ligands within mixtures*".

(Synopsis, Lines 45-46) "*Macrophages NFκB signaling dynamics preserve* ***partial*** *stimulus specificity in complex ligand-mixture contexts, and this is fueled by signaling antagonism.*"

(Results, Lines 300-302) "*These results indicated a* ***notable*** *capability of preserving ligand-specific information within NFκB dynamics,* ***potentially*** *enabling nuclear detection of extracellular ligands even in complex stimulus mixtures.*"

(Discussion, Lines 417-418) "*Our results demonstrate that macrophages maintain partial NFκB signaling SRS in complex ligand-mixture contexts.*"

Reviewer #1

I think that Reviewer 3's comments are reasonable, and agree that it should be reasonably straightforward for the authors to implement the suggestions. However I also agree that to rigorously demonstrate what the authors claim would be considerably more involved. In particular, the use of the term 'temporal coding' without any explanation implies that the signal is somehow 'encoded' into the temporal pattern, where they only indicate an association with these temporal oscillations. This might just be poor choice of wording, as without any claims to this effect the rest of the paper is reasonably solid.

Response: We thank the reviewer for this comment. To address Reviewer 3's concern regarding this point, in the Introduction, we have revised introduction (Lines 56–58) to summarize previous studies on temporal coding in NFκB signaling (Selimkhanov et al., 2014; Kellogg et al., 2017; Lane et al., 2019; Adelaja et al., 2021; Rahman et al., 2024) and clarified this as previous literature conclusion. As the reviewer notes, temporal coding is not a conclusion of the present study. Rather, it is an established concept from prior work that has been rigorously demonstrated in the literature.

We also thank the reviewer for their generous endorsement of our work.

References

Adelaja, A., Taylor, B., Sheu, K. M., Liu, Y., Luecke, S., & Hoffmann, A. (2021). Six distinct NFκB signaling codons convey discrete information to distinguish stimuli and enable appropriate macrophage responses. *Immunity*, *54*(5), Article 5. https://doi.org/10.1016/j.immuni.2021.04.011

Cover, T. M., & Thomas, J. A. (2012). *Elements of Information Theory*. Wiley.

Kellogg, R. A., Tian, C., Etzrodt, M., & Tay, S. (2017). Cellular Decision Making by Non-Integrative Processing of TLR Inputs. *Cell Reports*, *19*(1), Article 1. https://doi.org/10.1016/j.celrep.2017.03.027

Lane, K., Andres-Terre, M., Kudo, T., Monack, D. M., & Covert, M. W. (2019). Escalating Threat Levels of Bacterial Infection Can Be Discriminated by Distinct MAPK and NF-κB Signaling Dynamics in Single Host Cells. *Cell Systems*, *8*(3), Article 3. https://doi.org/10.1016/j.cels.2019.02.008

Lavin, Y., Mortha, A., Rahman, A., & Merad, M. (2015). Regulation of macrophage development and function in peripheral tissues. *Nature Reviews. Immunology*, *15*(12), 731–744. https://doi.org/10.1038/nri3920

Rahman, S. M. T., Singh, A., Lowe, S., Aqdas, M., Jiang, K., Vaidehi Narayanan, H., Hoffmann, A., & Sung, M.-H. (2024). Co-imaging of RelA and c-Rel reveals features of NF-κB signaling for ligand discrimination. *Cell Reports*, *43*(3), 113940. https://doi.org/10.1016/j.celrep.2024.113940

Ross, B. C. (2014). Mutual Information between Discrete and Continuous Data Sets. *PLOS ONE*, *9*(2), e87357. https://doi.org/10.1371/journal.pone.0087357

Selimkhanov, J., Taylor, B., Yao, J., Pilko, A., Albeck, J., Hoffmann, A., Tsimring, L., & Wollman, R. (2014). Accurate Information Transmission Through Dynamic

Biochemical Signaling Networks. *Science (New York, N.Y.)*, *346*(6215), Article 6215. https://doi.org/10.1126/science.1254933

Sheu, K. M., & Hoffmann, A. (2022). Functional Hallmarks of Healthy Macrophage Responses: Their Regulatory Basis and Disease Relevance. *Annual Review of Immunology*, *40*, 295–321. https://doi.org/10.1146/annurev-immunol-101320-031555

9th Jun 2026

Manuscript Number: MSB-2026-13639R
Title: Macrophage response specificity to ligand mixtures is improved by signaling pathway antagonism
Author: Xiaolu Guo
Supriya Sen
Julian Gonzalez
Alexander Hoffmann

Dear Alex,

Thank you for submitting the revised version of your manuscript. We are satisfied with the revisions made and would be able to accept your manuscript, pending the following editorial-level issues:

1. The table on page 20 requires a label and callout. We suggest labeling it as Appendix Table S1. It should therefore be removed from the manuscript file and added to the appendix file, with an appropriate legend and correct callout.

2. The entire section titled "SUPPLEMENTARY DATASET" should be removed from the manuscript file.

3. The following funding information is missing from the submission system and should be added: UCLA QCBio Collaboratory Fellowship.

4. The references need to be formatted according to the Molecular Systems Biology reference style. Please list up to 10 co-authors of a paper before adding et al. in the reference list. Citations should be listed in alphabetical order.

5. Appendix:
- The appendix file must be submitted in PDF format.
- The title page should read "Appendix for [manuscript title]" (instead of "Supplementary Information + manuscript title"); author and affiliation lists are not required.
- All figures and tables should be labeled consistently as Appendix Figure Sx and Appendix Table Sx throughout the manuscript and appendix PDF.
- Highlights should be removed from the text, as the appendix PDF will not be typeset.

6. "Code availability" should be incorporated into the "Data Availability" section, and the separate "Code Availability" section heading should be removed.

7. We have recently updated the dimension requirements for the visual abstract, and I apologize for the inconvenience. Please provide an updated version in PNG format with exact dimensions of 550 px (width) × 300 px (height), if possible, for optimal dispay on the website.

8. Please remove the synopsis text from the manuscript file and submit it as a separate Word document.

9. The current Table EV1 is rather complex and should be converted to an EV dataset. Therefore, the source file name, title, legend, and manuscript callout should all be updated to Dataset EV2 instead of Table EV1. The legend should remain uploaded as a separate tab/sheet in the Excel file, as it already is.

10. Source Data: Source data files need to be saved in a scheme one figure/folder and then uploaded as .zip files. E.g. all the Source data files for figure 1 need to be saved in a single folder and this needs to be zipped and then uploaded as "SD figure 1.zip" file. Completed SD checklist should be uploaded as Related Manuscript File.

11. Figure callouts:
- There is no panel F in Figure 5, although it is cited in the figure legend; please resolve this discrepancy.
-The callout for Supplementary Dataset 1 should be renamed Dataset EV1.

12. In the "Disclosure and competing interests statement," please add the following sentence: " AH is an editorial advisory board member. This has no bearing on the editorial consideration of this article for publication."

13. Please note that information related to n is missing in the legends 2C,F; EV-1B; EV-2B, and should be added.

Use the following link to submit your revised paper:

https://msb.msubmit.net/cgi-bin/main.plex

As a matter of course, please make sure that you have correctly followed the instructions for authors as given on the submission website.

Thank you for submitting this paper to Molecular Systems Biology.

Kind regards,
Jingyi

Jingyi Hou, PhD
Senior Editor
Molecular Systems Biology

If you do choose to resubmit, please click on the link below to submit the revision online before 9th Jul 2026.

https://msb.msubmit.net/cgi-bin/main.plex

*** PLEASE NOTE *** As part of the EMBO Press transparent editorial process initiative (see our Editorial at https://dx.doi.org/10.1038/msb.2010.72 , Molecular Systems Biology will publish online a Review Process File to accompany accepted manuscripts. When preparing your letter of response, please be aware that in the event of acceptance, your cover letter/point-by-point document will be included as part of this File, which will be available to the scientific community. More information about this initiative is available in our Instructions to Authors. If you have any questions about this initiative, please contact the editorial office (msb@embo.org).

Manuscript Number: MSB-2026-13639R
Title: Macrophage response specificity to ligand mixtures is improved by signaling pathway antagonism
Author: Xiaolu Guo
Supriya Sen
Julian Gonzalez
Alexander Hoffmann

Dear Alex,

Thank you for submitting the revised version of your manuscript. We are satisfied with the revisions made and would be able to accept your manuscript, pending the following editorial-level issues:
Response: Dear Jingyi, thank you for the constructive editorial assessment. We have addressed each point below. All changes in the manuscript file are marked in blue font.

1. The table on page 20 requires a label and callout. We suggest labeling it as Appendix Table S1. It should therefore be removed from the manuscript file and added to the appendix file, with an appropriate legend and correct callout.

Response: We have removed the dose table from the manuscript and added it to the Appendix file as “Appendix Table S1” with a descriptive legend. The in-text reference now reads “...the dose values are listed in Appendix Table S1.”

2. The entire section titled "SUPPLEMENTARY DATASET" should be removed from the manuscript file.

Response: The entire SUPPLEMENTARY DATASET section has been deleted from the manuscript file.

3. The following funding information is missing from the submission system and should be added: UCLA QCBio Collaboratory Fellowship.
Response: We have added the acknowledgements of “UCLA QCBio Collaboratory Fellowship” in the online submission system.

4. The references need to be formatted according to the Molecular Systems Biology reference style. Please list up to 10 co-authors of a paper before adding et al. in the reference list. Citations should be listed in alphabetical order.
Response: We have updated the reference list format according to the Molecular Systems Biology reference style.

5. Appendix:
(a)- The appendix file must be submitted in PDF format.
(b)- The title page should read "Appendix for [manuscript title]" (instead of "Supplementary Information + manuscript title"); author and affiliation lists are not required.
(c)- All figures and tables should be labeled consistently as Appendix Figure Sx and Appendix Table Sx throughout the manuscript and appendix PDF.
(d)- Highlights should be removed from the text, as the appendix PDF will not be typeset.
Response: (a) The Appendix is now provided in PDF format (Appendix_ 0614.pdf). (b) We have revised the title page in format of "Appendix for [manuscript title]"; the author and affiliation lists have been removed. (c) All figures are labeled "Appendix Figure Sx" throughout the appendix and the manuscript (manuscript callouts changed from "Appendix Fig." to "Appendix Figure"), and the moved dose table is labeled "Appendix Table S1." (d) Highlights have been removed from the appendix text.

6. "Code availability" should be incorporated into the "Data Availability" section, and the separate "Code Availability" section heading should be removed.
Response: We have incorporated the code-availability sentence into the Data Availability paragraph and removed the separate "Code Availability" heading.

7. We have recently updated the dimension requirements for the visual abstract, and I apologize for the inconvenience. Please provide an updated version in PNG format with exact dimensions of 550 px (width) × 300 px (height), if possible, for optimal dispay on the website.
Response: We have updated the visual abstract based on the new requirement.

8. Please remove the synopsis text from the manuscript file and submit it as a separate Word document.
Response: We have removed the synopsis from the manuscript and provided it as a separate Word document (Synopsis.docx).

9. The current Table EV1 is rather complex and should be converted to an EV dataset. Therefore, the source file name, title, legend, and manuscript callout should all be updated to Dataset EV2 instead of Table EV1. The legend should remain uploaded as a separate tab/sheet in the Excel file, as it already is.
Response: We have renamed Table_EV1.xlsx source file as Dataset_EV2.xlsx; its internal title/legend now reads "Dataset EV2."; the manuscript callout has been changed from "Table EV1" to "Dataset EV2." The legend remains on its own separate "legends" sheet, as before.

10. Source Data: Source data files need to be saved in a scheme one figure/folder and then uploaded as .zip files. E.g. all the Source data files for figure 1 need to be saved in a single folder and this needs to be zipped and then uploaded as "SD figure 1.zip" file. Completed SD checklist should be uploaded as Related Manuscript File.

Response: We have organized source data one folder per figure and zipped them as "SD figure 1.zip" … "SD figure 5.zip" (in the SD_zips folder). The completed SD Checklist (SD_checklist_Completed.xlsx) has been uploaded as a Related Manuscript File (author-completed form).

11. Figure callouts:
(a) - There is no panel F in Figure 5, although it is cited in the figure legend; please resolve this discrepancy.
(b) -The callout for Supplementary Dataset 1 should be renamed Dataset EV1.
Response: (a) In the last version of Figure 5, we have added new panel F, and we verified the current figure — panel F (the MDS visualization) is present in Figure 5 and is consistent with legend entry F and the in-text callout (Fig. 5F); the figure now contains six panels A–F, matching the legend, so the discrepancy is resolved. (b) We have renamed "Supplementary dataset 1" as "Dataset EV1."

12. In the "Disclosure and competing interests statement," please add the following sentence: " AH is an editorial advisory board member. This has no bearing on the editorial consideration of this article for publication."
Response: We thank the editor for this kind reminder, and we have added the sentence.

13. Please note that information related to n is missing in the legends 2C,F; EV-1B; EV-2B, and should be added.
Response: We have added sample size information to the corresponding panel (revised legends in blue text): for the simulated distributions, n = 1,000 single cells per condition; for the experimental distributions in Fig. 2C, n ≈ 600–950 single cells per condition (exact n per condition provided in the Source Data).

Use the following link to submit your revised paper:

https://msb.msubmit.net/cgi-bin/main.plex

As a matter of course, please make sure that you have correctly followed the instructions for authors as given on the submission website.

Thank you for submitting this paper to Molecular Systems Biology.

Kind regards,
Jingyi

Jingyi Hou, PhD
Senior Editor

Molecular Systems Biology

If you do choose to resubmit, please click on the link below to submit the revision online before 9th Jul 2026.

https://msb.msubmit.net/cgi-bin/main.plex

*** PLEASE NOTE *** As part of the EMBO Press transparent editorial process initiative (see our Editorial at https://dx.doi.org/10.1038/msb.2010.72 , Molecular Systems Biology will publish online a Review Process File to accompany accepted manuscripts. When preparing your letter of response, please be aware that in the event of acceptance, your cover letter/point-by-point document will be included as part of this File, which will be available to the scientific community. More information about this initiative is available in our Instructions to Authors. If you have any questions about this initiative, please contact the editorial office (msb@embo.org).

18th Jun 2026
Manuscript number: MSB-2026-13639RR
Title: Macrophage response specificity to ligand mixtures is improved by signaling pathway antagonism

Dear Alex,

Thank you again for sending us your revised manuscript. We are now satisfied with the modifications made and I am pleased to inform you that your paper has been accepted for publication.

Your manuscript will be processed for publication by EMBO Press. It will be copy edited and you will receive page proofs prior to publication. Please note that you will be contacted by Springer Nature Author Services to complete licensing and payment information.

You may qualify for financial assistance for your publication charges - either via a Springer Nature fully open access agreement or an EMBO initiative. Check your eligibility: https://link.springer.com/journal/44320/how-to-publish-with-us

Should you be planning a Press Release on your article, please notify the production team via this form in order to coordinate publication and release dates: https://forms.cloud.microsoft/e/eH4A8JVP6g

If you have any questions, please do not hesitate to contact the Editorial Office. Thank you for your contribution to Molecular Systems Biology.

Kind regards,
Jingyi

Jingyi Hou, PhD
Senior Editor
Molecular Systems Biology

>>> Please note that it is Molecular Systems Biology policy for the transcript of the editorial process (containing referee reports and your response letter) to be published as an online supplement to each paper. If you do NOT want this, you will need to inform the Editorial Office via email immediately. More information is available here: https://link.springer.com/partners/embo-press/editorial-policies#Peer%20review

***
Rev_Com_number: RC-2025-02980
New_manu_number: MSB-2026-13639RR
Corr_author: Hoffmann
Title: Macrophage response specificity to ligand mixtures is improved by signaling pathway antagonism

EMBO Press Author Checklist

Corresponding Author Name: Alexander Hoffmann	
Journal Submitted to: Molecular Systems Biology	
Manuscript Number: MSB-2026-13639-T	

USEFUL LINKS FOR COMPLETING THIS FORM
The EMBO Journal - Author Guidelines
EMBO Reports - Author Guidelines
Molecular Systems Biology - Author Guidelines
EMBO Molecular Medicine - Author Guidelines

Reporting Checklist for Life Science Articles (updated January
This checklist is adapted from Materials Design Analysis Reporting (MDAR) Checklist for Authors. MDAR establishes a minimum set of requirements in transparent reporting in the life sciences (see Statement of Task: 10.31222/osf.io/9sm4x). Please follow the journal's guidelines in preparing your manuscript.
Please note that a copy of this checklist will be published alongside your article.

Abridged guidelines for figures
1. Data
The data shown in figures should satisfy the following conditions:
- the data were obtained and processed according to the field's best practice and are presented to reflect the results of the experiments in an accurate and unbiased manner.
- ideally, figure panels should include only measurements that are directly comparable to each other and obtained with the same assay.
- plots include clearly labeled error bars for independent experiments and sample sizes. Unless justified, error bars should not be shown for technical
- if n<5, the individual data points from each experiment should be plotted. Any statistical test employed should be justified.
- Source Data should be included to report the data underlying figures according to the guidelines set out in the authorship guidelines on Data

2. Captions
Each figure caption should contain the following information, for each panel where they are relevant:
- a specification of the experimental system investigated (eg cell line, species name).
- the assay(s) and method(s) used to carry out the reported observations and measurements.
- an explicit mention of the biological and chemical entity(ies) that are being measured.
- an explicit mention of the biological and chemical entity(ies) that are altered/varied/perturbed in a controlled manner.
- the exact sample size (n) for each experimental group/condition, given as a number, not a range;
- a description of the sample collection allowing the reader to understand whether the samples represent technical or biological replicates (including how many animals, litters, cultures, etc.).
- a statement of how many times the experiment shown was independently replicated in the laboratory.
- definitions of statistical methods and measures:
  - common tests, such as t-test (please specify whether paired vs. unpaired), simple χ2 tests, Wilcoxon and Mann-Whitney tests, can be unambiguously identified by name only, but more complex techniques should be described in the methods section;
  - are tests one-sided or two-sided?
  - are there adjustments for multiple comparisons?
  - exact statistical test results, e.g., P values = x but not P values < x;
  - definition of 'center values' as median or average;
  - definition of error bars as s.d. or s.e.m.

Please complete ALL of the questions below.
Select "Not Applicable" only when the requested information is not relevant for your study.

Materials

Newly Created Materials	Information included in the manuscript?	In which section is the information available? (Reagents and Tools Table, Materials and Methods, Figures, Data Availability Section)
New materials and reagents need to be available; do any restrictions apply?	Yes	Data Availability Section, Reagents and Tools Table

Antibodies	Information included in the manuscript?	In which section is the information available? (Reagents and Tools Table, Materials and Methods, Figures, Data Availability Section)
For antibodies provide the following information:<br>- Commercial antibodies: RRID (if possible) or supplier name, catalogue number and or/clone number<br>- Non-commercial: RRID or citation	Not Applicable	

DNA and RNA sequences	Information included in the manuscript?	In which section is the information available? (Reagents and Tools Table, Materials and Methods, Figures, Data Availability Section)
Short novel DNA or RNA including primers, probes: provide the sequences.	Not Applicable	

Cell materials	Information included in the manuscript?	In which section is the information available? (Reagents and Tools Table, Materials and Methods, Figures, Data Availability Section)
**Cell lines: Provide species information, strain. Provide accession number in repository OR supplier name, catalog number, clone number, and/OR RRID.**	Yes	Reagents and Tools Table
**Primary cultures: Provide species, strain, sex of origin, genetic modification status.**	Yes	Reagents and Tools Table, Materials and Methods
Report if the cell lines were recently authenticated (e.g., by STR profiling) and tested for mycoplasma contamination.	Not Applicable	

Experimental animals	Information included in the manuscript?	In which section is the information available? (Reagents and Tools Table, Materials and Methods, Figures, Data Availability Section)
**Laboratory animals or Model organisms: Provide species, strain, sex, age, genetic modification status. Provide accession number in repository OR supplier name, catalog number, clone number, OR RRID.**	Yes	Reagents and Tools Table, Materials and Methods
**Animal observed in or captured from the field: Provide species, sex, and age where possible.**	Not Applicable	
Please detail housing and husbandry conditions.	Not Applicable	

Plants and microbes	Information included in the manuscript?	In which section is the information available? (Reagents and Tools Table, Materials and Methods, Figures, Data Availability Section)
**Plants: provide species and strain, ecotype and cultivar where relevant, unique accession number if available, and source (including location for collected wild specimens).**	Not Applicable	
**Microbes: provide species and strain, unique accession number if available, and source.**	Not Applicable	

Human research participants	Information included in the manuscript?	In which section is the information available? (Reagents and Tools Table, Materials and Methods, Figures, Data Availability Section)
If collected and within the bounds of privacy constraints report on age, sex and gender or ethnicity for all study participants.	Not Applicable	

Core facilities	Information included in the manuscript?	In which section is the information available? (Reagents and Tools Table, Materials and Methods, Figures, Data Availability Section)
If your work benefited from core facilities, was their service mentioned in the acknowledgments section?	Not Applicable	

Design

Study protocol	Information included in the manuscript?	In which section is the information available? (Reagents and Tools Table, Materials and Methods, Figures, Data Availability Section)
If study protocol has been pre-registered, provide DOI in the manuscript. For clinical trials, provide the trial registration number OR cite DOI.	Not Applicable	
Report the clinical trial registration number (at ClinicalTrials.gov or equivalent), where applicable.	Not Applicable	

Laboratory protocol	Information included in the manuscript?	In which section is the information available? (Reagents and Tools Table, Materials and Methods, Figures, Data Availability Section)
Provide DOI OR other citation details if external detailed step-by-step protocols are available.	Yes	Materials and Methods

Experimental study design and statistics	Information included in the manuscript?	In which section is the information available? (Reagents and Tools Table, Materials and Methods, Figures, Data Availability Section)
Include a statement about sample size estimate even if no statistical methods were used.	Yes	Materials and Methods, Statistics & Reproducibility
Were any steps taken to minimize the effects of subjective bias when allocating animals/samples to treatment (e.g. randomization procedure)? If yes, have they been described?	Yes	Materials and Methods, Statistics & Reproducibility
Include a statement about blinding even if no blinding was done.	Yes	Materials and Methods, Statistics & Reproducibility
Describe inclusion/exclusion criteria if samples or animals were excluded from the analysis. Were the criteria pre-established?<br><br>If sample or data points were omitted from analysis, report if this was due to attrition or intentional exclusion and provide justification.	Yes	Materials and Methods, Statistics & Reproducibility
For every figure, are statistical tests justified as appropriate? Do the data meet the assumptions of the tests (e.g., normal distribution)? Describe any methods used to assess it. Is there an estimate of variation within each group of data? Is the variance similar between the groups that are being statistically compared?	Yes	Materials and Methods, Statistics & Reproducibility, Figures

Sample definition and in-laboratory replication	Information included in the manuscript?	In which section is the information available? (Reagents and Tools Table, Materials and Methods, Figures, Data Availability Section)
In the figure legends: state number of times the experiment was replicated in laboratory.	Yes	Figures
In the figure legends: define whether data describe technical or biological replicates.	Yes	Figures

Ethics

Ethics	Information included in the manuscript?	In which section is the information available? (Reagents and Tools Table, Materials and Methods, Figures, Data Availability Section)
Studies involving human participants: State details of authority granting ethics approval (IRB or equivalent committee(s), provide reference number for approval.	Not Applicable	
Studies involving human participants: Include a statement confirming that informed consent was obtained from all subjects and that the experiments conformed to the principles set out in the WMA Declaration of Helsinki and the Department of Health and Human Services Belmont Report.	Not Applicable	
Studies involving human participants: For publication of patient photos, include a statement confirming that consent to publish was obtained.	Not Applicable	
Studies involving experimental animals: State details of authority granting ethics approval (IRB or equivalent committee(s), provide reference number for approval. Include a statement of compliance with ethical regulations.	Not Applicable	
Studies involving specimen and field samples: State if relevant permits obtained, provide details of authority approving study; if none were required, explain why.	Not Applicable	

Dual Use Research of Concern (DURC)	Information included in the manuscript?	In which section is the information available? (Reagents and Tools Table, Materials and Methods, Figures, Data Availability Section)
Could your study fall under dual use research restrictions? Please check biosecurity documents and list of select agents and toxins (CDC): https://www.selectagents.gov/sat/list.htm	Not Applicable	
If you used a select agent, is the security level of the lab appropriate and reported in the manuscript?	Not Applicable	
If a study is subject to dual use research of concern regulations, is the name of the authority granting approval and reference number for the regulatory approval provided in the manuscript?	Not Applicable	

Reporting

The MDAR framework recommends adoption of discipline-specific guidelines, established and endorsed through community initiatives. Journals have their own policy about requiring specific guidelines and recommendations to complement MDAR.

Adherence to community standards	Information included in the manuscript?	In which section is the information available? (Reagents and Tools Table, Materials and Methods, Figures, Data Availability Section)
State if relevant guidelines or checklists (e.g., ICMJE, MIBBI, ARRIVE, PRISMA) have been followed or provided.	Not Applicable	
For tumor marker prognostic studies, we recommend that you follow the REMARK reporting guidelines (see link list at top right). See author guidelines, under 'Reporting Guidelines'. Please confirm you have followed these guidelines.	Not Applicable	
For phase II and III randomized controlled trials, please refer to the CONSORT flow diagram (see link list at top right) and submit the CONSORT checklist (see link list at top right) with your submission. See author guidelines, under 'Reporting Guidelines'. Please confirm you have submitted this list.	Not Applicable	

Data Availability

Data availability	Information included in the manuscript?	In which section is the information available? (Reagents and Tools Table, Materials and Methods, Figures, Data Availability Section)
Have primary datasets been deposited according to the journal's guidelines (see 'Data Deposition' section) and the respective accession numbers provided in the Data Availability Section?	Yes	Data Availability Section
Were human clinical and genomic datasets deposited in a public access-controlled repository in accordance to ethical obligations to the patients and to the applicable consent agreement?	Not Applicable	
Are computational models that are central and integral to a study available without restrictions in a machine-readable form? Were the relevant accession numbers or links provided?	Yes	Data Availability Section, Reagents and Tools Table
If publicly available data were reused, provide the respective data citations in the reference list.	Yes	Materials and Methods
